# Supplementary material for: A More Sustainable Isocyanide Synthesis from N-Substituted Formamides Using Phosphorus Oxychloride in the Presence of Triethylamine as Solvent
Source: Molecules. 2022 Oct 13;27(20):6850. doi: 10.3390/molecules27206850 (PMC9608669; doi:10.3390/molecules27206850)
Supplement: Supplementary file 1 [file molecules-27-06850-s001.zip › molecules-1959763-supplementary.pdf]

A More Sustainable Isocyanide Synthesis from *N*-substituted Formamides using Phosphorus oxychloride in the presence of triethylamine as solvent.

Sodeeq Aderotimi Salami [1,*], Xavier Siwe-Noundou [2,*] and Rui Werner Maçedo Krause [1,*]

[1] Department of Chemistry, Rhodes University, Grahamstown 6140, South Africa

[2] Department of Pharmaceutical Sciences, School of Pharmacy, Sefako Makgatho Health Sciences University, Pretoria 0204, South Africa

* Correspondence: sodeeqaderotimi@gmail.com; Tel.: +27 83 302 3511 (S.A.S.); xavier.siwenoundou@smu.ac.za; Tel +27 12 521 5647 (X.S.-N.); r.krause@ru.ac.za; Tel +27 46 603 7030 (R.W.M.K)

Abstract: A simple, green, and highly efficient protocol for the synthesis of isocyanides is described. The reaction involves dehydration of formamides with phosphorus oxychloride in the presence of triethylamine at 0°C under solvent free conditions. The product isocyanides were obtained in high to excellent yields in less than 5 minutes. The method offers several advantages including increased synthesis speed, relatively mild conditions, and rapid access to large numbers of functionalized isocyanides, excellent purity, increased safety, and minimal reaction waste. The new approach of dehydrative isocyanides from formamides is significantly more environmentally friendly than prior methods.

Keywords: *N*-substituted formamides; isocyanides; phosphorus oxychloride, solvent free.

Table of Contents

Page

1. **Experimental** **S2**

1.2. General Procedure **S2**

1.3. General procedure for the passerini product **S12**

2. **Spectral data of synthsized compounds** **S13**
3. **E-factor evaluation and other parameters** **S49**

1. A General Procedure for *N*-Formylation of Amines with Triethyl Orthoformate Promoted by Immobilized H_2SO_4 on Silica Gel

To a mixture of aniline (0.548 mL, 6 mmol) and triethyl orthoformate (24 mmol), the immobilized H2SO4 on silica gel (1.2 g) was then added and the reaction mixture was stirred under reflux conditions (65 ◦C). Progress of the reaction was monitored by TLC. After completion of the reaction, the mixture was diluted with EtOAc (20 mL), filtered, water (30 mL) was added, the solution was extracted with EtOAc, and the combined organic layers were dried over anhydrous Na_2SO_4 and concentrated. The residue was subjected to column chromatography and eluted with (EtOAc–Pet Ether (3:1)) to afford the product in high yields.

Note: This have been previously studied [31]. Formamide molecules have both a conformational stereogenic axis and a configurational stereogenic centre. These molecules take on two distinct syn and anti-conformational diastereomers due to restricted rotation around the Ar–N bond. The 1H and 13C NMR spectra of most of the synthesized formamides were consistent with the presence of two rotamers. Therefore, the formamides were used unresolved in the synthesis of isocyanides.

1.2 Synthesis of Isocyanide from *N*-Formamide

1.2 General Procedure

To a solution of *N*-(3-bromophenyl)formamide (0.25 ml 2 mmol) in triethylamine (2 ml) and subsequently phosphorus oxychloride (2 mmol) 0.2 ml were added at 0 °C. The reaction mixture was stirred for about 5 minutes. The progress of the reaction was monitored by TLC. After the completion of reaction, the reaction mixture was poured directly into a dry packed column. 100 % diethyl ether was used as the mobile phase to afford the product in high yield with minimal solvent consumption. The products obtained were known compounds and identified by melting point and ^{1}H NMR spectroscopy and the spectral data were compared with the literature values [19, 23, 26, 27].

1.2.1 1-bromo-3-isocyanobenzene

To a solution of *N*-(3-bromophenyl)formamide (0.25 ml 2 mmol) in triethylamine (2 ml) and subsequently phosphorus oxychloride (2 mmol) 0.2 ml were added at 0 °C. The reaction mixture was stirred for about 5 minutes. The progress of the reaction was monitored by TLC. After the completion of reaction, the reaction mixture was poured directly into a dry packed column. 100 % diethyl ether was used as the mobile phase to afford a brown liquid 1-bromo-3-isocyanobenzene (0.235 g 94%). IR (NaCl): 2125 cm^{-1}. 1H NMR (400 MHz, $CDCl_3$) δ 7.51 (s, 1H), 7.51 – 7.46 (m, 3H), 7.32 – 7.23 (m, 3H).^{13}C NMR (101 MHz, $CDCl_3$) δ 166.41, 133.01, 131.00, 129.66, 127.92, 125.38, 122.97.

1.2.2 4-bromo-1- isocyanobenzene

To a solution of *N*-(4-bromo phenyl)formamide (0.4g; 2 mmol) in triethylamine (2 ml) and subsequently phosphorus oxychloride (2 mmol) 0.2 ml were added at 0 °C. The reaction mixture was stirred for about 5 minutes. The progress of the reaction was monitored by TLC. After the completion of reaction, the reaction mixture was poured directly into a dry packed column. 100 % diethyl ether was used as the mobile phase to afford a yellow solid **4-bromo phenyl isocyanide** (0.35 g; 85%) m.p 98-100 ◦ C. IR (NaCl): 2114 cm^{-1}. ^{1}H NMR (400 MHz, $CDCl_3$) δ 7.46 (d, *J* = 8.8 Hz, 1H), 7.18 (d, *J* = 8.6 Hz, 1H). ^{13}C NMR (101 MHz, $CDCl_3$) δ 165.65, 132.54, 127.31, 125.20, 123.17

1.2.3 1-chloro-2-isocyanobenzene

To a solution of *N*-(2-chlorophenyl)formamide (0.3g; 2 mmol) in triethylamine (2 ml) and subsequently phosphorus oxychloride (2 mmol) 0.2 ml were added at 0 °C. The reaction mixture was stirred for about 5 minutes. The progress of the reaction was monitored by TLC. After the completion of reaction, the reaction mixture was poured directly into a dry packed column. 100 % diethyl ether was used as the mobile phase to afford brown liquid **1-chloro-2-isocyanobenzene** (0.27 g; 90%). IR (NaCl): 2141 cm^{-}

[1]. 1H NMR (400 MHz, CDCl3) δ 7.40 (dd, J = 7.9, 1.4 Hz, 1H), 7.35 (dd, J = 7.7, 1.4 Hz, 1H), 7.27 (td, J = 7.7, 1.9 Hz, 1H), 7.22 (td, J = 7.6, 1.5 Hz, 1H).^{13}C NMR (101 MHz, $CDCl_3$) δ 169.18, 130.79, 130.31, 130.14, 127.99, 127.57.

1.2.4 1-bromo-2-isocyanobenzene

To a solution of *N*-(2-bromophenyl)formamide (0.4g; 2 mmol) in triethylamine (2 ml) and subsequently phosphorus oxychloride (2 mmol) 0.2 ml were added at 0 °C. The reaction mixture was stirred for about 5 minutes. The progress of the reaction was monitored by TLC. After the completion of reaction, the reaction mixture was poured directly into a dry packed column. 100 % diethyl ether was used as the mobile phase to afford a yellow liquid **1-bromo-2-isocyanobenzene** (0.34 g; 86%). IR (NaCl): 2145 cm^{-1}. ^{1}H NMR (400 MHz, $CDCl_3$) δ 7.57 (dd, J = 8.0, 1.3 Hz, 1H), 7.35 (dd, J = 7.9, 1.3 Hz, 1H), 7.27 (td, J = 7.7, 1.4 Hz, 1H), 7.19 (td, J = 7.8, 1.7 Hz, 1H). ^{13}C NMR (101 MHz, $CDCl_3$) δ 169.22, 133.89, 130.72, 128.24, 119.87.

1.2.5 1-iodo-2-isocyanobenzene

To a solution of *N*-(2-iodophenyl)formamide (0.49 g; 2 mmol) in triethylamine (2 ml) and subsequently phosphorus oxychloride (2 mmol) 0.2 ml were added at 0 °C. The reaction mixture was stirred for about 5 minutes. The progress of the reaction was monitored by TLC. After the completion of reaction, the reaction mixture was poured directly into a dry packed column. 100 % diethyl ether was used as the mobile phase to afford a green liquid **1-iodo-2-isocyanobenzene** (0.36 g; 83%). IR (NaCl): 2121 cm^{-1}. ^{1}H NMR (400 MHz, $CDCl_3$) δ 7.74 (d, J = 8.2 Hz, 1H), 7.24 (dd, J = 6.1, 5.1 Hz, 2H), 6.98 (ddd, J = 8.2, 5.9, 3.3 Hz, 1H). ^{13}C NMR (101 MHz, $CDCl_3$) δ 167.39, 139.37, 130.64, 128.84, 127.53, 94.21.

1.2.6 1-fluoro-3-isocyanobenzene

To a solution of *N*-(3-fluorophenyl)formamide (0.28 g; 2 mmol) in triethylamine (2 ml) and subsequently phosphorus oxychloride (2 mmol) 0.2 ml were added at 0 °C. The reaction mixture was stirred for about 5 minutes. The progress of the reaction was monitored by TLC. After the completion of reaction, the reaction mixture was poured directly into a dry packed column. 100 % diethyl ether was used as the mobile phase to afford a yellow liquid **1-fluoro-3-isocyanobenzene** (0.24 g; 85%). IR (NaCl): 2106 cm^{-1}. ^{1}H NMR (400 MHz, $CDCl_3$) δ 7.38 (td, J = 8.1, 6.0 Hz, 1H), 7.19 (d, J = 8.0 Hz, 1H), 7.16 (dd, J = 2.5, 0.8 Hz, 1H), 7.15 – 7.08 (m, 2H). ^{13}C NMR (101 MHz, $CDCl_3$) δ 166.07, 164.64, 161.85, 131.74, 123.37, 117.76, 114.63.

1.2.7 1,2-difluoro-4-isocyanobenzene

To a solution of *N*-(3,4-difluorophenyl)formamide (0.314 g; 2 mmol) in triethylamine (2 ml) and subsequently phosphorus oxychloride (2 mmol) 0.2 ml were added at 0 °C. The reaction mixture was

stirred for about 5 minutes. The progress of the reaction was monitored by TLC. After the completion of reaction, the reaction mixture was poured directly into a dry packed column. 100 % diethyl ether was used as the mobile phase to afford a yellow liquid **1,2-difluoro-4-isocyanobenzene** (0.25 g; 79%). IR (NaCl): 2137 cm^{-1}. ^{1}H NMR (400 MHz, $CDCl_3$) δ 7.30 (d, *J* = 1.9 Hz, 1H), 7.28 (s, 2H), 7.24 (d, *J* = 1.7 Hz, 1H), 7.23 (s, 1H), 7.22 (s, 2H). ^{13}C NMR (101 MHz, $CDCl_3$) δ 165.42, 151.96, 151.75, 149.35, 149.15, 123.04, 122.97, 122.93, 118.11, 117.92, 116.06, 115.85.

1.2.8 1,2-dichloro-4-isocyanobenzene

To a solution of *N*-(3,4-dichlorophenyl)formamide (0.38 g; 2 mmol) in triethylamine (2 ml) and subsequently phosphorus oxychloride (2 mmol) 0.2 ml were added at 0 °C. The reaction mixture was stirred for about 5 minutes. The progress of the reaction was monitored by TLC. After the completion of reaction, the reaction mixture was poured directly into a dry packed column. 100 % diethyl ether was used as the mobile phase to afford a yellow solid **1,2-dichloro-4-isocyanobenzene** (0.24 g; 85%) m.p 32-33 ◦C. IR (NaCl): 2125 cm^{-1}. ^{1}H NMR (400 MHz, $CDCl_3$) δ 7.28 (d, *J* = 3.4 Hz, 1H), 7.03 (dd, *J* = 8.6, 2.2 Hz, 1H). ^{13}C NMR (101 MHz, DMSO) δ 162.69, 160.30, 139.59, 138.90, 130.55, 127.38, 123.90, 122.14, 120.00, 118.63, 112.32, 111.26.

1.2.9 1-Isocyano-2-nitrobenzene

To a solution of *N*-(2-nitrophenyl)formamide (0.33g; 2 mmol) in triethylamine (2 ml) and subsequently phosphorus oxychloride (2 mmol) 0.2 ml were added at 0 °C. The reaction mixture was stirred for about 5 minutes. The progress of the reaction was monitored by TLC. After the completion of reaction, the reaction mixture was poured directly into a dry packed column. 100 % diethyl ether was used as the mobile phase to afford a yellow liquid **1-Isocyano-2-nitrobenzene** (0.29 g; 87%). IR (NaCl): 2121 cm^{-1}.^{1}H NMR (400 MHz, $CDCl_3$) δ 8.16 – 8.06 (m, 2H), 7.72 (t, *J* = 8.4 Hz, 1H), 7.66 – 7.53 (m, 2H), 7.42 – 7.31 (m, 1H), 6.81 (d, *J* = 8.4 Hz, 1H), 6.74 – 6.65 (m, 1H). ^{13}C NMR (101 MHz, $CDCl_3$) δ 173.70, 144.70, 135.61, 134.53, 129.94, 126.14, 118.41, 116.70.

1.2.10 4-Nitro phenyl isocyanide

To a solution of *N*-(4-Nitrophenyl)formamide (0.33g; 2 mmol) in triethylamine (2 ml) and subsequently phosphorus oxychloride (2 mmol) 0.2 ml were added at 0 °C. The reaction mixture was stirred for about 5 minutes. The progress of the reaction was monitored by TLC. After the completion of reaction, the reaction mixture was poured directly into a dry packed column. 100 % diethyl ether was used as the mobile phase to afford a yellow solid **4-Nitro phenyl isocyanide** (0.31 94%) m.p 96-98 ◦C. IR (NaCl): 2117 cm^{-1}. ^{1}H NMR (400 MHz, MeOD) δ 7.98 (d, *J* = 12.3 Hz, 1H), 6.62 (d, *J* = 10.3 Hz, 1H). ^{13}C NMR (101 MHz, MeOD) δ 156.42, 138.18, 127.36, 113.03.

1.2.11 4-methyl-2-nitro phenyl isocyanide

To a solution of *N*-(4-methyl-2-nitrophenyl)formamide (0.36 g; 2 mmol) in triethylamine (2 ml) and subsequently phosphorus oxychloride (2 mmol) 0.2 ml were added at 0 °C. The reaction mixture was stirred for about 15 minutes. The progress of the reaction was monitored by TLC. After the completion of reaction, the reaction mixture was poured directly into a dry packed column. 100 % diethyl ether was used as the mobile phase to afford a yellow solid **4-methyl-2-nitro phenyl isocyanide** (0.31 g; 86%). IR (NaCl): 2127 cm^{-1}. 1H NMR (400 MHz, CDCl3) δ 7.89 (s, 1H), 7.50 – 7.46 (m, 2H), 6.73 (d, J = 8.5 Hz, 1H), 2.48 (s, 3H). ^{13}C NMR (101 MHz, $CDCl_3$) δ 172.59, 143.43, 142.76, 141.32, 136.73, 134.32, 129.35, 126.19, 125.52, 124.83, 118.88, 20.85, 19.49.

1.2.12 2-isocyano-4-nitrophenol

To a solution of 2-formamido 4-nitrophenol (0.36 g; 2 mmol) in triethylamine (2 ml) and subsequently phosphorus oxychloride (2 mmol) 0.2 ml were added at 0 °C. The reaction mixture was stirred for about 5 minutes. The progress of the reaction was monitored by TLC. After the completion of reaction, the reaction mixture was poured directly into a dry packed column. 100 % diethyl ether was used as the mobile phase to afford a yellow solid **2-isocyano-4-nitrophenol** (0.33 g; 92%). ^{1}H NMR (400 MHz, $CDCl_3$) δ 8.72 (s, 1H), 8.39 (d, *J* = 8.9 Hz, 1H), 8.28 (s, 1H), 7.73 (d, *J* = 9.0 Hz, 1H). ^{13}C NMR (101 MHz, $CDCl_3$) δ 155.24, 153.91, 145.46, 140.96, 122.35, 117.84, 111.85.

1.2.13 1-isocyano-4-methylbenzene

To a solution of *p*-tolyformamide (0.27 g; 2 mmol) in triethylamine (2 ml) and subsequently phosphorus oxychloride (2 mmol) 0.2 ml were added at 0 °C. The reaction mixture was stirred for about 5 minutes. The progress of the reaction was monitored by TLC. After the completion of reaction, the reaction mixture was poured directly into a dry packed column. 100 % diethyl ether was used as the mobile phase to afford a brown solid **1-isocyano-4-methylbenzene** (0.24 g; 88%) m.p 20-21 ◦C. IR (NaCl): 2125 cm^{-1}.^{1}H NMR (400 MHz, $CDCl_3$) δ 7.26 (d, *J* = 8.1 Hz, 1H), 7.18 (d, *J* = 8.2 Hz, 1H), 2.37 (s, 2H). ^{13}C NMR (101 MHz, $CDCl_3$) δ 163.63, 139.44, 130.02, 126.53, 29.98, 21.90.

1.2.14 1-isocyano-3-methylbenzene

To a solution of *m*-tolyformamide (0.27 g; 2 mmol) in triethylamine (2 ml) and subsequently phosphorus oxychloride (2 mmol) 0.2 ml were added at 0 °C. The reaction mixture was stirred for about 5 minutes. The progress of the reaction was monitored by TLC. After the completion of reaction, the reaction mixture was poured directly into a dry packed column. 100 % diethyl ether was used as the mobile phase to afford a brown liquid **1-isocyano-3-methylbenzene** (0.20 g; 74%). IR (NaCl): 2133 cm^{-1}. ^{1}H NMR (400 MHz, $CDCl_3$) δ 7.31 – 7.24 (m, 1H), 7.19 (t, *J* = 8.3 Hz, 2H), 2.36 (s, 2H), 1.25 (s,

1H). ^{13}C NMR (101 MHz, $CDCl_3$) δ 163.90, 140.04, 130.55, 129.49, 127.43, 123.92, 30.48, 21.65.

1.2.15 3,5-dimethyl phenyl isocyanide

To a solution of 3,5-dimethyl phenyl formamide (0.30 g; 2 mmol) in triethylamine (2 ml) and subsequently phosphorus oxychloride (2 mmol) 0.2 ml were added at 0 °C. The reaction mixture was stirred for about 5 minutes. The progress of the reaction was monitored by TLC. After the completion of reaction, the reaction mixture was poured directly into a dry packed column. 100 % diethyl ether was used as the mobile phase to afford a yellow liquid **3,5-dimethyl phenyl isocyanide** (0.24 g; 80%). IR (NaCl): 2157 cm^{-1}. ^{1}H NMR (400 MHz, $CDCl_3$) δ 7.01 (s, 1H), 6.99 (s, 2H), 2.31 (s, 6H). ^{13}C NMR (101 MHz, $CDCl_3$) δ 163.35, 139.56, 131.51, 124.42, 29.69, 21.63.

1.2.16 2-isocyano-1,3,5-trimethylbenzene

To a solution of 2,4,6-trimethylphenyl formamide (0.32 g; 2 mmol) in triethylamine (2 ml) and subsequently phosphorus oxychloride (2 mmol) 0.2 ml were added at 0 °C. The reaction mixture was stirred for about 5 minutes. The progress of the reaction was monitored by TLC. After the completion of reaction, the reaction mixture was poured directly into a dry packed column. 100 % diethyl ether was used as the mobile phase to afford a colourless liquid **2-isocyano-1,3,5-trimethylbenzene** (0.31 g 97%). IR (NaCl): 2121 cm^{-1}. ^{1}H NMR (400 MHz, $CDCl_3$) δ 6.89 (s, 1H), 2.36 (s, 3H), 2.29 (s, 2H). ^{13}C NMR (101 MHz, $CDCl_3$) δ 167.47, 139.13, 134.55, 128.61, 21.50, 19.10.

1.2.17 3-isocyanobenzonitrile

To a solution of 3-formamido benzonitrile (0.29 g; 2 mmol) in triethylamine (2 ml) and subsequently phosphorus oxychloride (2 mmol) 0.2 ml were added at 0 °C. The reaction mixture was stirred for about 5 minutes. The progress of the reaction was monitored by TLC. After the completion of reaction, the reaction mixture was poured directly into a dry packed column. 100 % diethyl ether was used as the mobile phase to afford a white solid **3-isocyanobenzonitrile** (0.26 g; 90%) m.p 68-69 ^{0}C.. IR (NaCl): 2141 cm^{-1}. ^{1}H NMR (400 MHz, $CDCl_3$) δ 7.66 (d, J = 7.6 Hz, 1H), 7.62 (s, 1H), 7.57 (d, J = 8.2 Hz, 1H), 7.51 (t, J = 7.9 Hz, 1H). ^{13}C NMR (101 MHz, $CDCl_3$) δ 167.08, 132.45, 130.32, 129.37, 116.37, 113.56.

1.2.18 1-(isocyanomethyl)-4-methoxybenzene

To a solution of 4-methoxy benzyl formamide (0.33 g; 2 mmol) in triethylamine (2 ml) and subsequently phosphorus oxychloride (2 mmol) 0.2 ml were added at 0 °C. The reaction mixture was stirred for about 5 minutes. The progress of the reaction was monitored by TLC. After the completion of reaction, the reaction mixture was poured directly into a dry packed column. 100 % diethyl ether was used as the

mobile phase to afford a white solid **1-(isocyanomethyl)-4-methoxybenzene** (0.29 g; 88%) m.p 32-34 ^{0}C. IR (NaCl): 2153 cm^{-1}. ^{1}H NMR (400 MHz, $CDCl_3$) δ 7.14 (d, *J* = 8.8 Hz, 2H), 6.80 (d, *J* = 8.8 Hz, 2H), 4.42 (s, 2H), 3.68 (s, 3H). ^{13}C NMR (101 MHz, $CDCl_3$) δ 159.70, 156.92, 127.47, 124.00, 113.52, 54.73, 44.54.

1.2.19 1-isocyano-4-methoxy benzene

To a solution of *N*-(4-methoxyphenyl)formamide (0.30 g; 2 mmol) in triethylamine (2 ml) and subsequently phosphorus oxychloride (2 mmol) 0.2 ml were added at 0 °C. The reaction mixture was stirred for about 5 minutes. The progress of the reaction was monitored by TLC. After the completion of reaction, the reaction mixture was poured directly into a dry packed column. 100 % diethyl ether was used as the mobile phase to afford a yellow solid **1-isocyano-4-methoxy benzene** (0.27 g; 90%) m.p 33-34 ◦C. IR (NaCl): 2161 cm^{-1}. ^{1}H NMR (400 MHz, $CDCl_3$) δ 7.20 (d, *J* = 8.8 Hz, 1H), 6.77 (d, *J* = 8.9 Hz, 1H), 3.72 (s, 2H). ^{13}C NMR (101 MHz, $CDCl_3$) δ 162.90, 160.05, 127.91, 114.89, 55.89.

1.2.20 1-(isocyanomethyl)-2-methoxybenzene

To a solution of 2-methoxy benzyl formamide (0.33 g; 2 mmol) in triethylamine (2 ml) and subsequently phosphorus oxychloride (2 mmol) 0.2 ml were added at 0 °C. The reaction mixture was stirred for about 5 minutes. The progress of the reaction was monitored by TLC. After the completion of reaction, the reaction mixture was poured directly into a dry packed column. 100 % diethyl ether was used as the mobile phase to afford a dark green solid **1-(isocyanomethyl)-2-methoxybenzene** (0.25 g; 76%) m.p 110-112 ◦C. IR (NaCl): 2145 cm^{-1}. ^{1}H NMR (400 MHz, $CDCl_3$) δ 7.31 (d, *J* = 7.5 Hz, 1H), 7.26 – 7.17 (m, 1H), 6.90 (t, *J* = 7.5 Hz, 1H), 6.83 (t, *J* = 7.5 Hz, 1H), 6.78 (dd, *J* = 8.2, 3.4 Hz, 1H), 4.53 (d, *J* = 18.9 Hz, 3H), 3.74 (d, *J* = 12.1 Hz, 4H). ^{13}C NMR (101 MHz, $CDCl_3$) δ 157.26, 156.22, 130.69, 129.67, 127.53, 125.41, 120.91, 111.05, 110.00, 55.09, 41.76, 41.05.

1.2.21 1,2-diisocyanobenzene

To a solution of o-phenelenediformamide (0.32 g; 2 mmol) in triethylamine (2 ml) and subsequently phosphorus oxychloride (2 mmol) 0.2 ml were added at 0 °C. The reaction mixture was stirred for about 5 minutes. The progress of the reaction was monitored by TLC. After the completion of reaction, the reaction mixture was poured directly into a dry packed column. 100 % diethyl ether was used as the mobile phase to afford a brown liquid **1,2-diisocyanobenzene** (0.29 g; 91%). IR (NaCl): 2157 cm^{-1}. ^{1}H NMR (400 MHz, $CDCl_3$) δ 7.59 – 7.02 (m, 1H). ^{13}C NMR (101 MHz, $CDCl_3$) δ 171.93, 130.14, 127.65.

1.2.22 Ethyl-2 isocyanobenzoate

To a solution of Ethyl 2-formamidobenzoate (0.39 g; 2 mmol) in triethylamine (2 ml) and subsequently

phosphorus oxychloride (2 mmol) 0.2 ml were added at 0 °C. The reaction mixture was stirred for about 5 minutes. The progress of the reaction was monitored by TLC. After the completion of reaction, the reaction mixture was poured directly into a dry packed column. 100 % diethyl ether was used as the mobile phase to afford a white solid **Ethyl-2 isocyanobenzoate** (0.33 g; 85%). IR (NaCl): 2125 cm^{-1}. ^{1}H NMR (400 MHz, $CDCl_3$) δ 7.79 (d, *J* = 8.0 Hz, 1H), 7.38 (td, *J* = 7.7, 1.6 Hz, 1H), 7.35 – 7.20 (m, 2H), 4.24 (q, *J* = 7.1 Hz, 2H), 1.24 (t, *J* = 7.2 Hz, 3H). ^{13}C NMR (101 MHz, $CDCl_3$) δ 169.28, 163.22, 132.13, 131.07, 128.58, 126.54, 62.10, 13.46.

1.2.23 1-isocyanonaphthalene

To a solution of *N*-(1-naphthy)formamide (0.34 g; 2 mmol) in triethylamine (2 ml) and subsequently phosphorus oxychloride (2 mmol) 0.2 ml were added at 0 ◦C. The reaction mixture was stirred for about 5 minutes. The progress of the reaction was monitored by TLC. After the completion of reaction, the reaction mixture was poured directly into a dry packed column. 100 % diethyl ether was used as the mobile phase to afford a green liquid **1-isocyanonaphthalene** (0.32 g; 96%). IR (NaCl): 2129 cm^{-1}.^{1}H NMR (400 MHz, $CDCl_3$) δ 7.95 (d, *J* = 8.4 Hz, 1H), 7.65 (dd, *J* = 8.1, 5.1 Hz, 2H), 7.48 – 7.30 (m, 3H), 7.23 – 7.16 (m, 1H). ^{13}C NMR (101 MHz, $CDCl_3$) δ 167.36, 133.45, 129.66, 128.20, 127.85, 127.28, 124.77, 124.37, 122.69.

1.2.24 1,5-diisocyanonaphthalene

To a solution of 1,5-difformamido naphthalein (0.37 g; 2 mmol) in triethylamine (2 ml) and subsequently phosphorus oxychloride (2 mmol) 0.2 ml were added at 0 °C. The reaction mixture was stirred for about 5 minutes. The progress of the reaction was monitored by TLC. After the completion of reaction, the reaction mixture was poured directly into a dry packed column. 100 % diethyl ether was used as the mobile phase to afford a brown solid **1,5-diisocyanonaphthalene** (0.30 g; 81%) m.p 198-200 ^{0}C. IR (NaCl): 2125 cm^{-1}. ^{1}H NMR (400 MHz, $CDCl_3$) δ 8.29 (d, *J* = 8.3 Hz, 1H), 7.74 (d, *J* = 7.3 Hz, 1H), 7.68 (t, *J* = 7.9 Hz, 1H). ^{13}C NMR (101 MHz, $CDCl_3$) δ 173.01, 128.21, 127.15, 125.80, 125.05, 114.58.

1.2.25 2-Isocyano anthracene-9, 10 dione

To a solution of 2-Formamido anthraquinone (0.50 g; 2 mmol) in triethylamine (2 ml) and subsequently phosphorus oxychloride (2 mmol) 0.2 ml were added at 0 °C. The reaction mixture was stirred for about 5 minutes. The progress of the reaction was monitored by TLC. After the completion of reaction, the reaction mixture was poured directly into a dry packed column. 100 % diethyl ether was used as the mobile phase to afford an orange colour solid **2-Isocyano anthracene-9, 10 dione** (0.34 g; 68%) m.p 81-83 ^{0}C.. IR (NaCl): 2121 cm^{-1}. ^{1}H NMR (400 MHz, DMSO) δ 7.73 (s, 1H), 7.60 (dd, *J* = 65.1, 17.0

Hz, 3H),7.39 (s, 2H), 7.28 – 7.17 (m, 1H). ^{13}C NMR (101 MHz, DMSO) δ 182.71, 181.29, 168.33, 135.43, 133.69, 132.33, 129.15, 127.07, 124.57.

1.2.26 4-Isocyanobenzoic acid

To a solution of 4-formamido benzoic acid (0.33 g; 2 mmol) in triethylamine (2 ml) and subsequently phosphorus oxychloride (2 mmol) 0.2 ml were added at 0 °C. The reaction mixture was stirred for about 5 minutes. The progress of the reaction was monitored by TLC. After the completion of reaction, the reaction mixture was poured directly into a dry packed column. 100 % diethyl ether was used as the mobile phase to afford a white liquid **4-isocyanobenzoic acid** (0.21 g; 64%). 1H NMR (400 MHz, MeOD) δ 9.83 (d, J = 10.8 Hz, 1H), 9.26 (d, J = 8.7 Hz, 1H), 9.12 (d, J = 8.5 Hz, 1H), 9.04 (d, J = 2.0 Hz, 1H), 5.11 (s, 1H). 13C NMR (101 MHz, MeOD) δ 171.64, 166.45, 139.26, 133.29, 131.96, 128.85, 127.74.

1.2.27 4-isocyano phenol

To a solution of 4-formamido phenol (0.27 ml; 2 mmol) in triethylamine (2 ml) and subsequently phosphorus oxychloride (2 mmol) 0.2 ml were added at 0 °C. The reaction mixture was stirred for about 5 minutes. The progress of the reaction was monitored by TLC. After the completion of reaction, the reaction mixture was poured directly into a dry packed column. 100 % diethyl ether was used as the mobile phase to afford a brown liquid **4-isocyano phenol** (0.21 g; 78%). ^{1}H NMR (400 MHz, MeOD) δ 8.37 (s, 1H), 7.65 (d, J = 14.4 Hz, 2H), 7.31 (d, J = 11.9 Hz, 3H). ^{13}C NMR (101 MHz, MeOD) δ 164.80, 161.30, 150.11, 134.00, 124.21, 121.42.

1.2.28 (5-chloro-2-isocyanophenyl)(2-fluorophenyl)methanone

To a solution of N-[4-chloro-2-(2-fluorobenzoyl)phenyl]formamide (0.56 g; 2 mmol) in triethylamine (2 ml) and subsequently phosphorus oxychloride (2 mmol) 0.2 ml were added at 0 °C. The reaction mixture was stirred for about 5 minutes. The progress of the reaction was monitored by TLC. After the completion of reaction, the reaction mixture was poured directly into a dry packed column. 100 % diethyl ether was used as the mobile phase to afford a yellow solid (**5-chloro-2-isocyanophenyl)(2-fluorophenyl)methanone** (0.36 g; 65%) m.p 72-74 ^{0}C.. IR (NaCl): 2110 cm^{-1}. ^{1}H NMR (400 MHz, $CDCl_3$) δ 7.54 – 7.35 (m, 2H), 7.28 – 7.11 (m, 4H), 6.66 (d, J = 8.8 Hz, 1H). ^{13}C NMR (101 MHz, $CDCl_3$) δ 194.31, 160.05, 157.95, 149.55, 134.82, 133.05, 132.04, 129.63, 127.83, 123.98, 120.15, 118.44, 116.30.

1.2.29 Isocyano(pyridine-3-yl)methanone

To a solution of *N*-formylnicotinamide (0.29 g; 2 mmol) in triethylamine (2 ml) and subsequently phosphorus oxychloride (2 mmol) 0.2 ml were added at 0 °C. The reaction mixture was stirred for about

5 minutes. The progress of the reaction was monitored by TLC. After the completion of reaction, the reaction mixture was poured directly into a dry packed column. 100 % diethyl ether was used as the mobile phase to afford a colourless liquid **Isocyano(pyridine-3-yl)methanone** (0.13 g; 45%). ^{1}H NMR (400 MHz, $CDCl_3$) δ 8.82 (s, 1H), 8.75 (dd, *J* = 5.0, 1.3 Hz, 1H), 7.93 (d, *J* = 8.0 Hz, 1H), 7.40 (dd, *J* = 8.0, 5.0 Hz, 1H). ^{13}C NMR (101 MHz, $CDCl_3$) δ 152.47, 151.88, 138.85, 123.20, 116.02, 109.54.

1.2.30 2-Isocyano Thiazole

To a solution of *N*-(thiazol-2-yl)formamide (0.26 g; 2 mmol) in triethylamine (2 ml) and subsequently phosphorus oxychloride (2 mmol) 0.2 ml were added at 0 °C. The reaction mixture was stirred for about 5 minutes. The progress of the reaction was monitored by TLC. After the completion of reaction, the reaction mixture was poured directly into a dry packed column. 100 % diethyl ether was used as the mobile phase to afford a brown liquid **2-isocyano thiazole** (0.14 g; 54%). ^{1}H NMR (400 MHz, $CDCl_3$) δ 8.66 (s, 1H), 7.49 (d, *J* = 3.7 Hz, 1H), 7.07 (d, *J* = 3.7 Hz, 1H). ^{13}C NMR (101 MHz, $CDCl_3$) δ 158.26, 134.81, 113.88.

1.2.31 4-isocyano-antipyrine

To a solution of 4-formamido antipyrine (0.5 g; 2 mmol) in triethylamine (2 ml) and subsequently phosphorus oxychloride (2 mmol) 0.2 ml were added at 0 °C. The reaction mixture was stirred for about 5 minutes. The progress of the reaction was monitored by TLC. After the completion of reaction, the reaction mixture was poured directly into a dry packed column. 100 % diethyl ether was used as the mobile phase to afford a yellow solid **4-isocyano-antipyrine** (0.34 g; 68%). IR (NaCl): 2131 cm^{-1}. ^{1}H NMR (400 MHz, $CDCl_3$) δ 7.50 (d, *J* = 1.8 Hz, 1H), 7.48 (s, 2H), 7.46 (t, *J* = 1.9 Hz, 2H), 7.39 (t, *J* = 1.2 Hz, 1H), 7.38 (t, *J* = 1.9 Hz, 1H), 7.36 (t, *J* = 1.2 Hz, 1H), 7.32 (d, *J* = 2.1 Hz, 3H), 7.30 (t, *J* = 1.7 Hz, 2H), 3.18 (s, 3H). ^{13}C NMR (101 MHz, $CDCl_3$) δ 169.86, 159.00, 150.64, 133.78, 129.27, 128.22, 125.44, 35.13, 10.67.

1.2.32 1,2,4-trichloro-5-isocyanobenzene

To a solution of *N*-(2,4,5-trichlorophenyl)formamide (0.45 g; 2mmol) in triethylamine (2 ml) and subsequently phosphorus oxychloride (2 mmol) 0.2 ml were added at 0 °C. The reaction mixture was stirred for about 5 minutes. The progress of the reaction was monitored by TLC. After the completion of reaction, the reaction mixture was poured directly into a dry packed column. 100 % diethyl ether was used as the mobile phase to afford a brown liquid **1,2,4-trichloro-5-isocyanobenzene** (0.39 g; 87%). IR (NaCl): 2134 cm^{-1}. 1H NMR (400 MHz, CDCl3) δ 7.46 (d, J = 18.6 Hz, 1H), 5.21 (s, 1H). ^{13}C NMR (101 MHz, $CDCl_3$) δ 172.07, 134.61, 131.94, 131.21, 129.79, 128.90.

1.2.33 2-isocyanobenzenethiol

To a solution of *N*-(2-mercaptophenyl)formamide (0.31 ml; 2mmol) in triethylamine (2 ml) and subsequently phosphorus oxychloride (2 mmol) 0.2 ml were added at 0 °C. The reaction mixture was stirred for about 5 minutes. The progress of the reaction was monitored by TLC. After the completion of reaction, the reaction mixture was poured directly into a dry packed column. 100 % diethyl ether was used as the mobile phase to afford a yellow liquid **2-isocyanobenzenethiol** (0.23 ml; 76%). IR (NaCl): 2127 cm^{-1}. 1H NMR (400 MHz, CDCl3) δ 8.80 (s, 1H), 7.99 (d, J = 8.2 Hz, 1H), 7.74 (d, J = 8.1 Hz, 1H), 7.32 (t, J = 7.7 Hz, 1H), 7.23 (t, J = 7.6 Hz, 1H). 13C NMR (101 MHz, CDCl3) δ 154.03, 153.20, 133.70, 126.16, 125.53, 123.59, 121.89.

1.2.34 2-isocyano-1-methoxy-4-methylbenzene

To a solution of *N*-(2-methoxy-5-methylphenyl)formamide (0.33 g; 2mmol) in triethylamine (2 ml) and subsequently phosphorus oxychloride (2 mmol) 0.2 ml were added at 0 °C. The reaction mixture was stirred for about 5 minutes. The progress of the reaction was monitored by TLC. After the completion of reaction, the reaction mixture was poured directly into a dry packed column. 100 % diethyl ether was used as the mobile phase to afford a colourless liquid **2-isocyano-1-methoxy-4-methylbenzene** (0.31 ml; 93%). IR (NaCl): 2136 cm^{-1}. 1H NMR (400 MHz, CDCl3) δ 7.00 (d, J = 8.6 Hz, 1H), 6.95 (s, 1H), 6.71 (d, J = 8.6 Hz, 1H), 3.74 (s, 3H), 2.13 (s, 3H). 13C NMR (101 MHz, CDCl3) δ 166.75, 152.79, 130.78, 130.00, 127.60, 111.48, 56.21, 20.06.

1.3. General procedure for passerini product

To a solution of 2-formamido-benzothiazole (2 mmol) in triethylamine (5 mmol) and subsequently phosphorus oxychloride (2 mmol) 0.2 ml were added at 0 °C. The reaction mixture was stirred for 5 min. The progress of the reaction was monitored by TLC. After the completion of reaction, DCM (2 ml), benzoic acid (2 mmol), benzaldehyde (2 mmol) were added. The reaction mixture was stirred at room temperature for 24 hours. Upon the completion of reaction as indicated by TLC the resultant mixture was extracted with DCM. The combined organic solution was washed with water and brine, dried with anhydrous Na_2SO_4, and concentrated under reduced pressure. The column chromatographic purification of crude mass on silica gel eluting with DCM) afforded MCR products in good yield.

NMR spectral data of synthesised compounds

2.1 1-bromo-3-isocyanobenzene

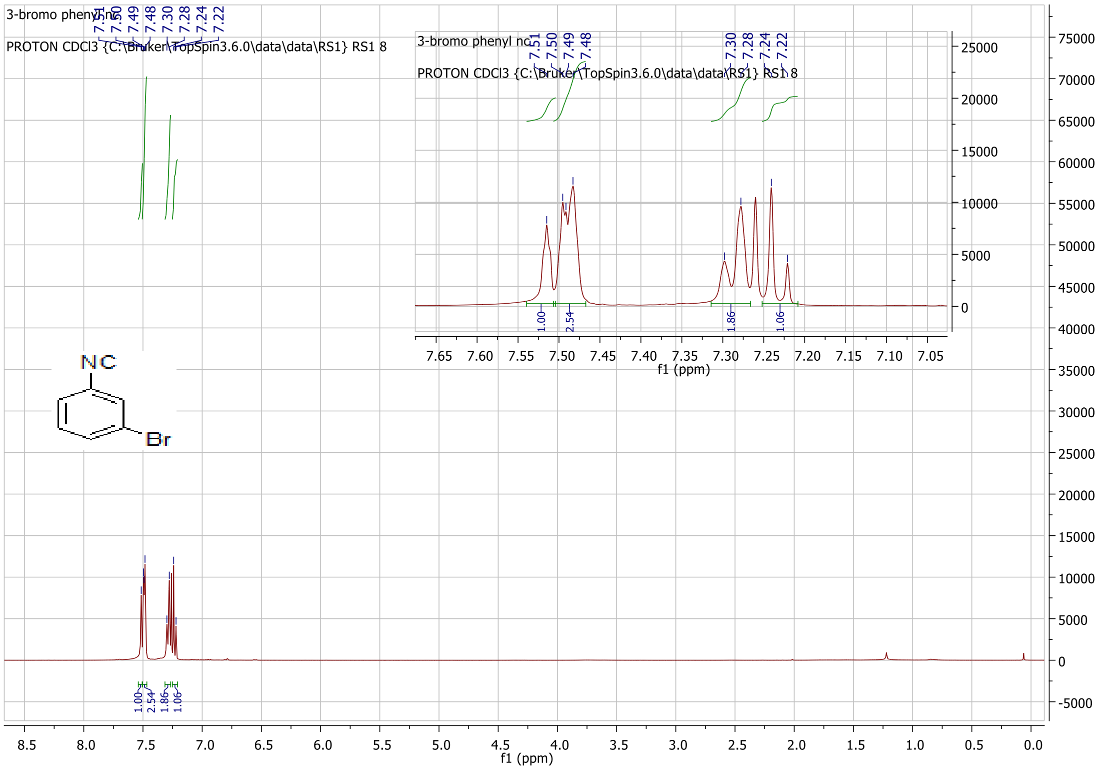

Figure S2. ^{1}H NMR spectrum (400 MHz, $CDCl_3$)

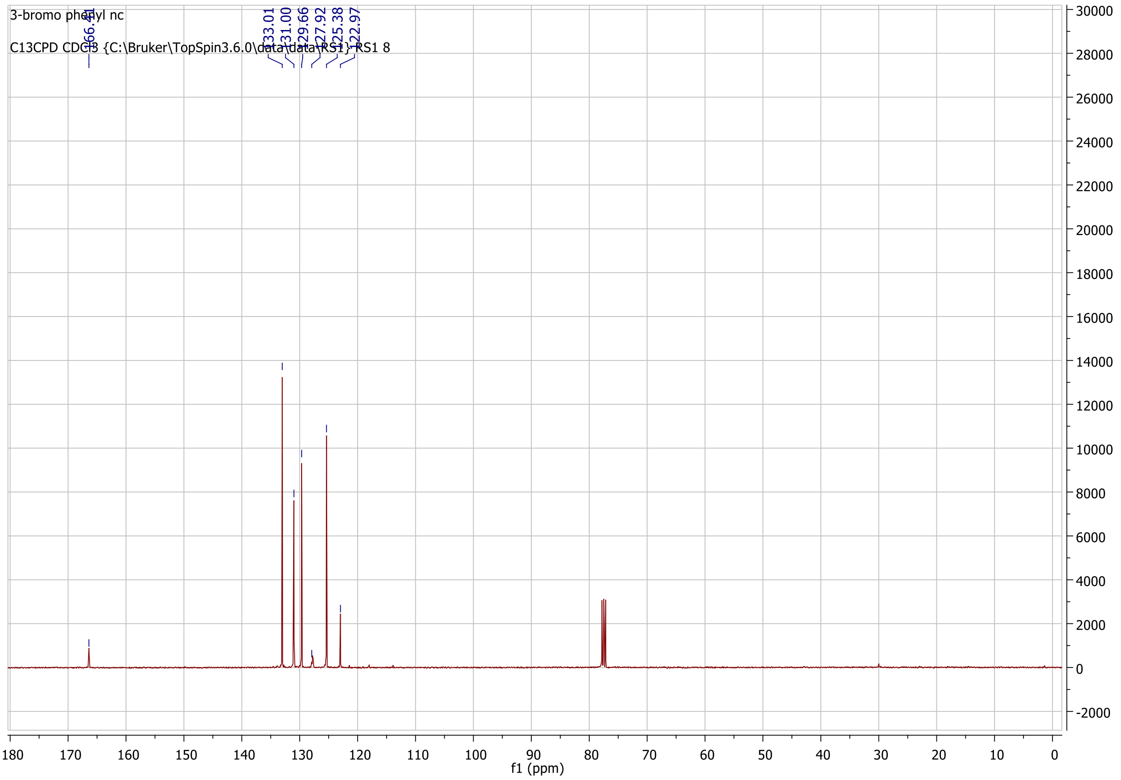

Figure S3. ^{13}C NMR spectrum (101 MHz, $CDCl_3$)

2.2 1-bromo-4-isocyanobenzene

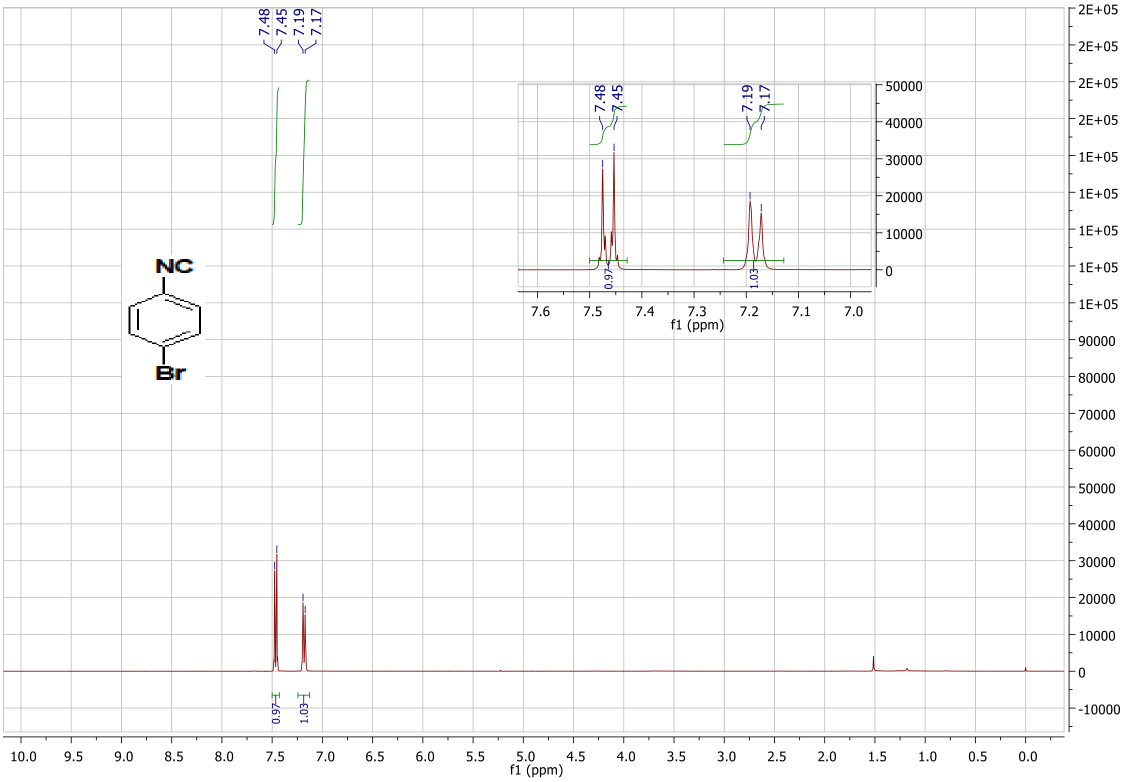

Figure S4. ^{1}H NMR spectrum (400 MHz, $CDCl_3$)

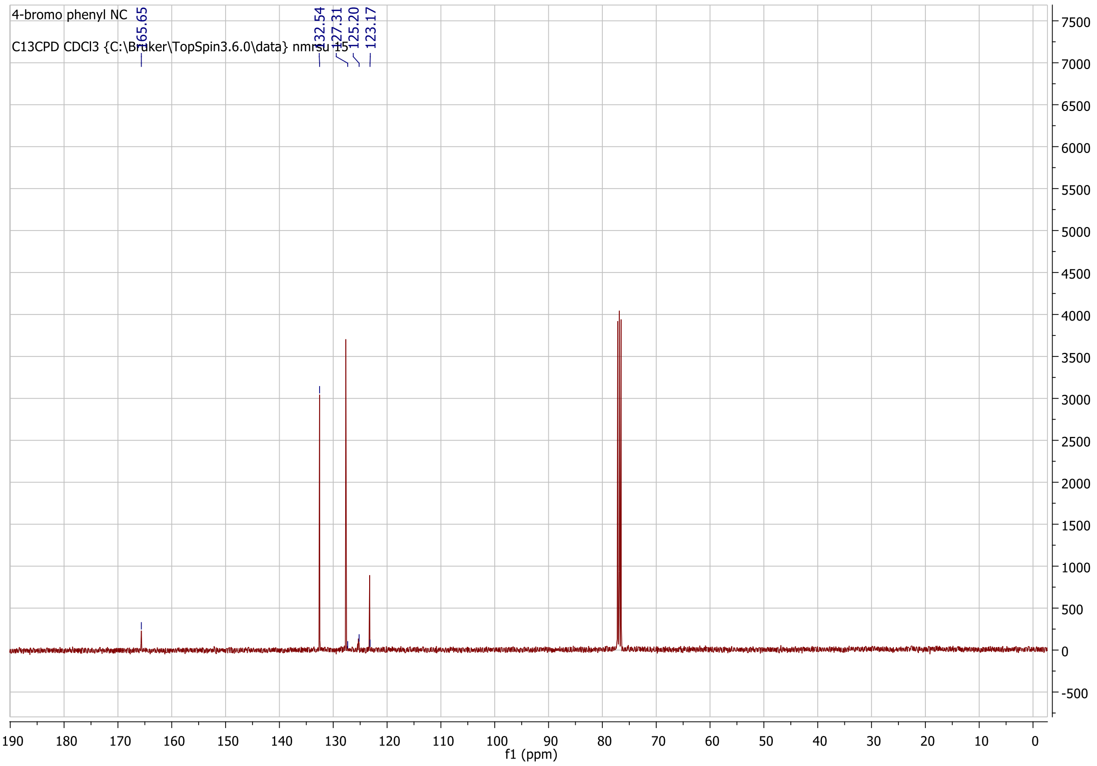

Figure S5. ^{13}C NMR spectrum (101 MHz, $CDCl_3$)

2.3. 1-chloro-2-isocyanobenzene

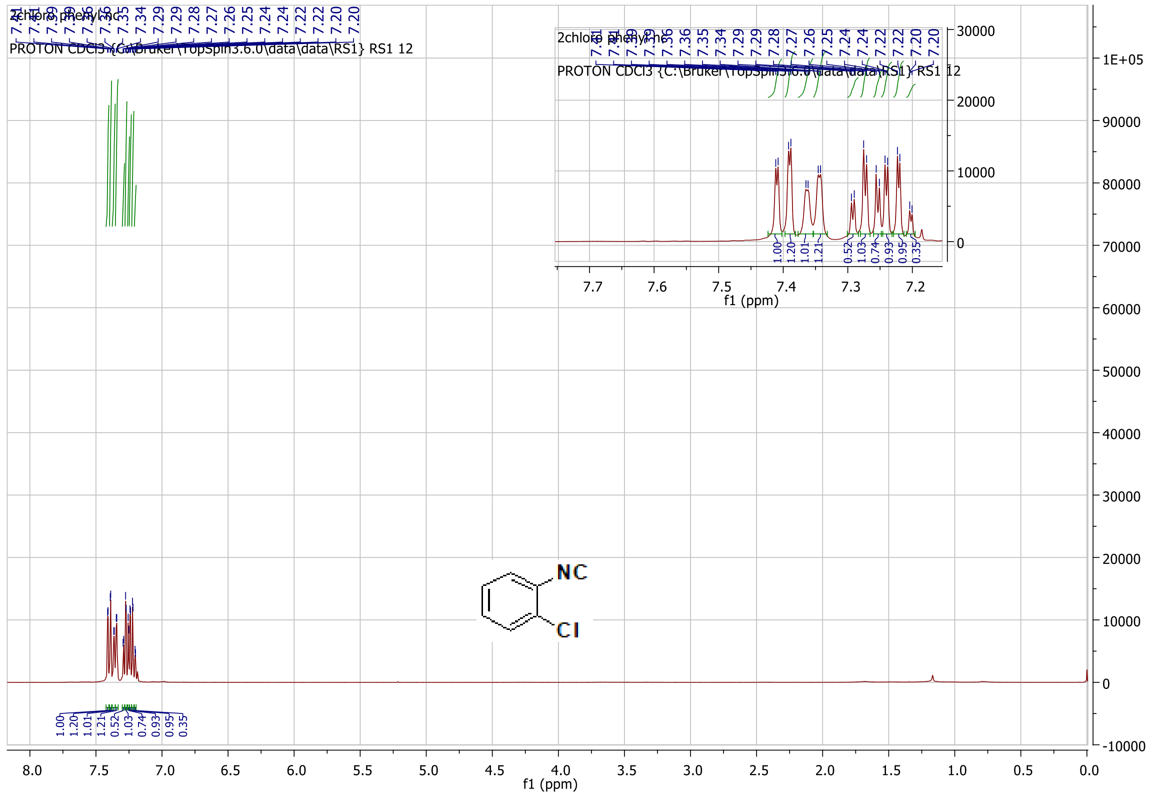

Figure S6. ^{1}H NMR spectrum (400 MHz, $CDCl_3$)

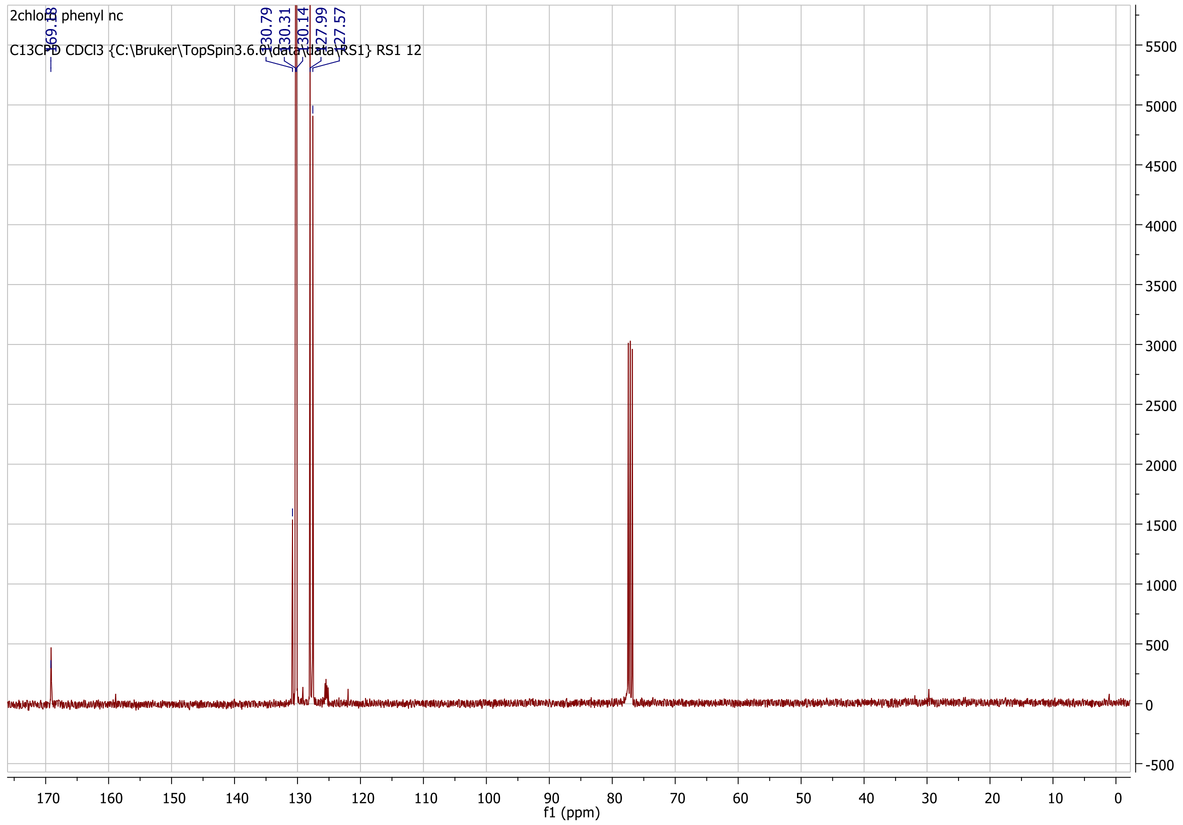

Figure S7. ^{13}C NMR spectrum (101 MHz, $CDCl_3$)

2.4. 1-bromo-2-isocyanobenzene

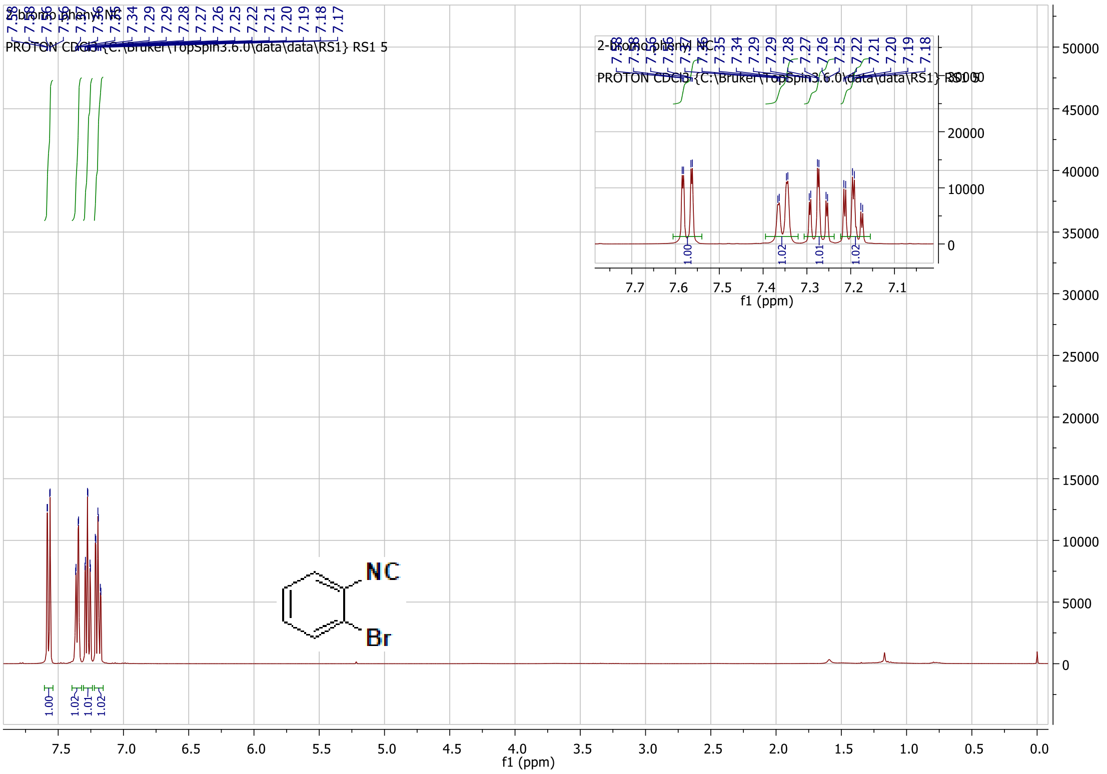

Figure S8. ^{1}H NMR spectrum (400 MHz, $CDCl_3$)

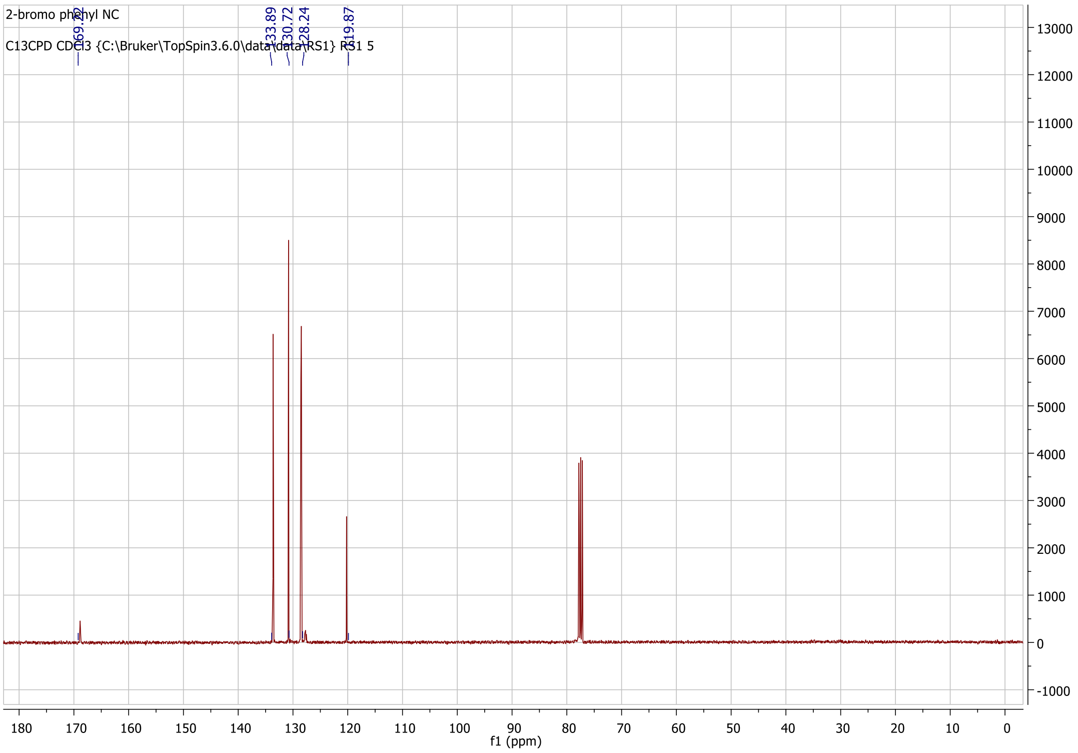

Figure S9. ^{13}C NMR spectrum (101 MHz, $CDCl_3$)

2.5. 1-iodo-2-isocyanobenzene

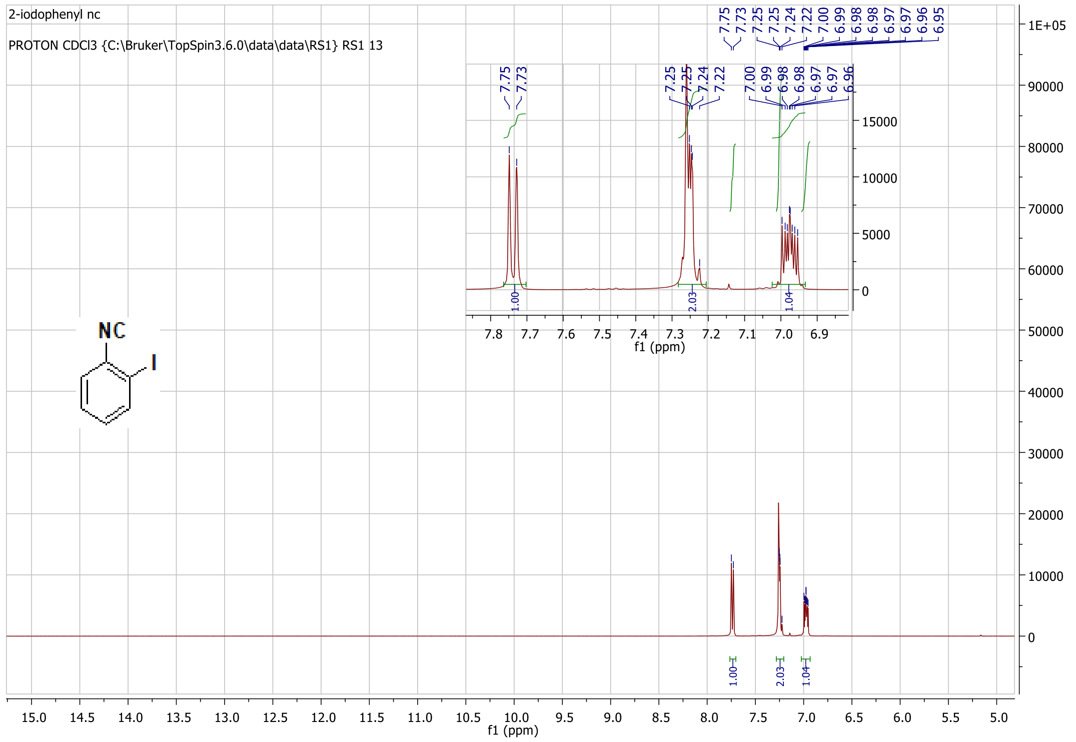

Figure S10. ^{1}H NMR spectrum (400 MHz, $CDCl_3$)

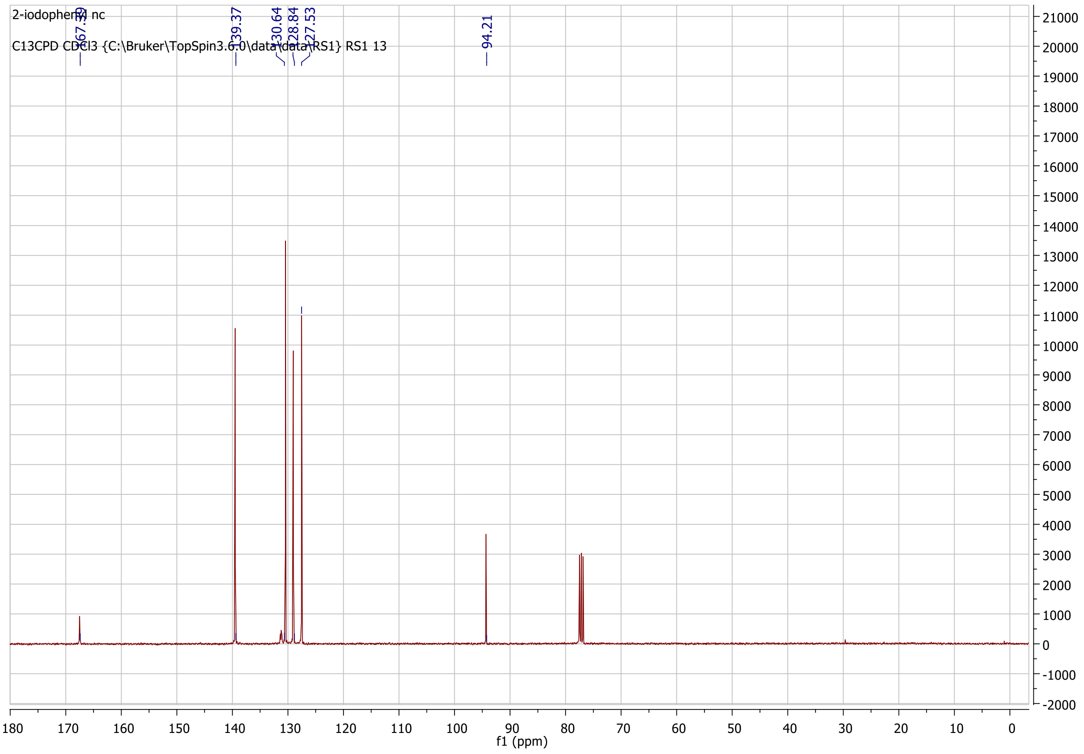

Figure S11. ^{13}C NMR spectrum (101 MHz, $CDCl_3$)

2.6. 1-fluoro-3-isocyanobenzene

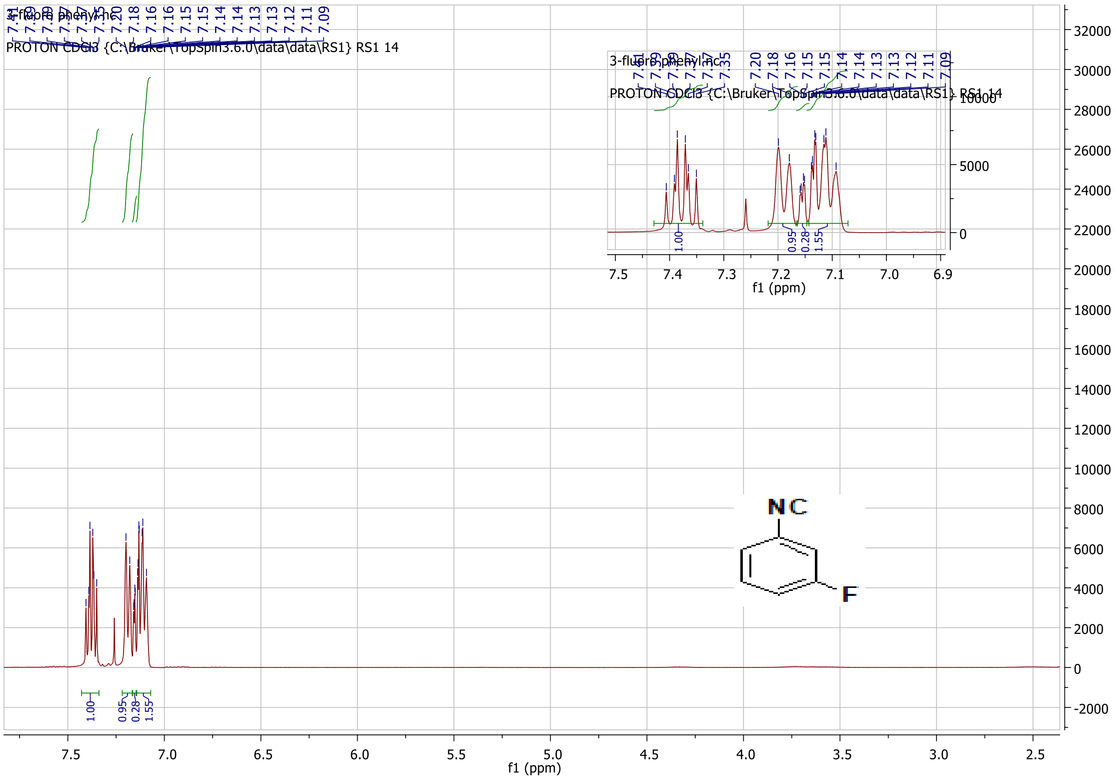

Figure S12. ^{1}H NMR spectrum (400 MHz, $CDCl_3$)

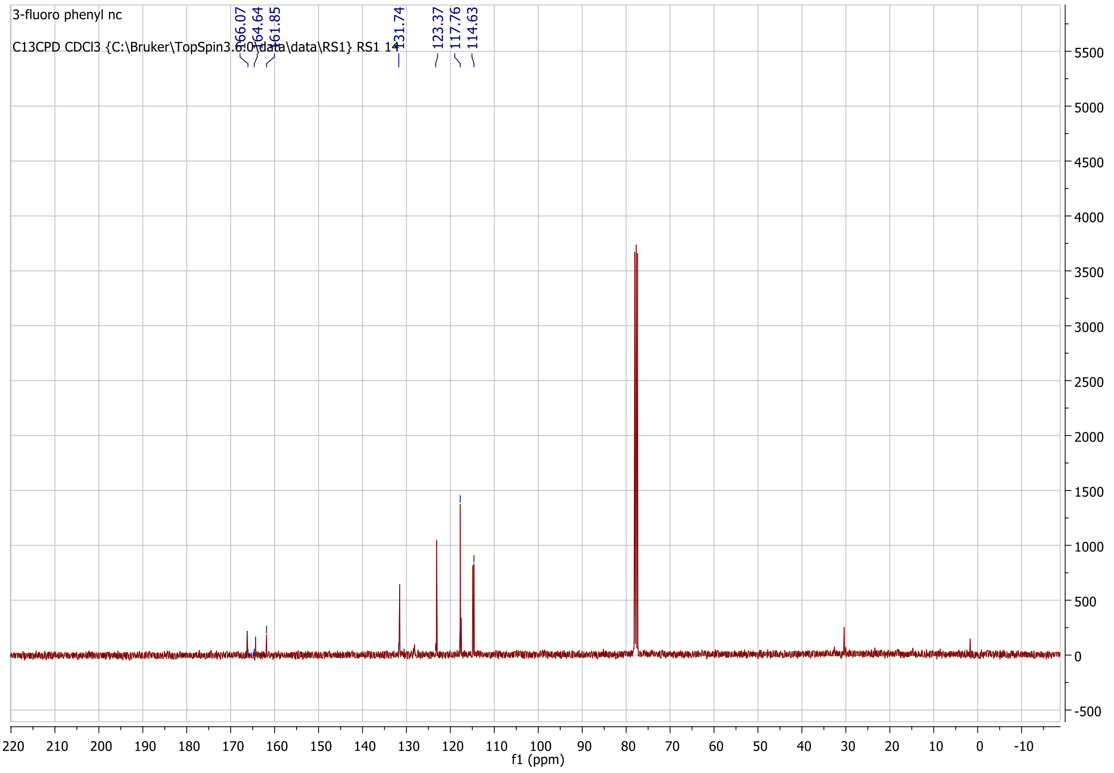

Figure S13. ^{13}C NMR spectrum (101 MHz, $CDCl_3$)

2.7. 1,2-difluoro-4-isocyanobenzene

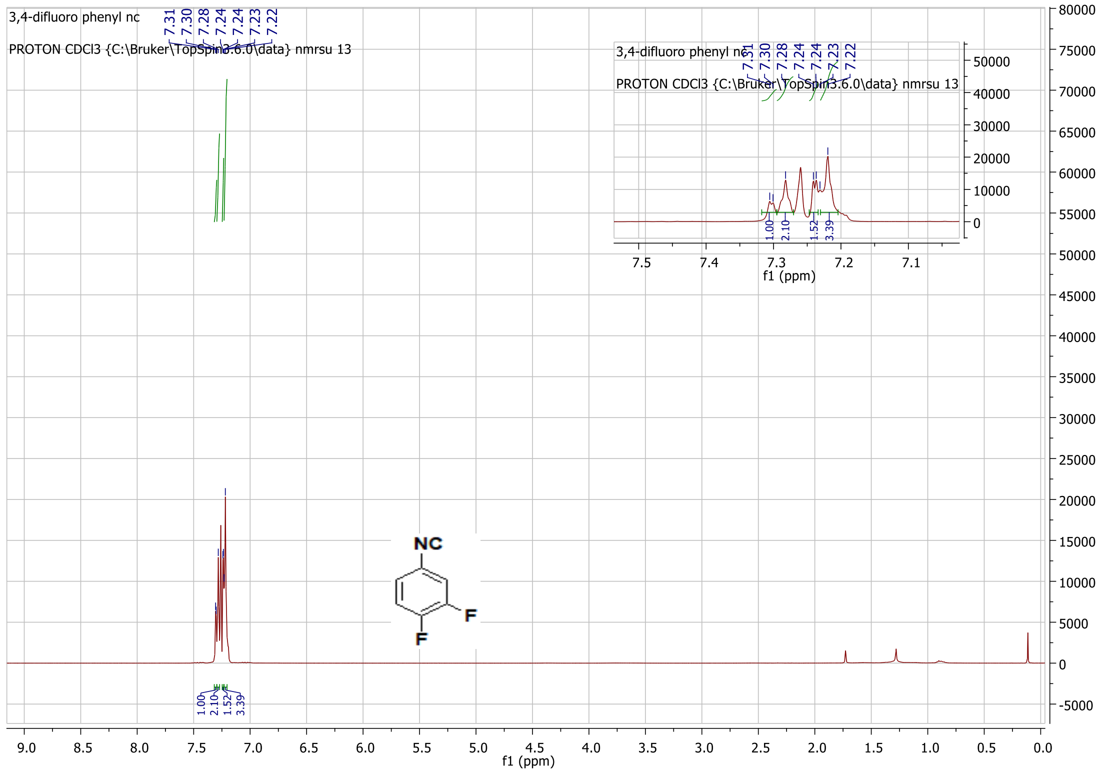

Figure S14. ^{1}H NMR spectrum (400 MHz, $CDCl_3$)

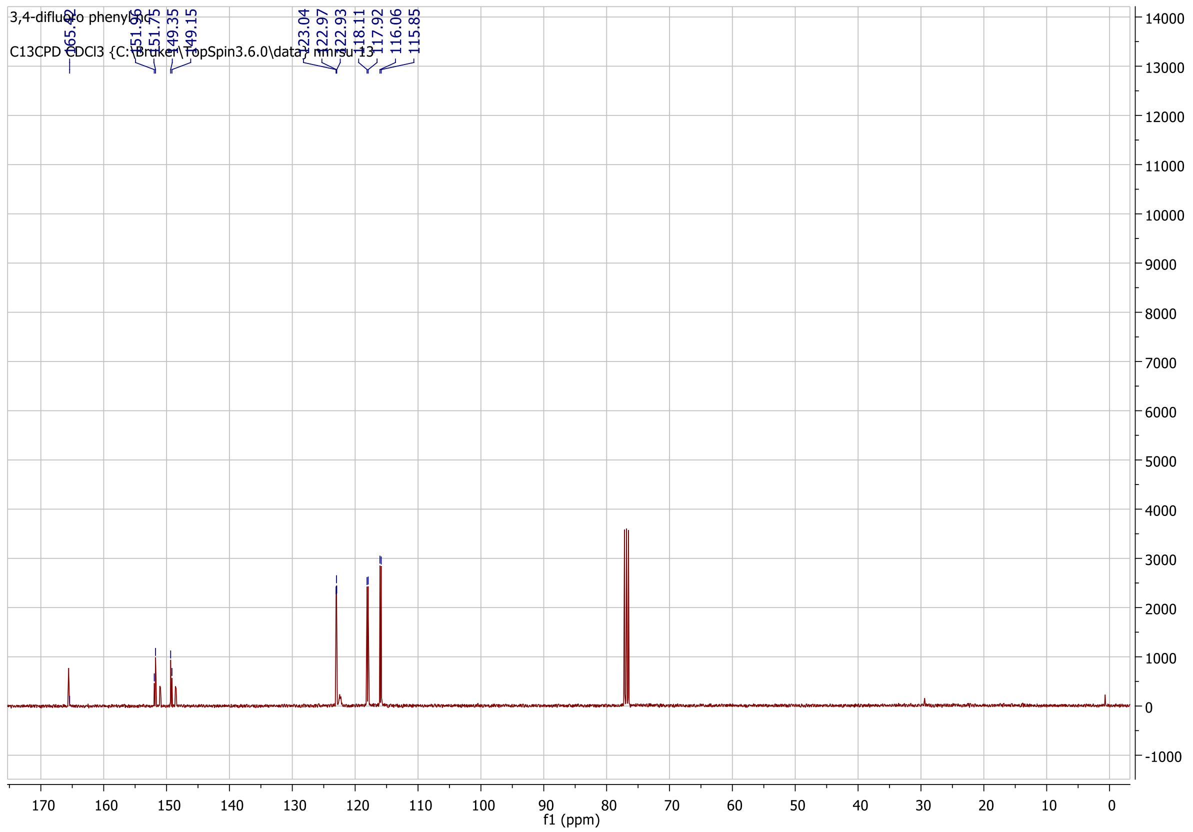

Figure S15. ^{13}C NMR spectrum (101 MHz, $CDCl_3$)

2.8. 1,2-dichloro-4-isocyanobenzene

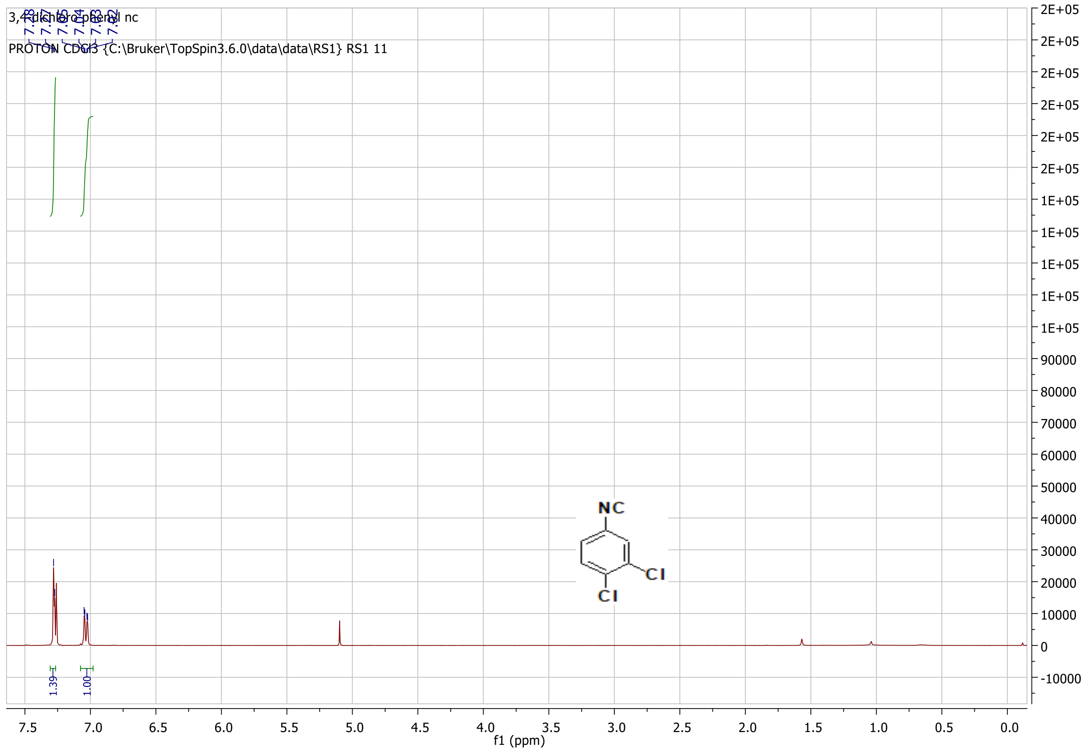

Figure S16. ^{1}H NMR spectrum (400 MHz, $CDCl_3$)

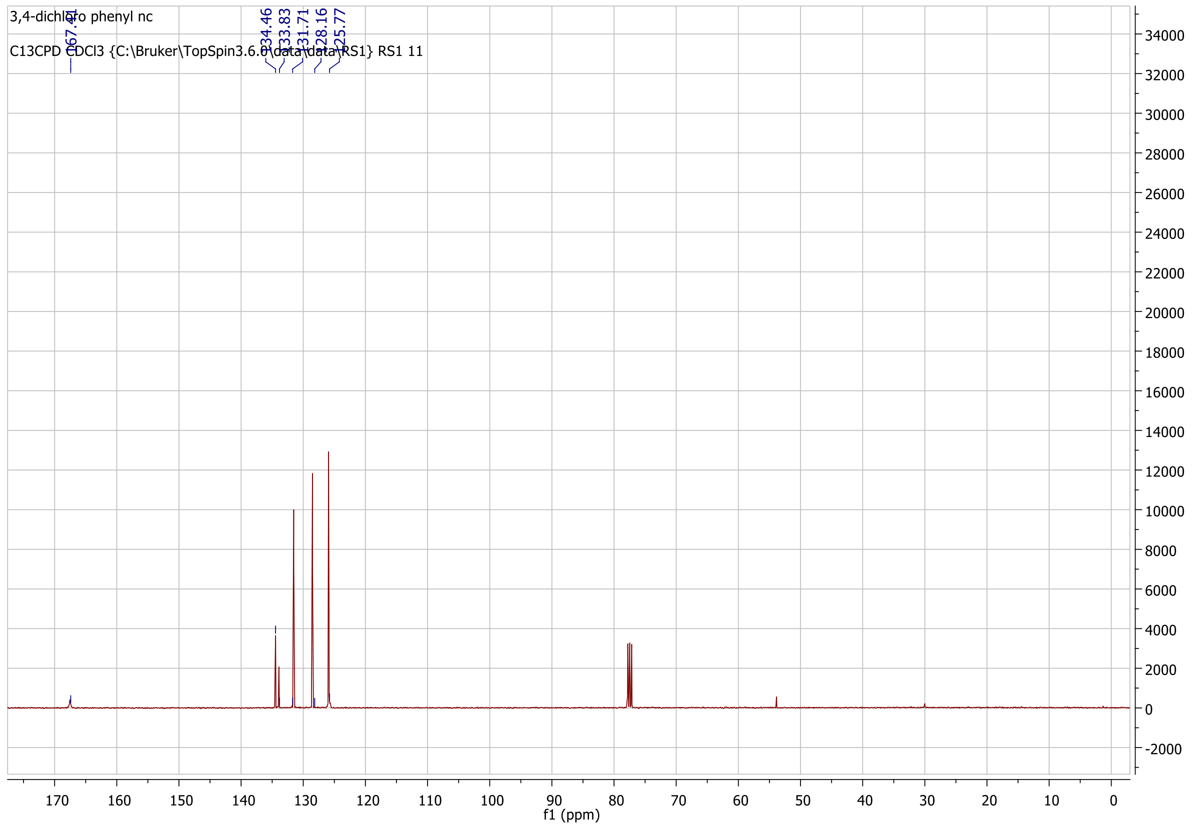

Figure S17. ^{13}C NMR spectrum (101 MHz, $CDCl_3$)

2.9. 1-Isocyano-2-nitrobenzene

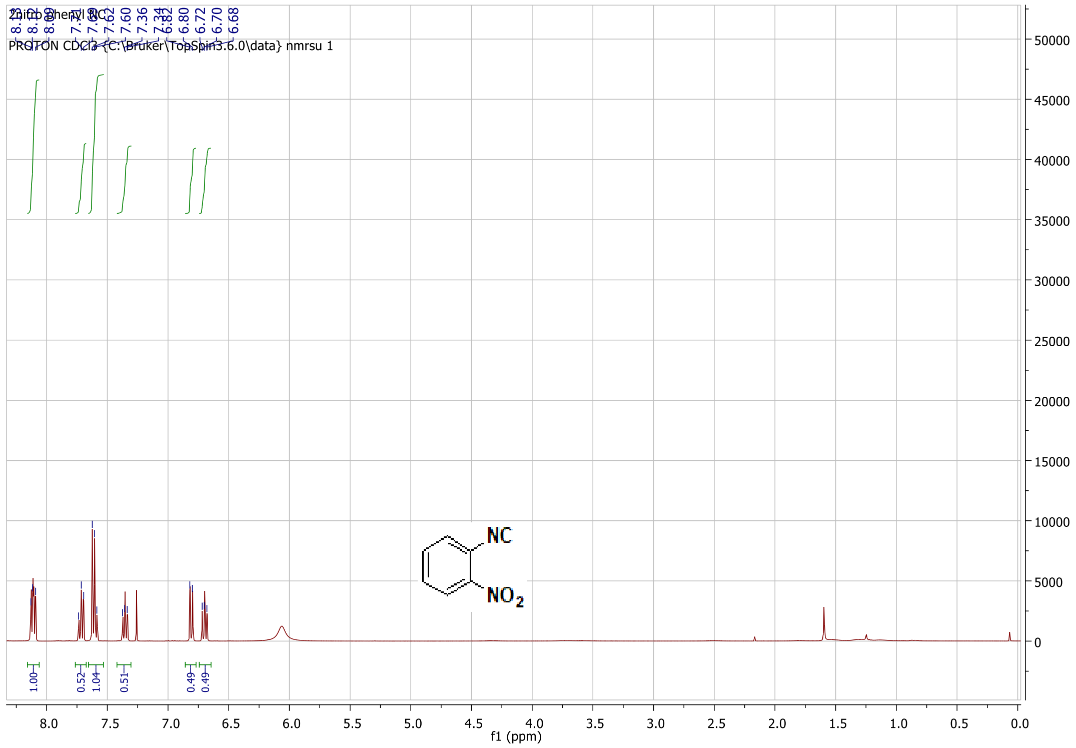

Figure S18. ^{1}H NMR spectrum (400 MHz, $CDCl_3$)

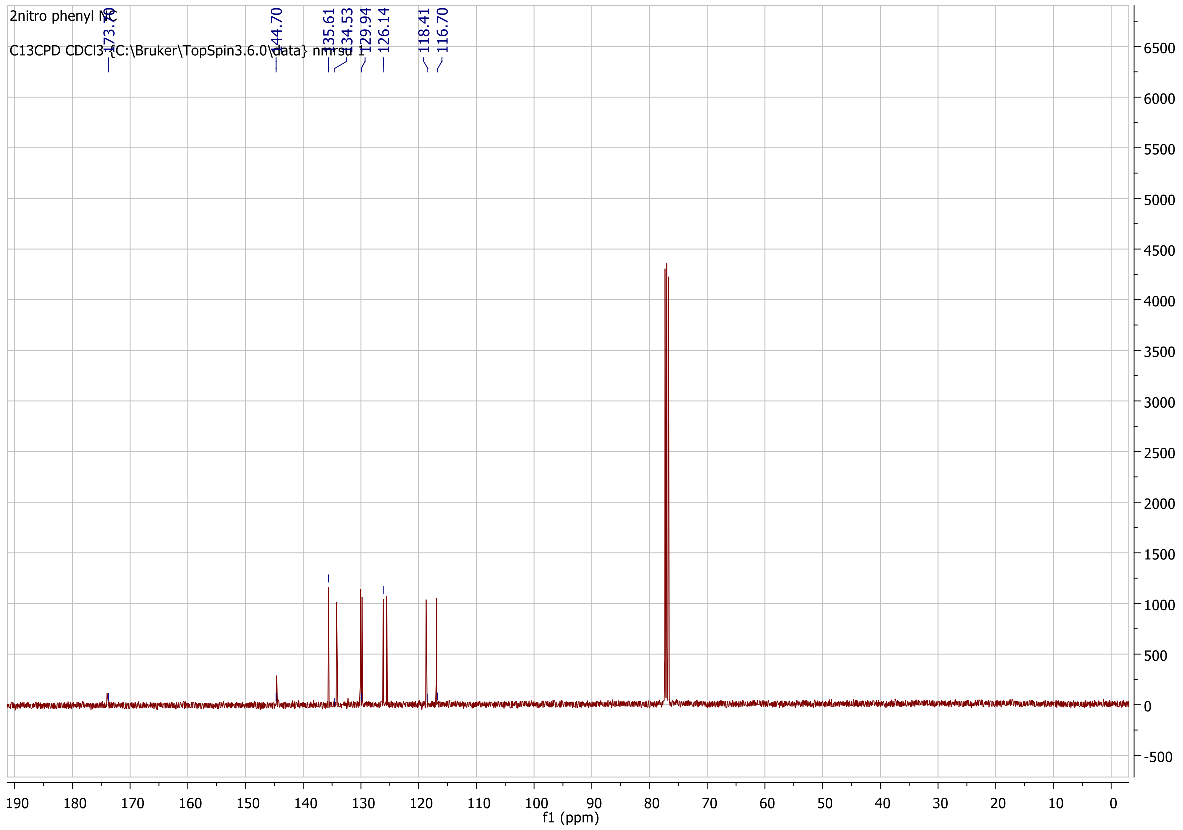

Figure S19. ^{13}C NMR spectrum (101 MHz, $CDCl_3$)

2.10. 4-Nitro phenyl isocyanide

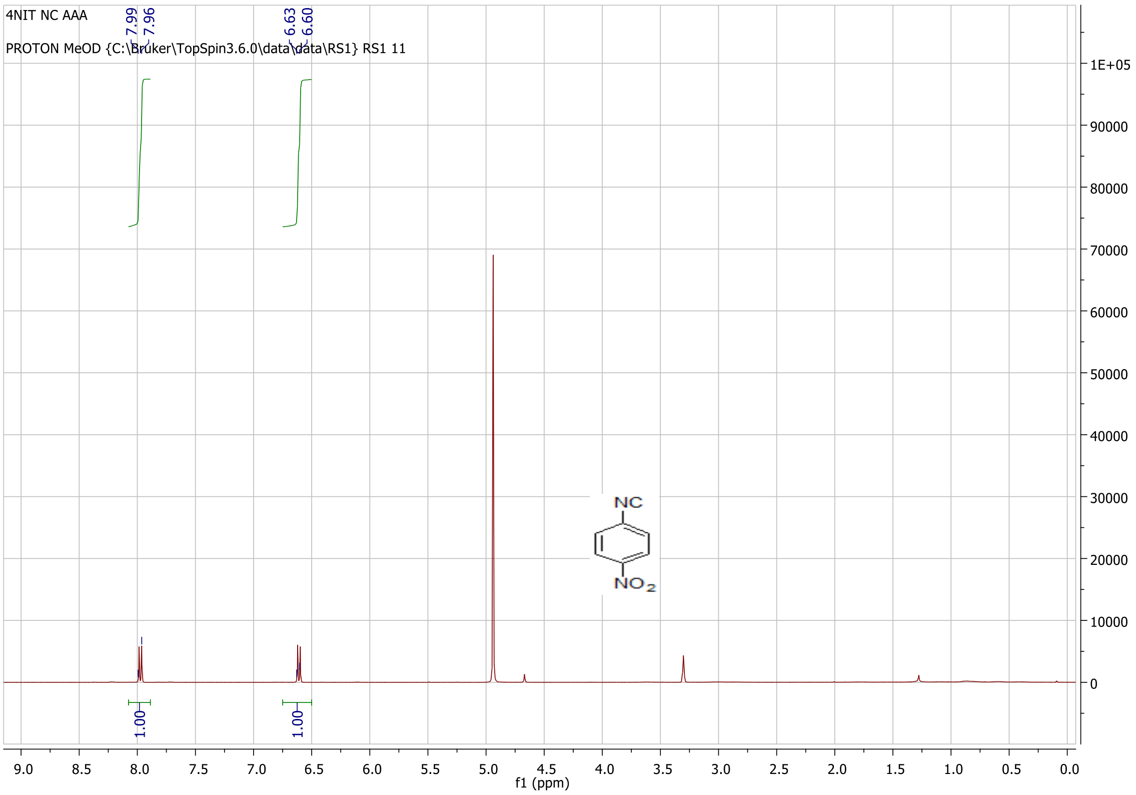

Figure S20. ^{1}H NMR spectrum (400 MHz, MeOD)

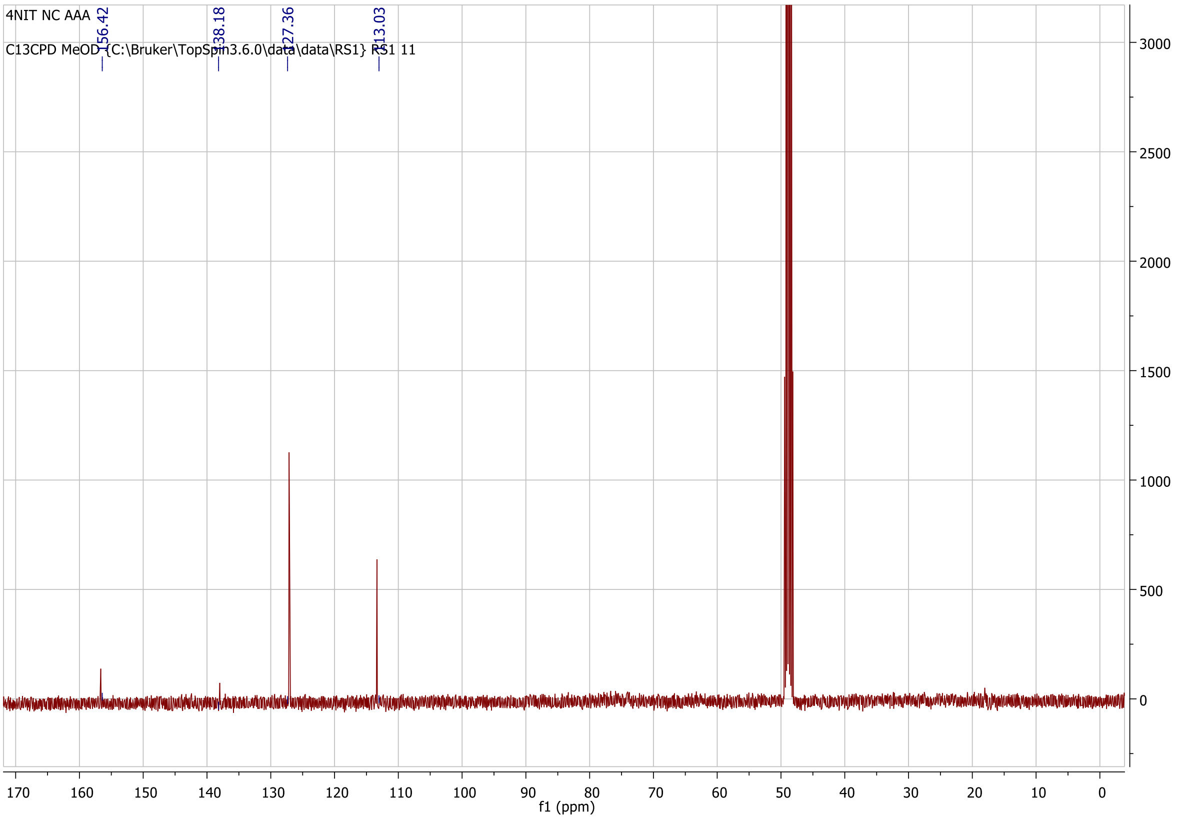

Figure S21. ^{13}C NMR spectrum (101 MHz, MeOD)

2.11. 4-methyl-2-nitro phenyl isocyanide

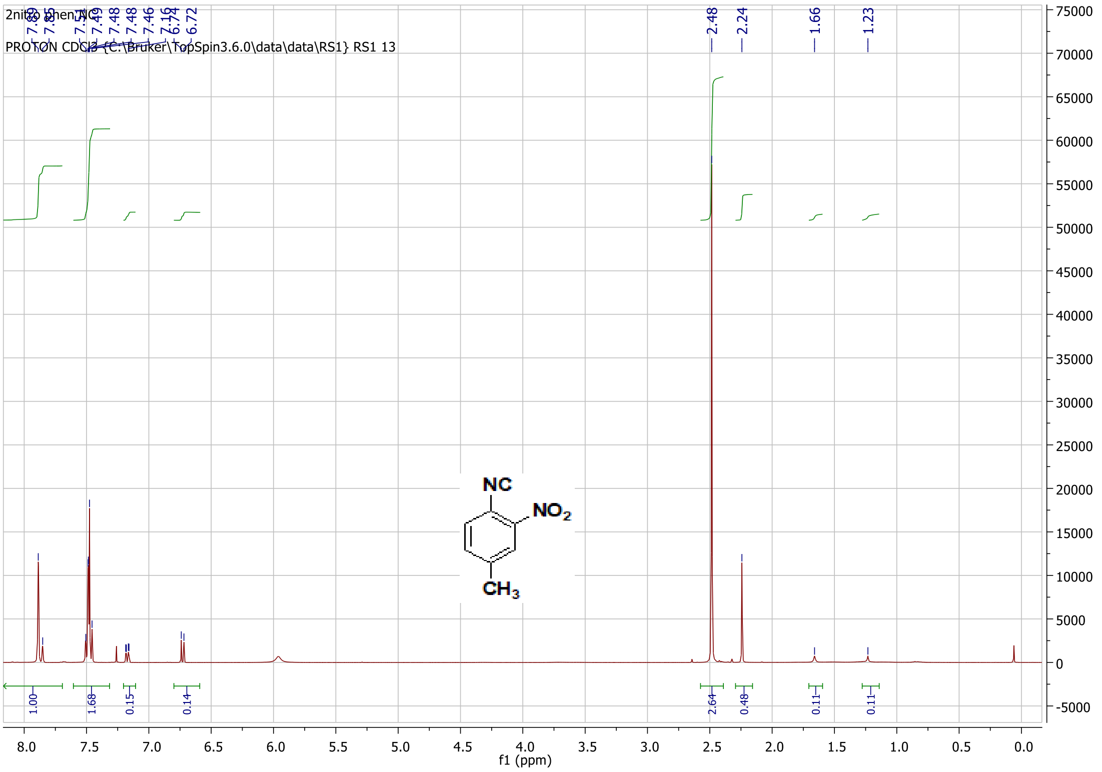

Figure S22. ^{1}H NMR spectrum (400 MHz, $CDCl_3$)

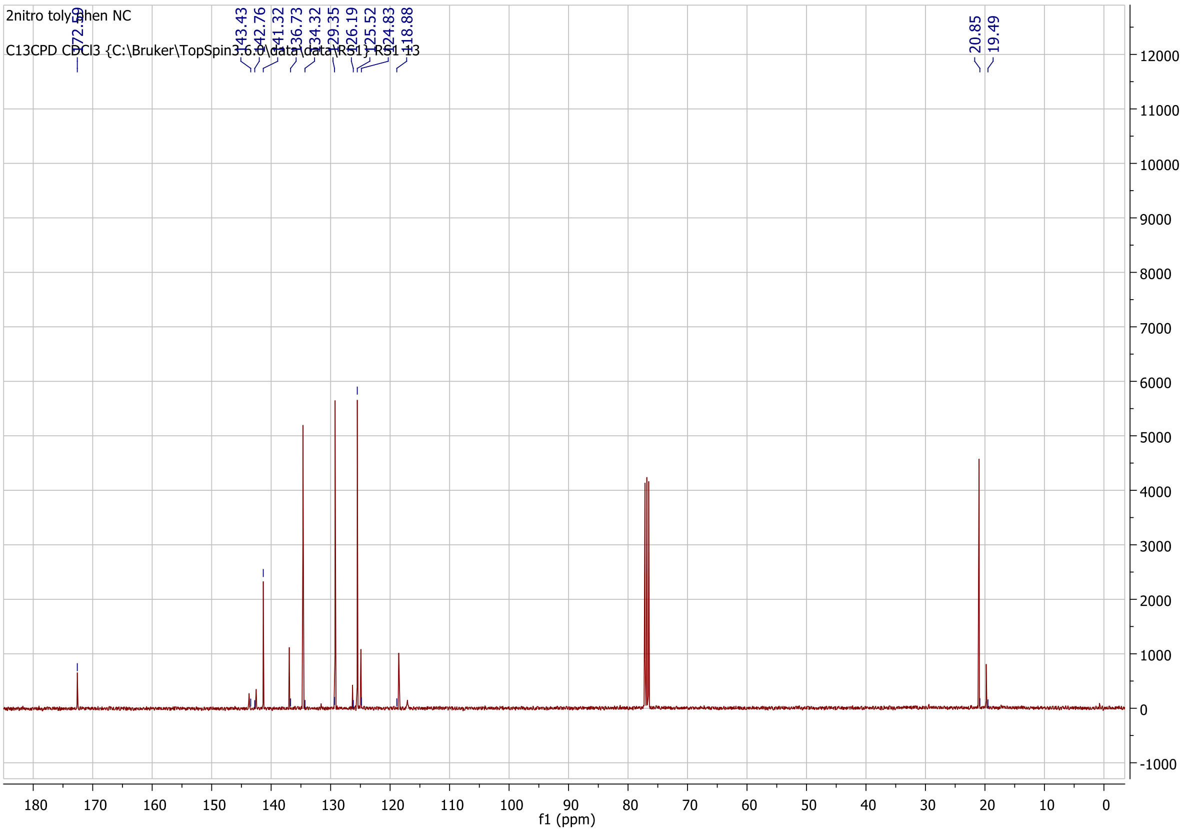

Figure S23. ^{13}C NMR spectrum (101 MHz, $CDCl_3$)

2.12 2-isocyano-4-nitrophenol

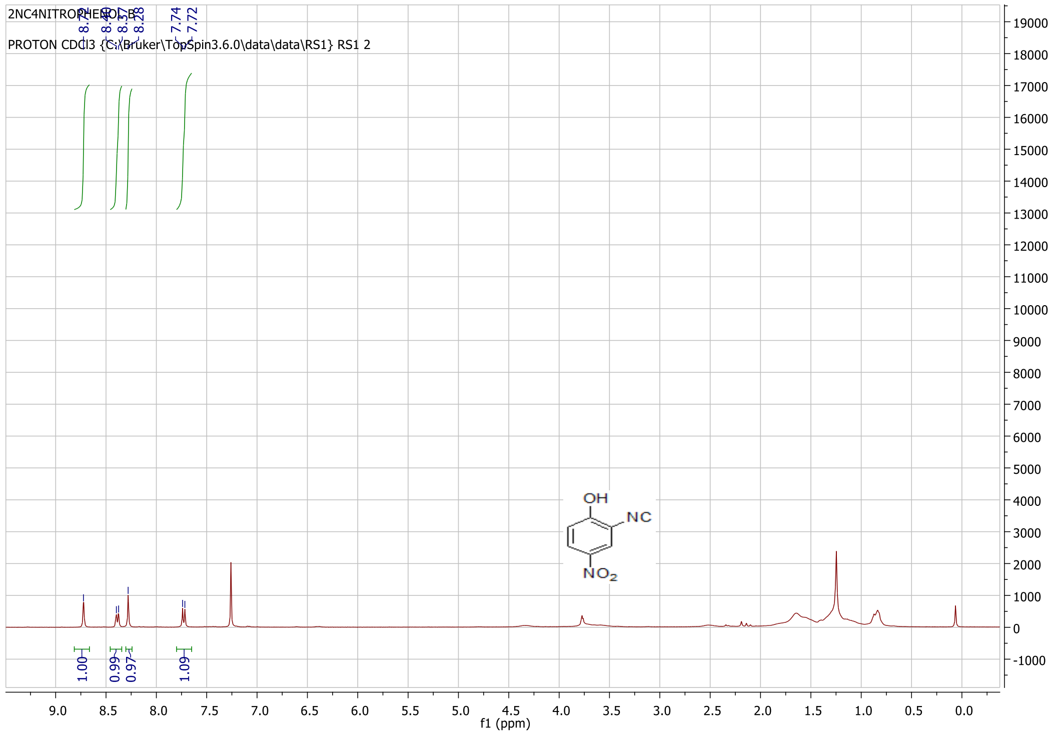

Figure S24. ^{1}H NMR spectrum (400 MHz, $CDCl_3$)

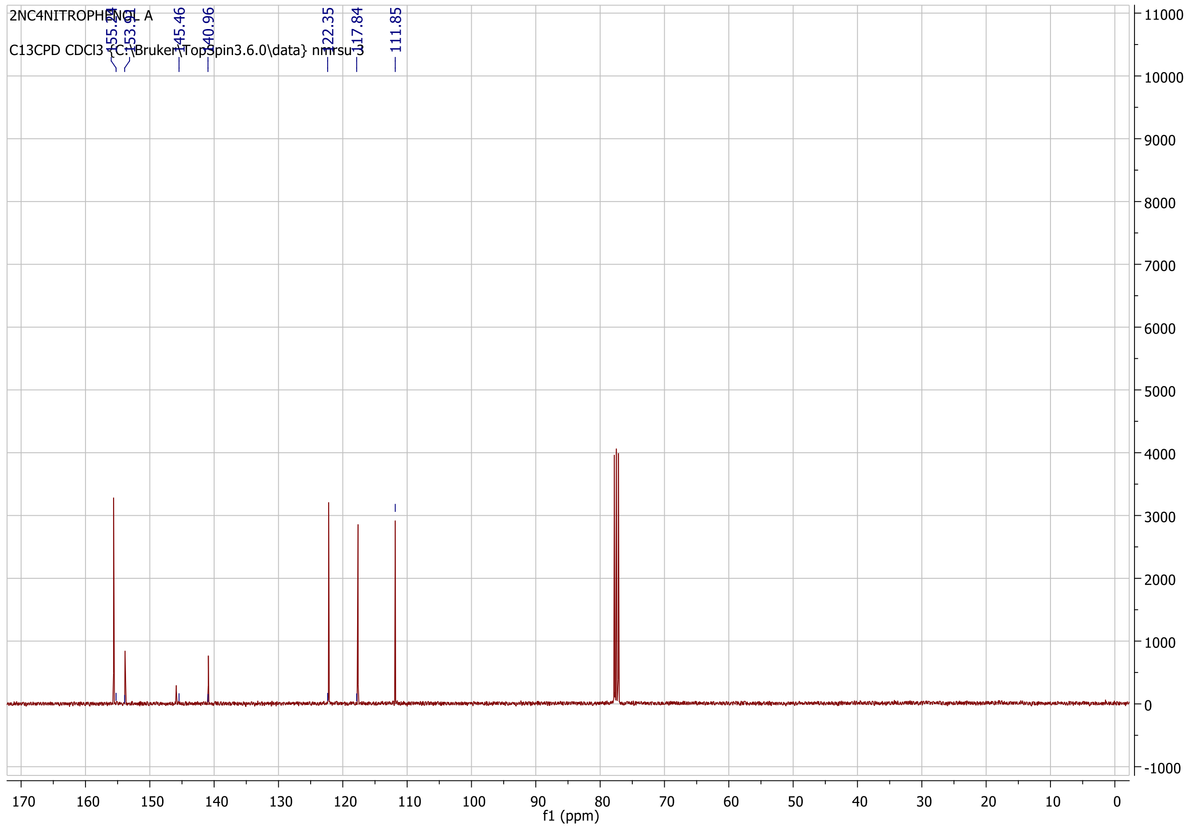

Figure S25. ^{13}C NMR spectrum (101 MHz, $CDCl_3$)

2.13. 1-isocyano-4-methylbenzene

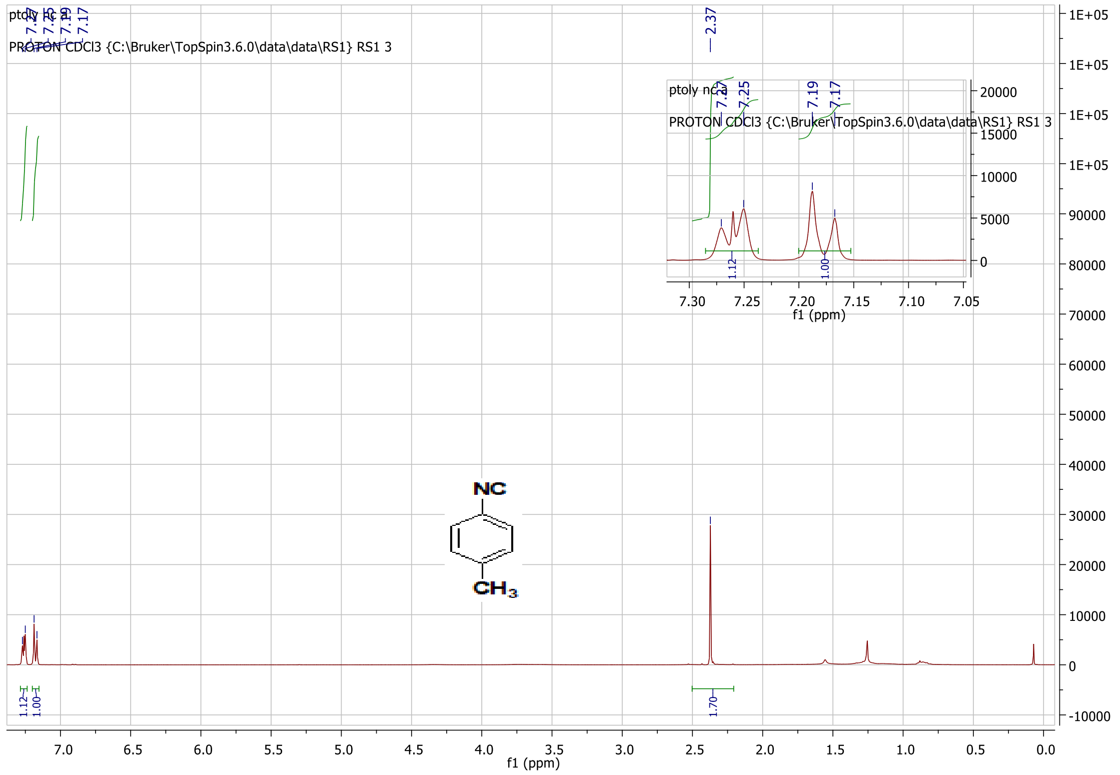

Figure S26. ^{1}H NMR spectrum (400 MHz, $CDCl_3$)

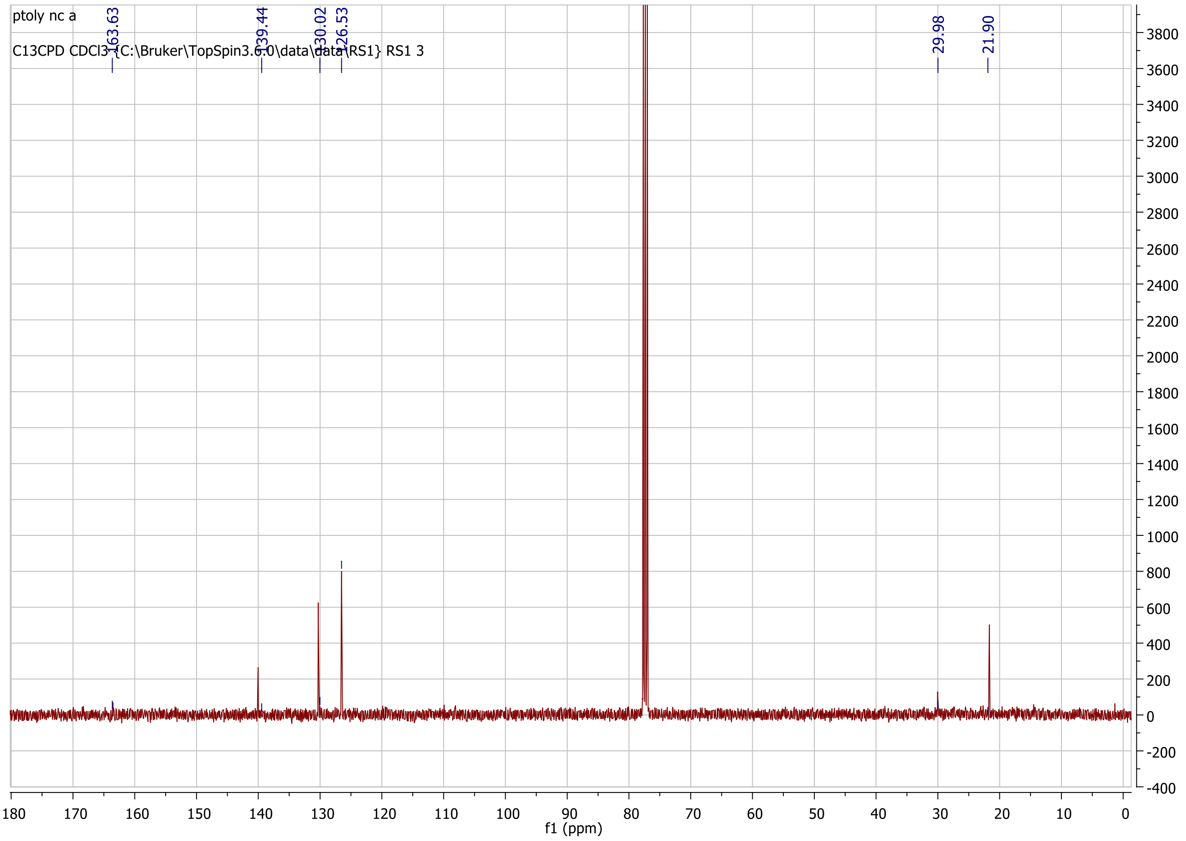

Figure S27. ^{13}C NMR spectrum (101 MHz, $CDCl_3$)

2.14. 1-isocyano-3-methylbenzene

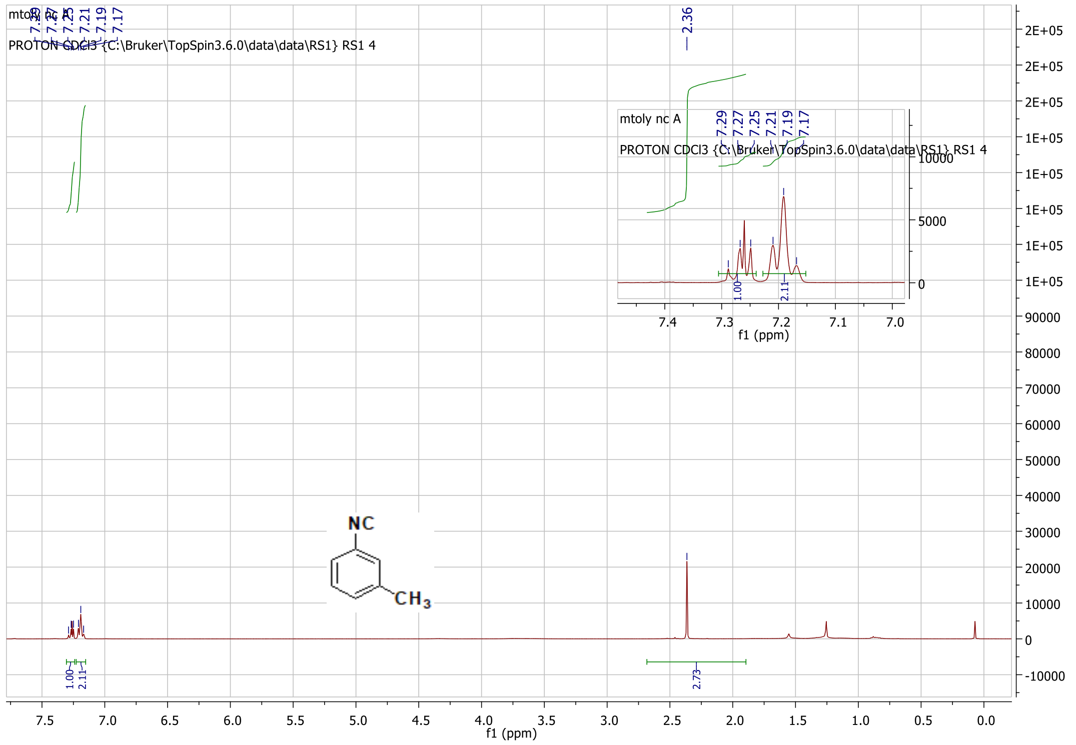

Figure S28. ^{1}H NMR spectrum (400 MHz, $CDCl_3$)

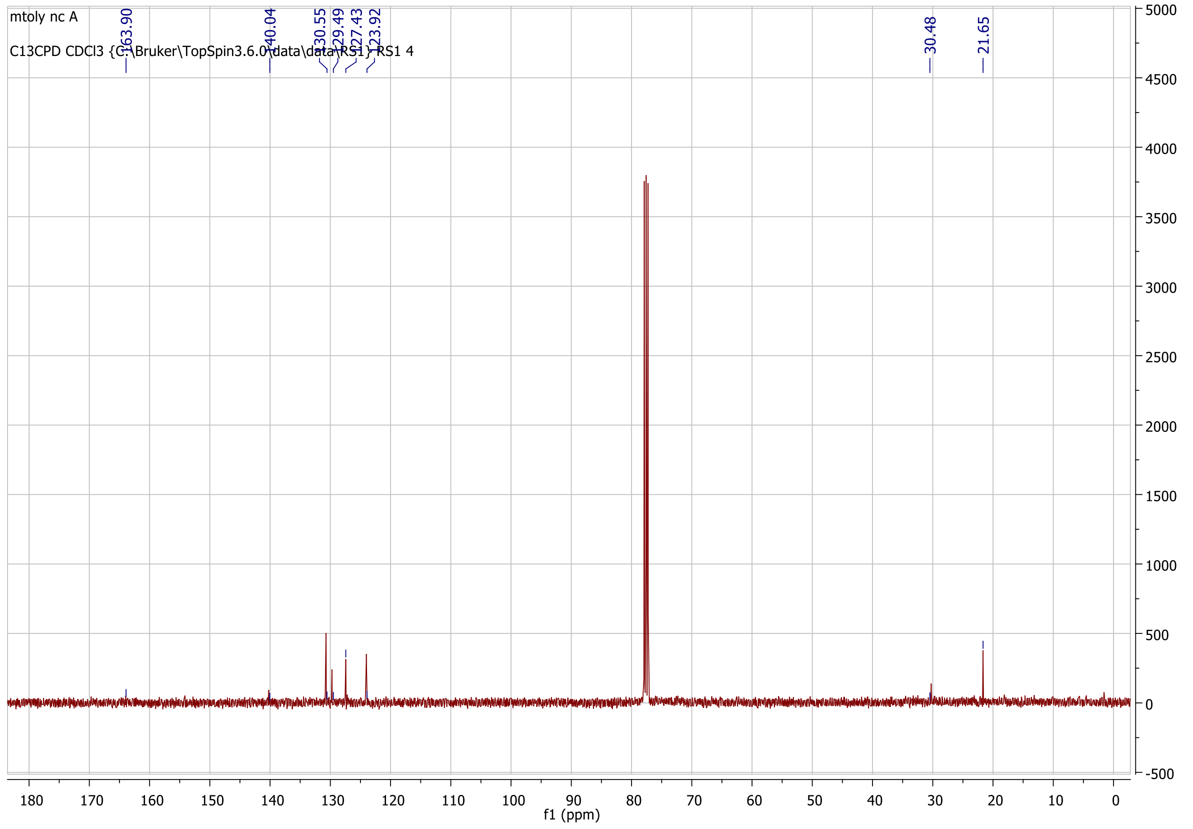

Figure S29. ^{13}C NMR spectrum (101 MHz, $CDCl_3$)

2.15. 3,5-dimethyl phenyl isocyanide

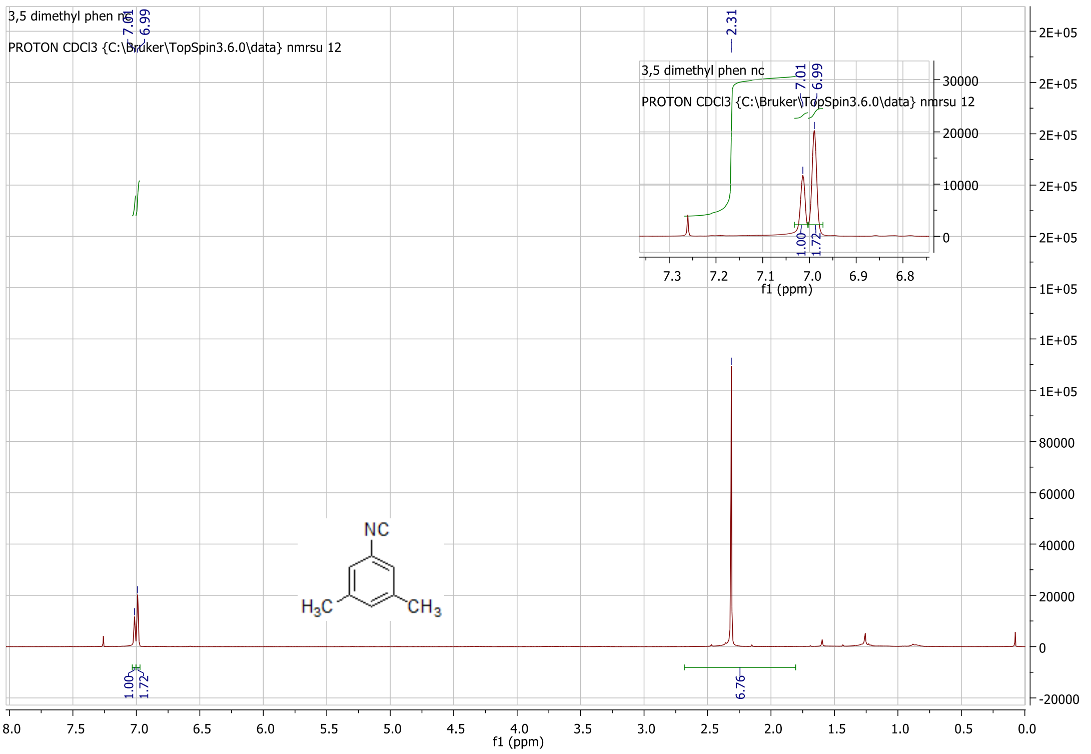

Figure S30. ^{1}H NMR spectrum (400 MHz, $CDCl_3$)

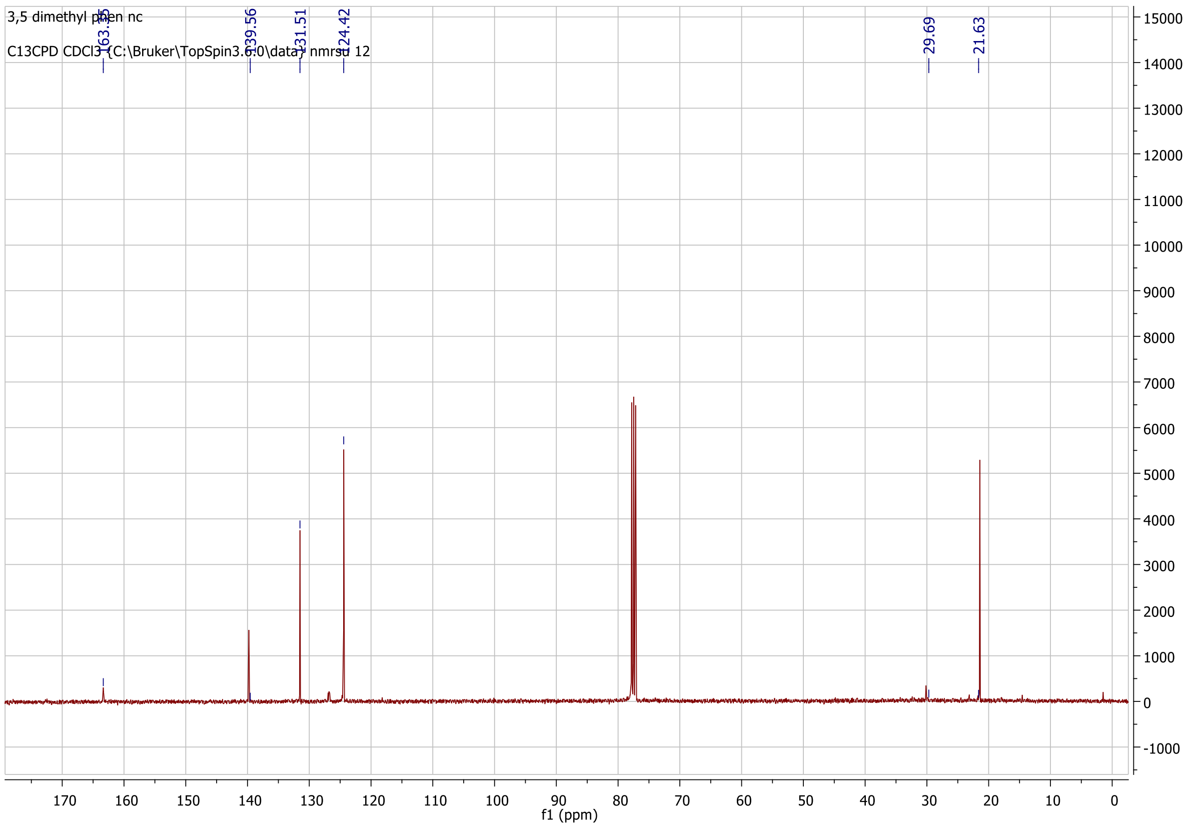

Figure S31. ^{13}C NMR spectrum (101 MHz, $CDCl_3$)

2.16. 2-isocyano-1,3,5-trimethylbenzene

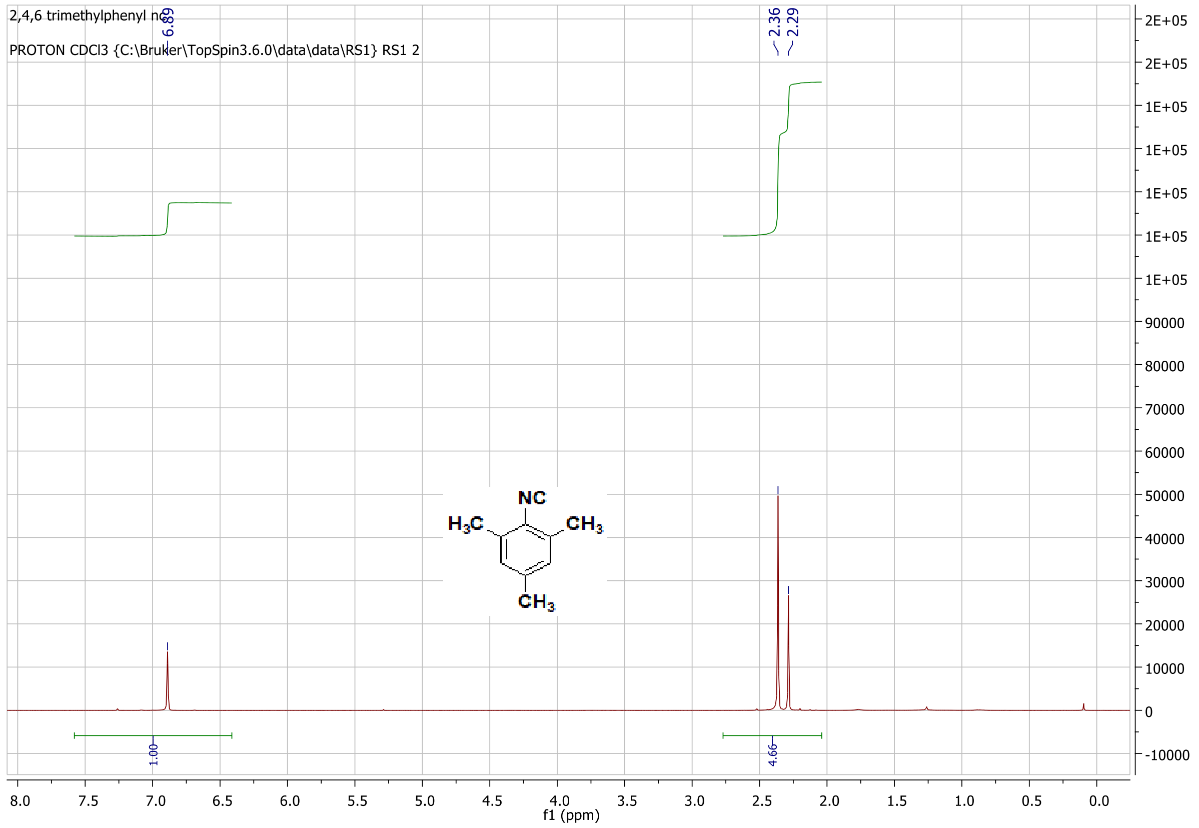

Figure S32. ^{1}H NMR spectrum (400 MHz, $CDCl_3$)

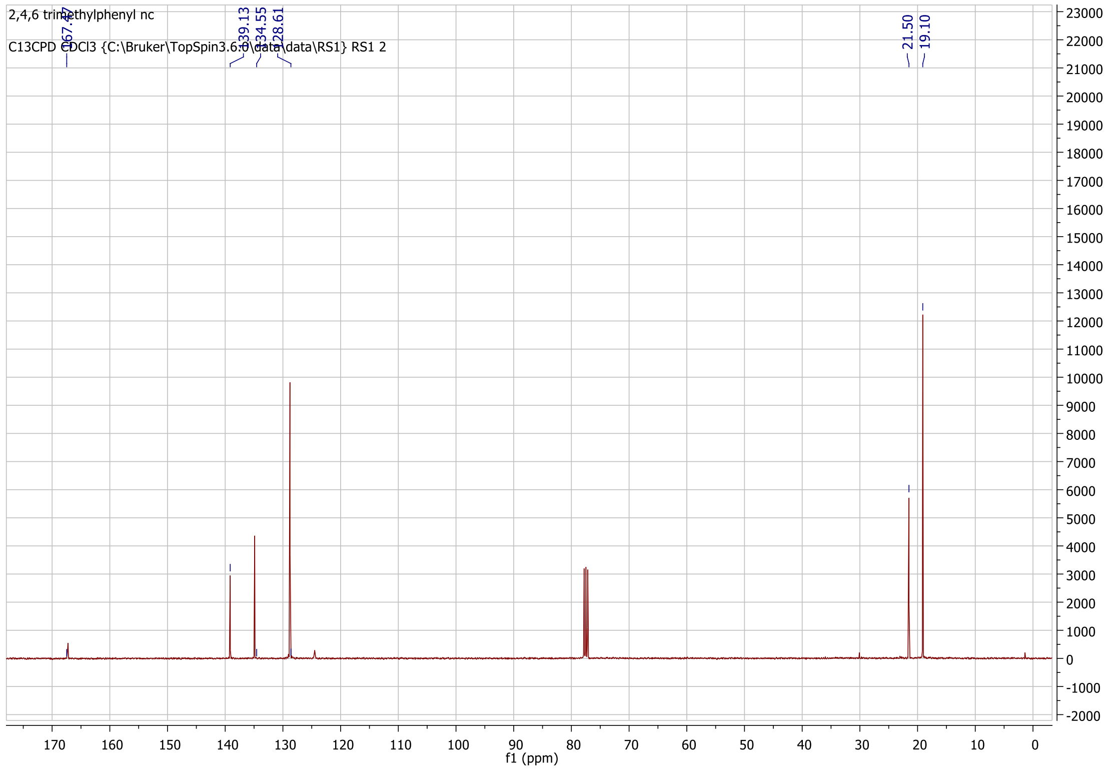

Figure S33. ^{13}C NMR spectrum (101 MHz, $CDCl_3$)

2.17. 3-isocyanobenzonitrile

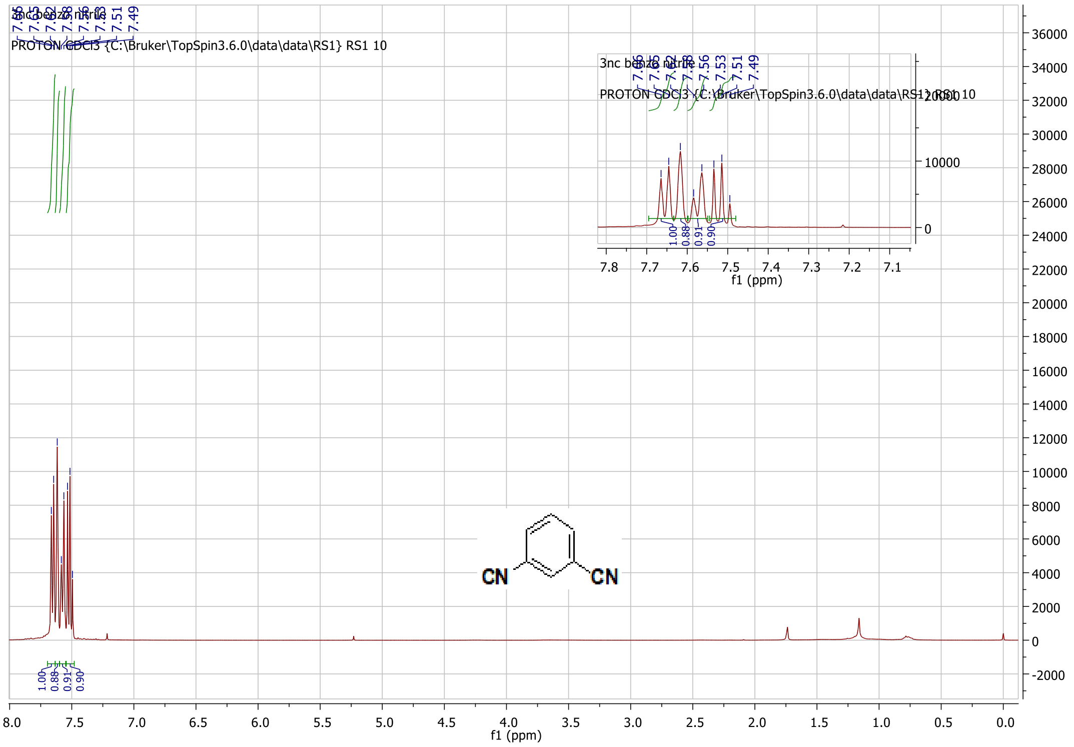

Figure S34. ^{1}H NMR spectrum (400 MHz, $CDCl_3$)

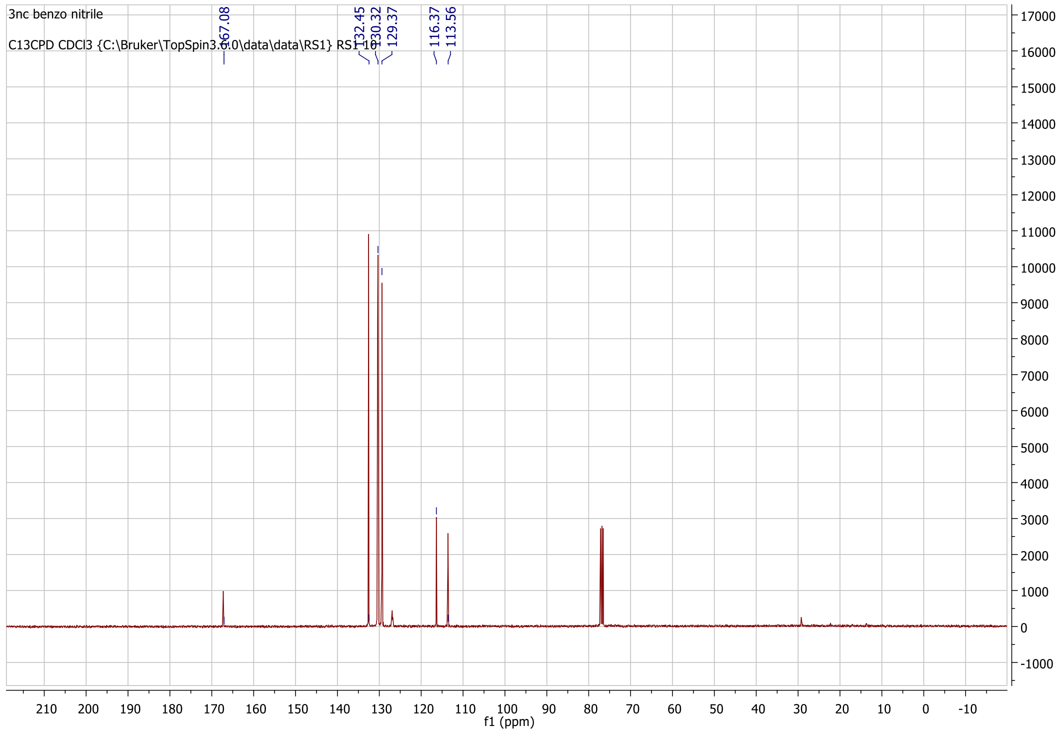

Figure S35. ^{13}C NMR spectrum (101 MHz, $CDCl_3$)

2.18. 1-(isocyanomethyl)-4-methoxybenzene

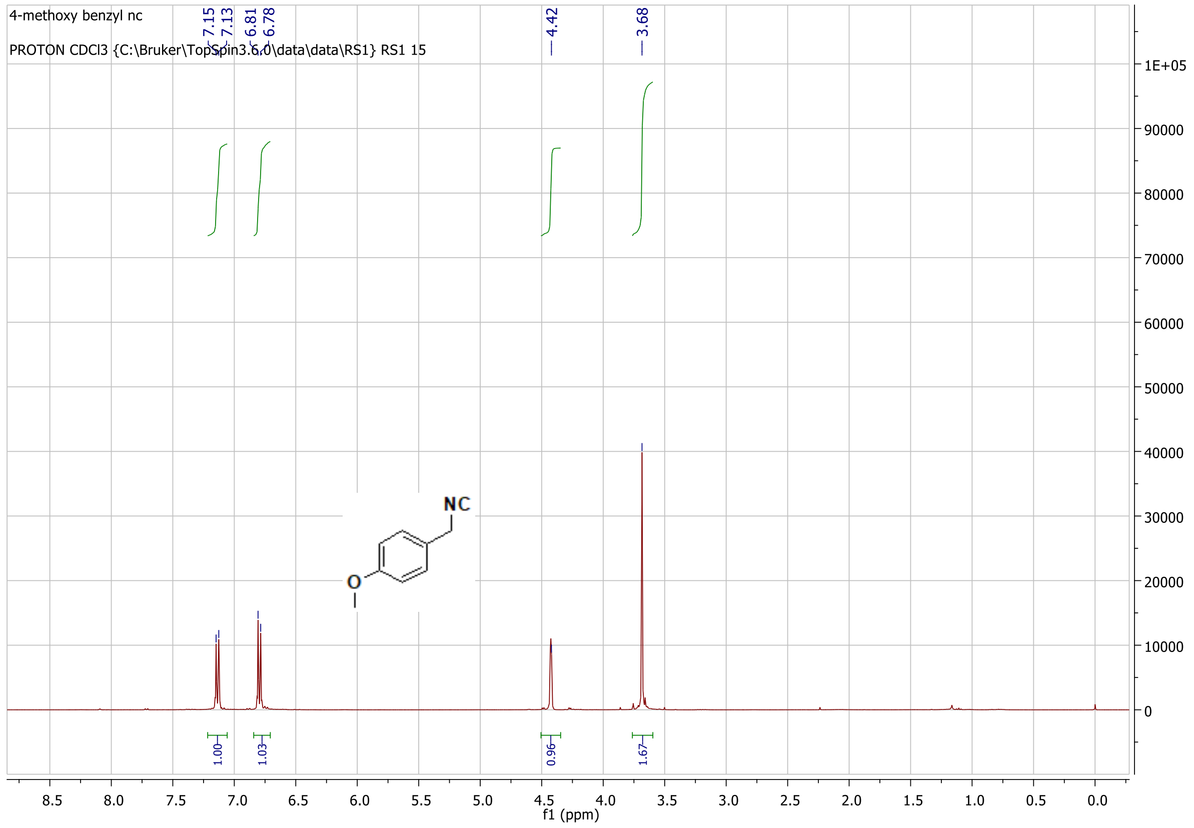

Figure S36. ^{1}H NMR spectrum (400 MHz, $CDCl_3$)

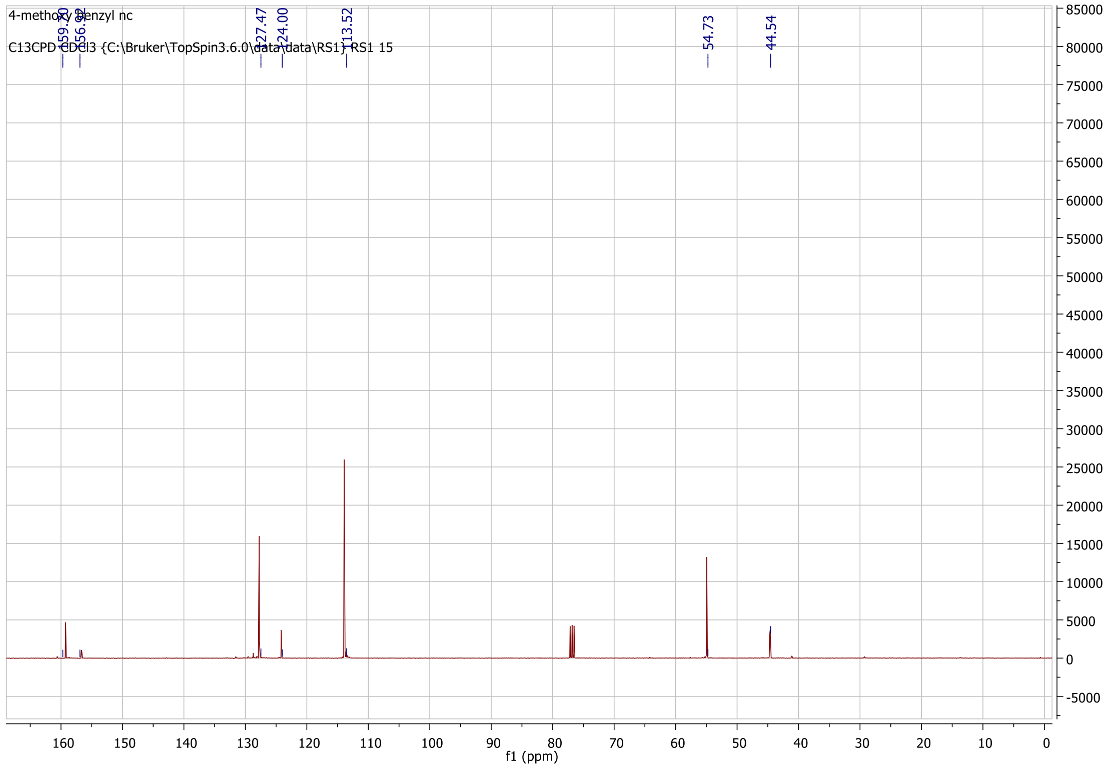

Figure S37. ^{13}C NMR spectrum (101 MHz, $CDCl_3$)

2.19. 1-isocyano-4-methoxy benzene

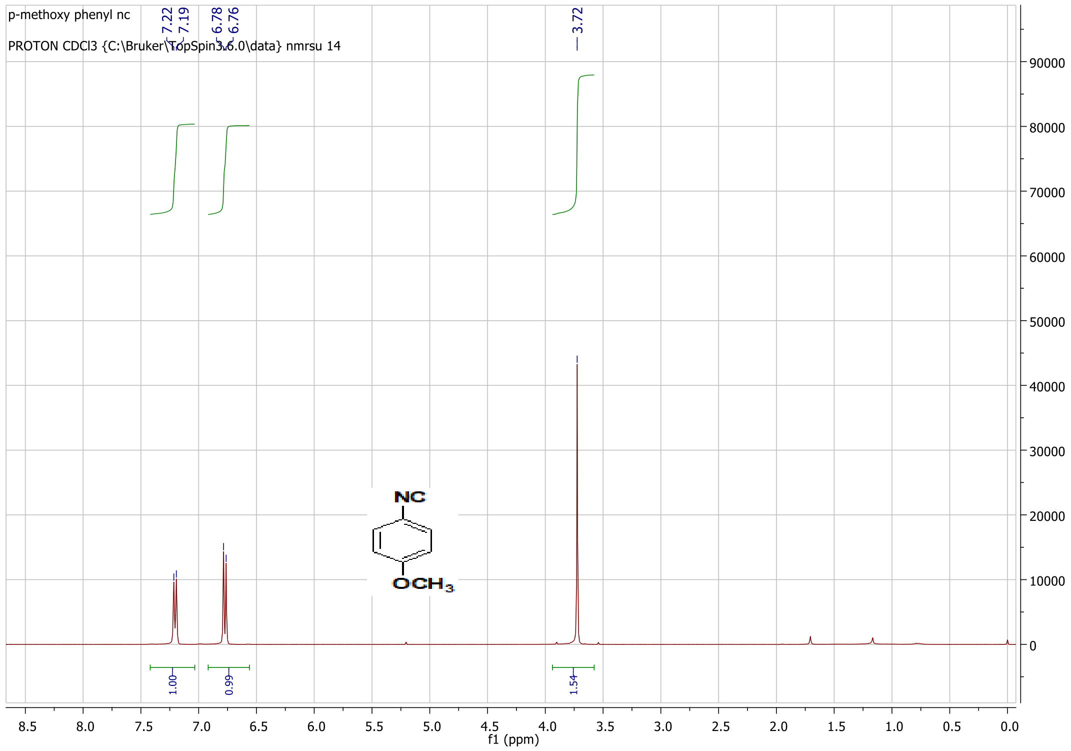

Figure S38. ^{1}H NMR spectrum (400 MHz, $CDCl_3$)

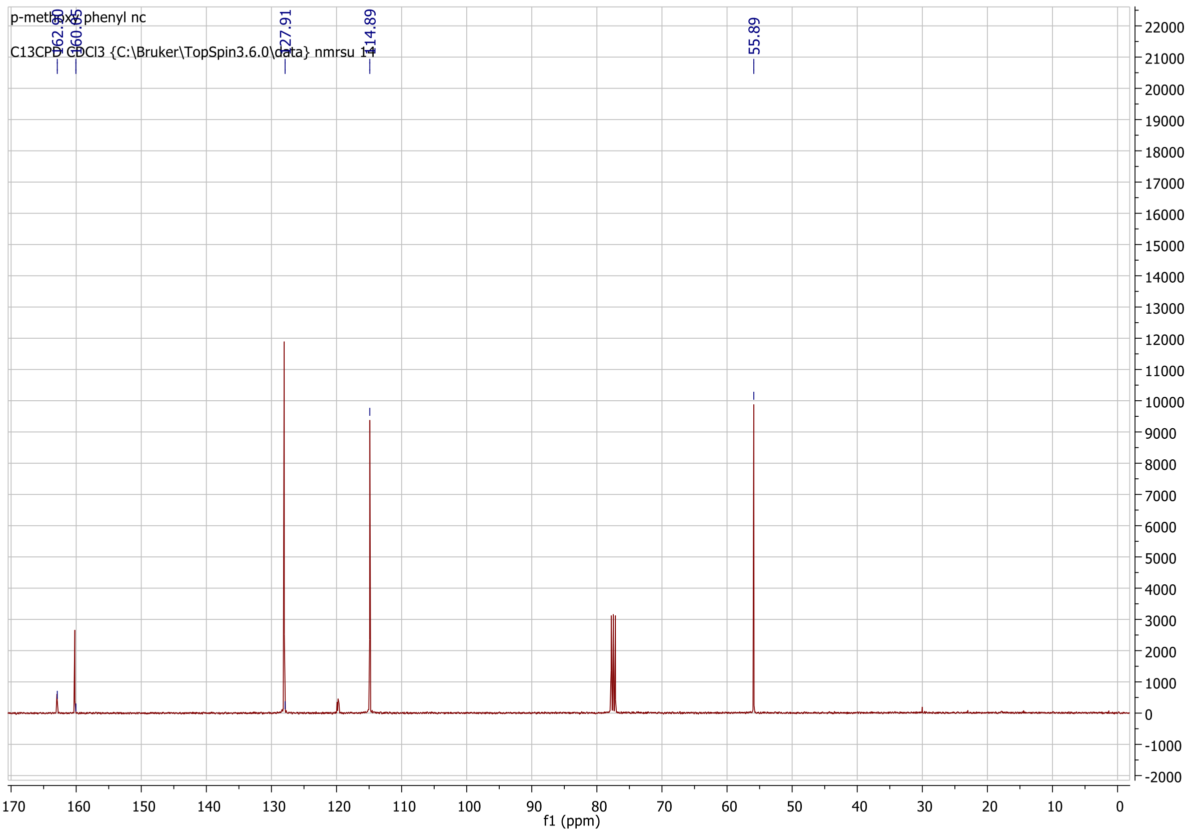

Figure S39. ^{13}C NMR spectrum (101 MHz, $CDCl_3$)

2.20. 1-(isocyanomethyl)-2-methoxybenzene

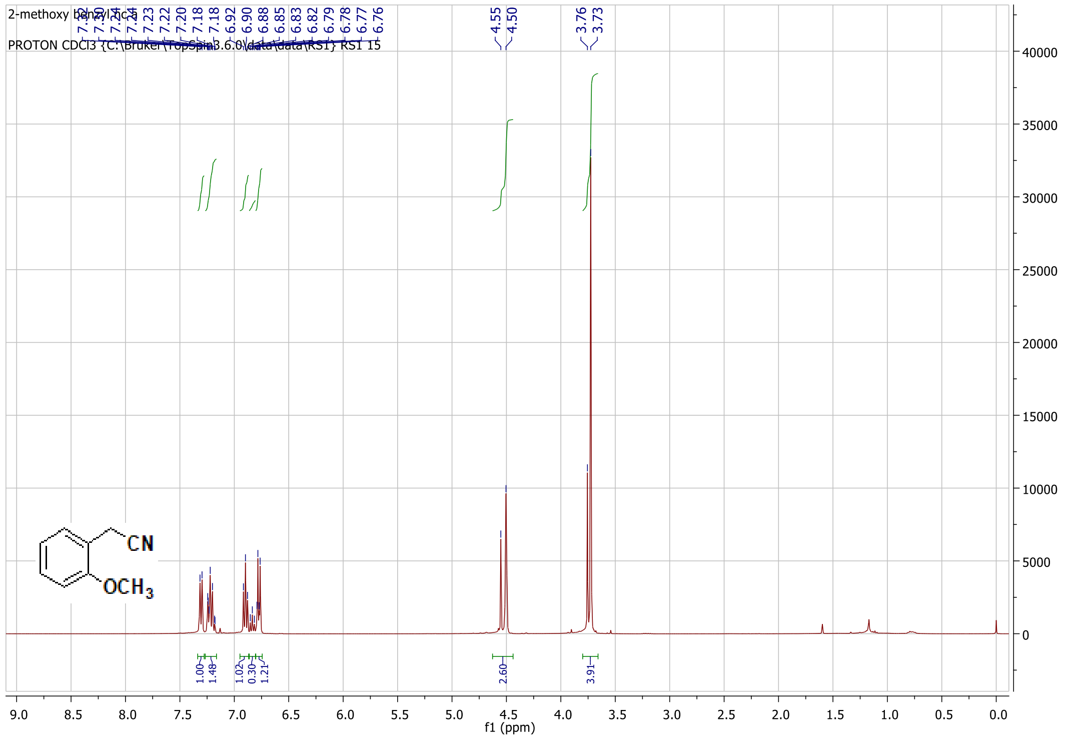

Figure S40. ^{1}H NMR spectrum (400 MHz, $CDCl_3$)

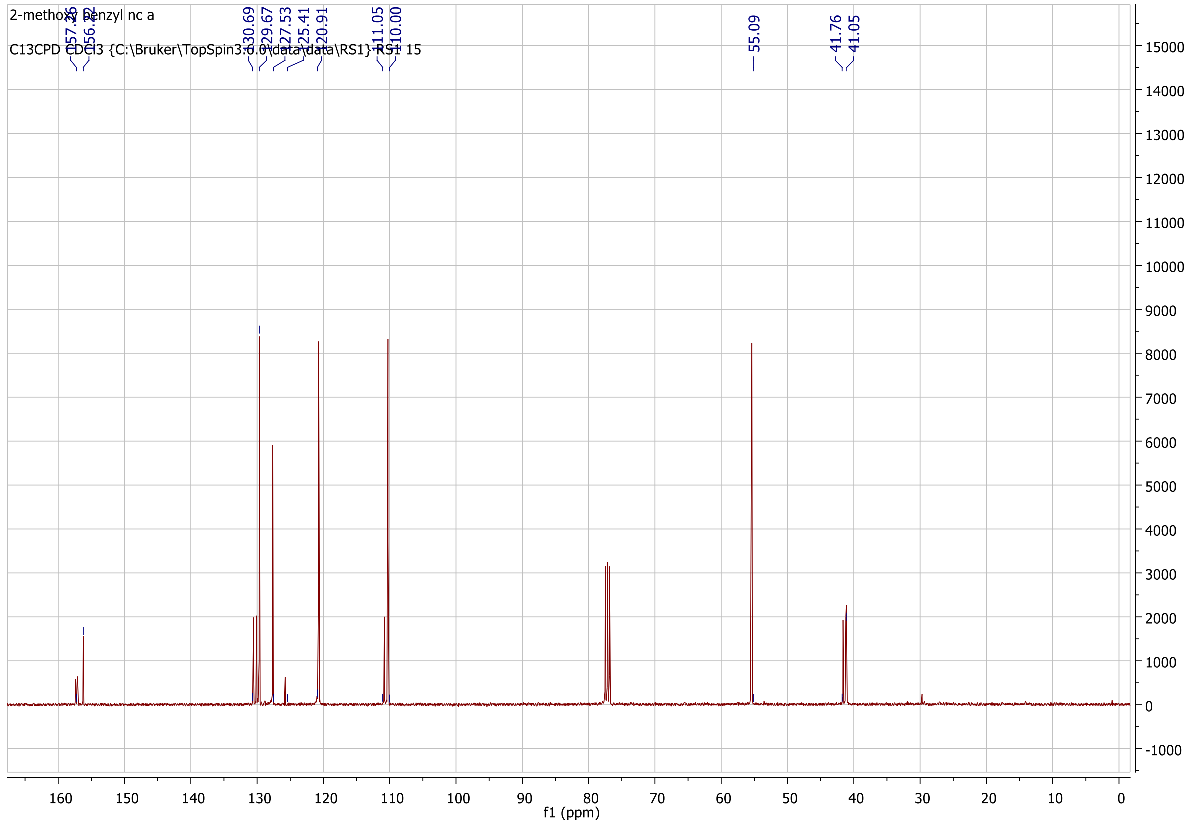

Figure S41. ^{13}C NMR spectrum (101 MHz, $CDCl_3$)

2.21. 1,2-diisocyanobenzene

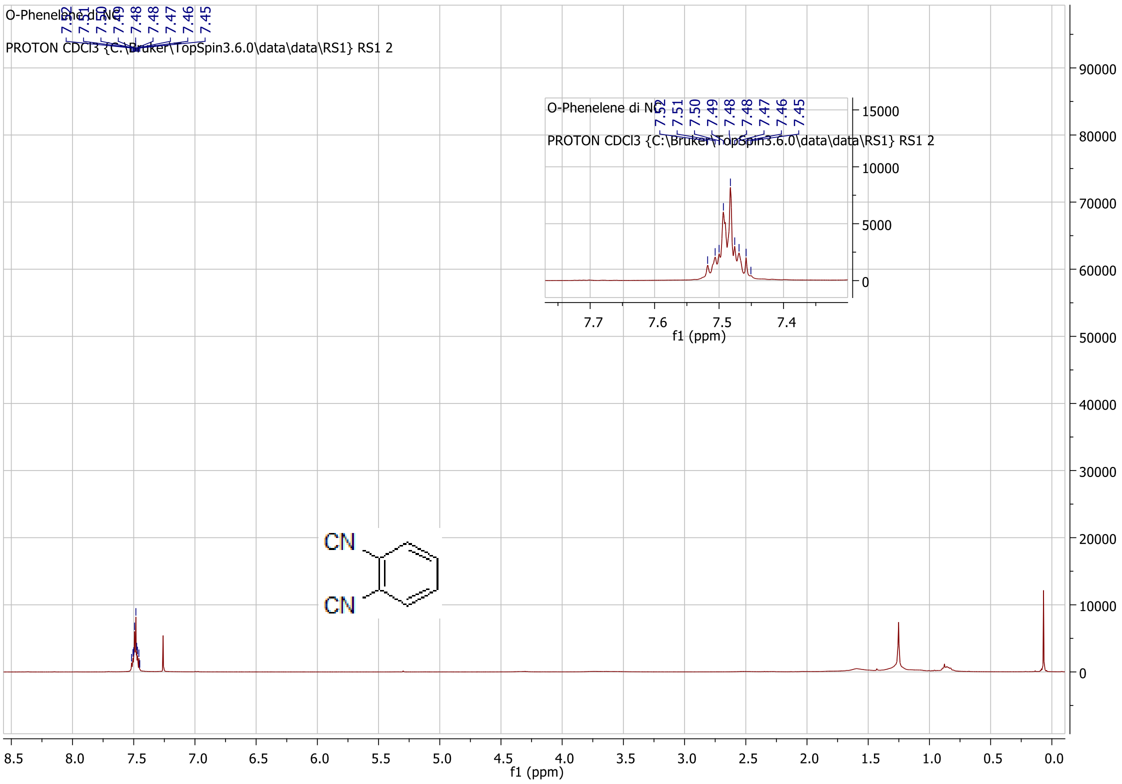

Figure S42. ^{1}H NMR spectrum (400 MHz, $CDCl_3$)

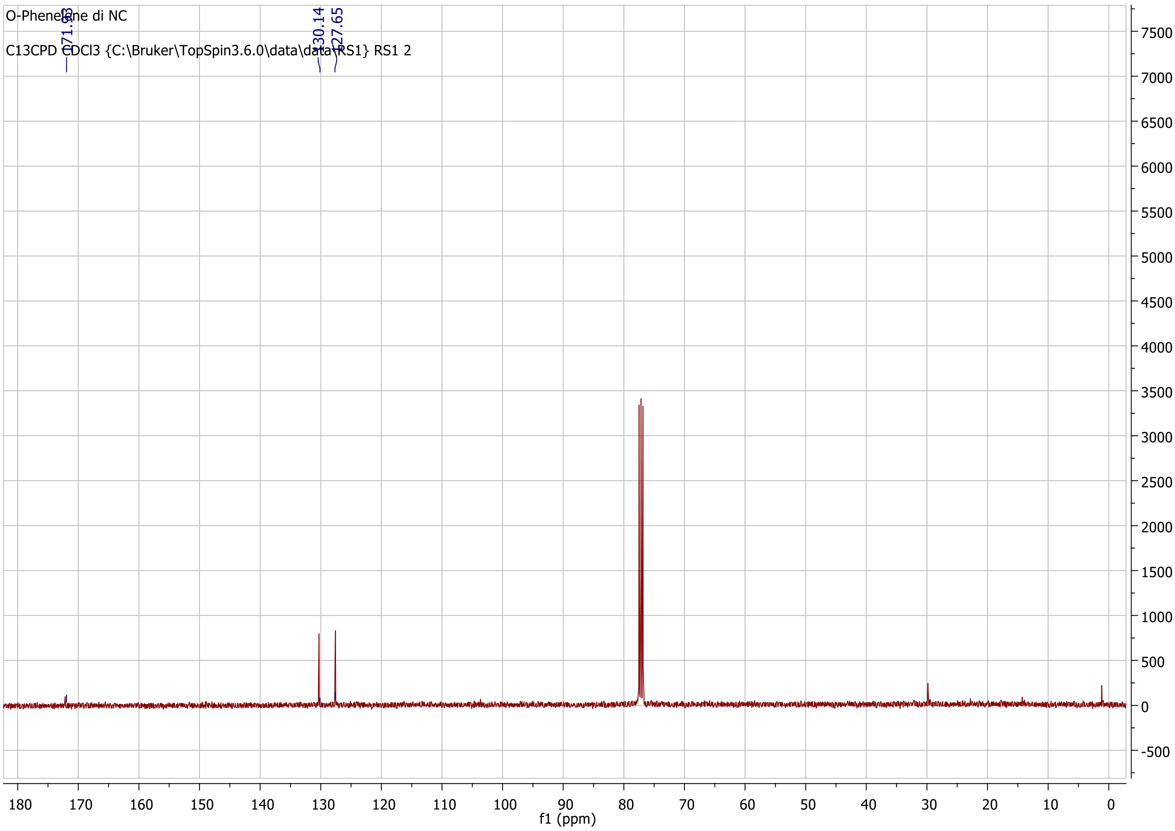

Figure S43. ^{13}C NMR spectrum (101 MHz, $CDCl_3$)

2.22. Ethyl-2 isocyanobenzoate

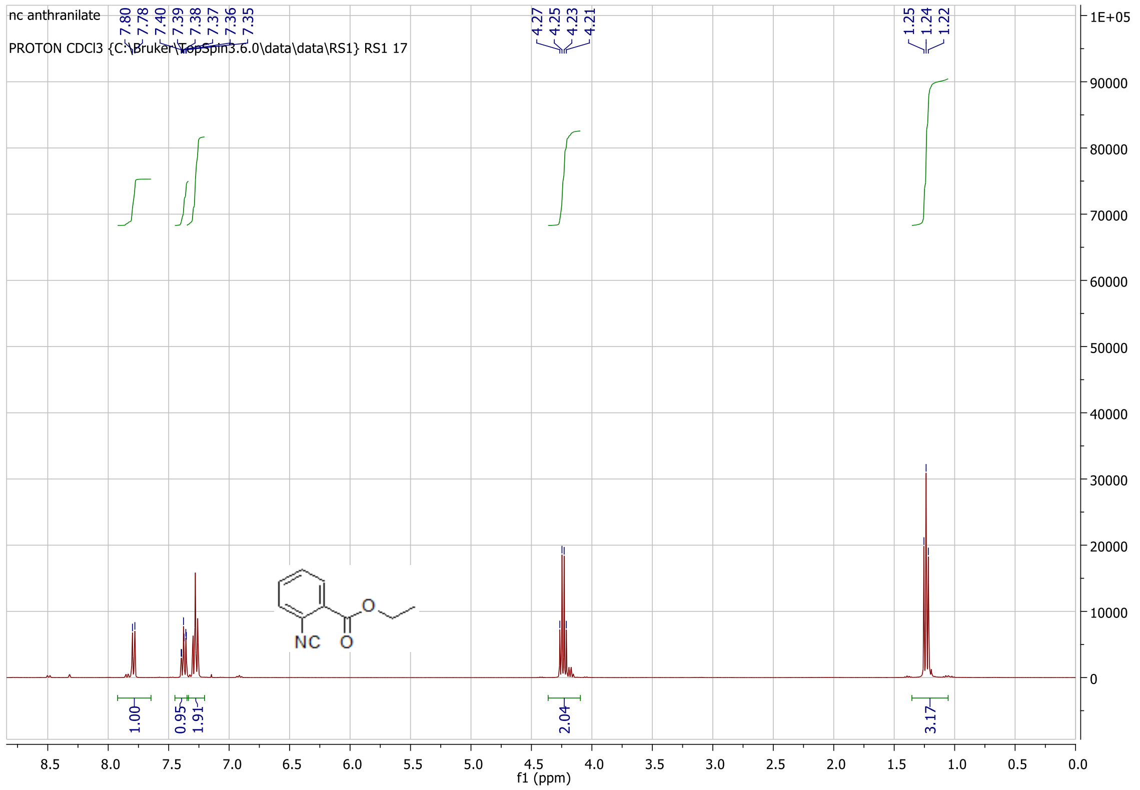

Figure S44. ^{1}H NMR spectrum (400 MHz, $CDCl_3$)

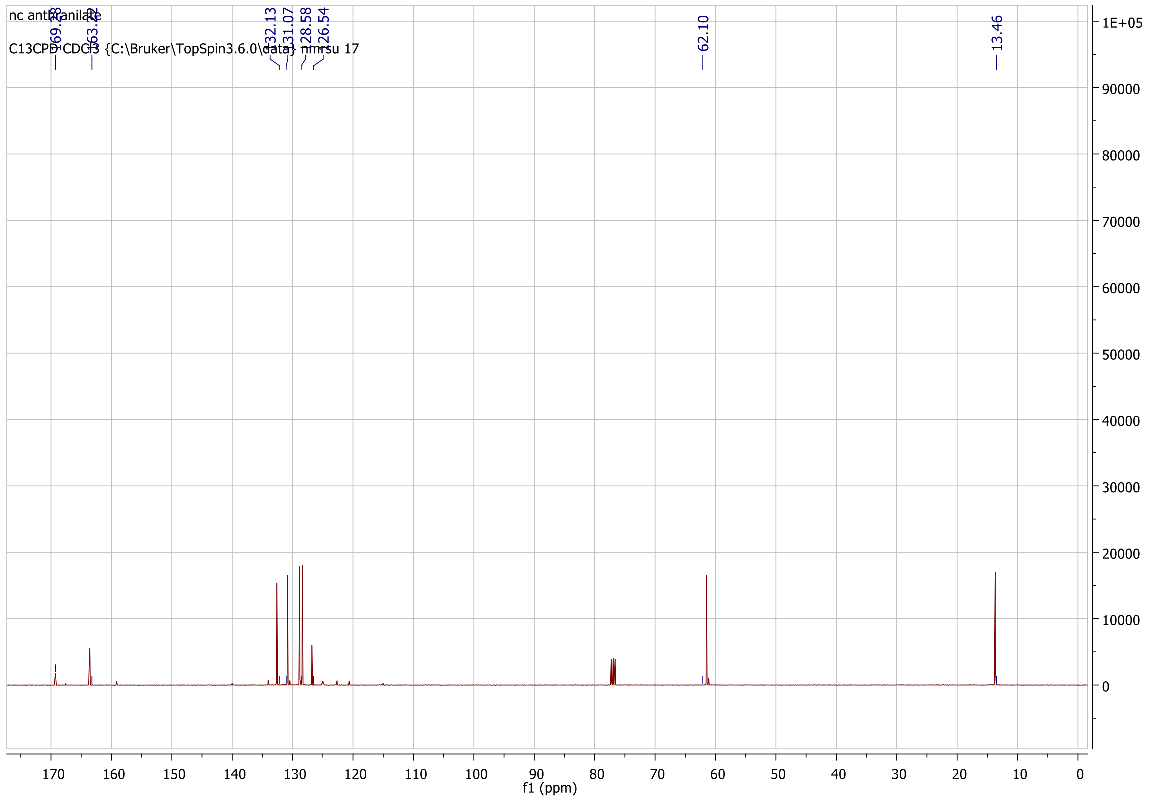

Figure S45. ^{13}C NMR spectrum (101 MHz, $CDCl_3$)

2.23. 1-isocyanonaphthalene

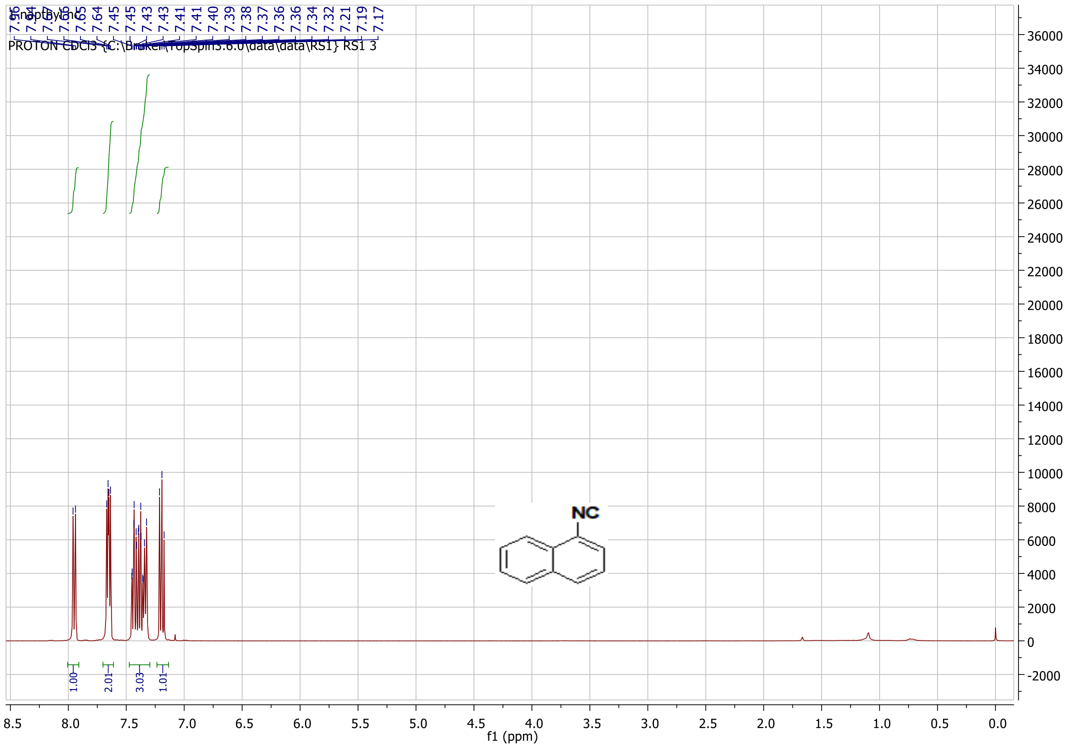

Figure S46. ^{1}H NMR spectrum (400 MHz, $CDCl_3$)

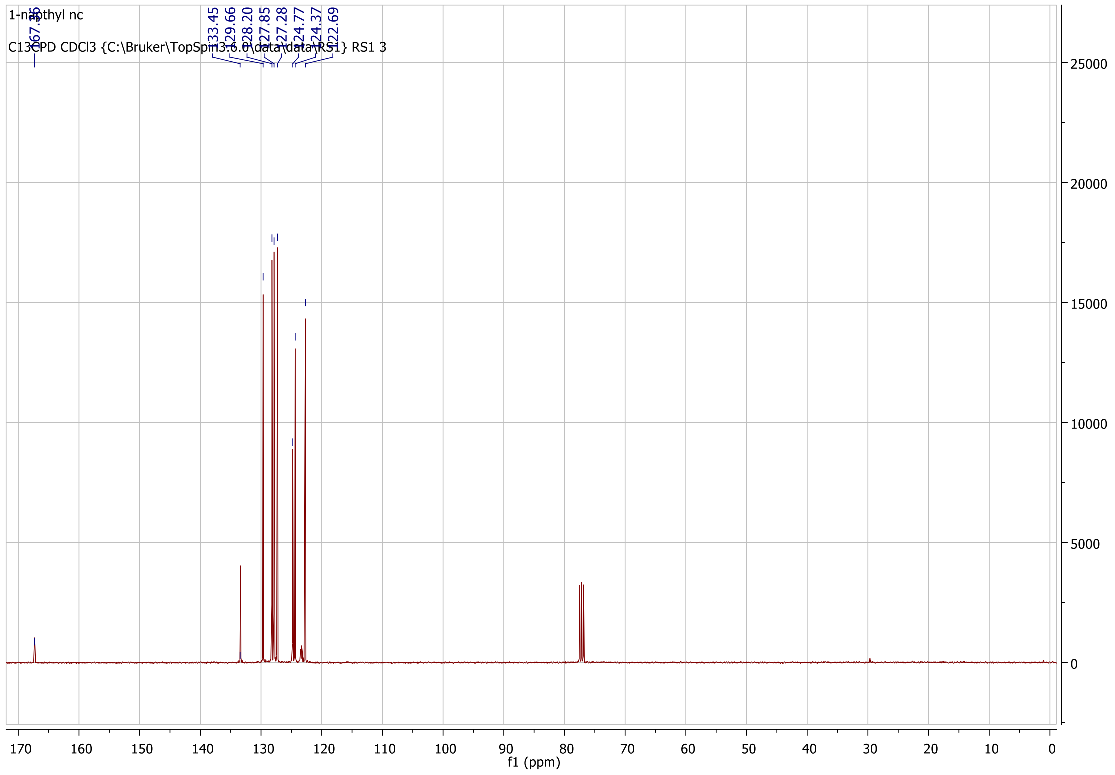

Figure S47. ^{13}C NMR spectrum (101 MHz, $CDCl_3$)

2.24. 1,5-diisocyanonaphthalene

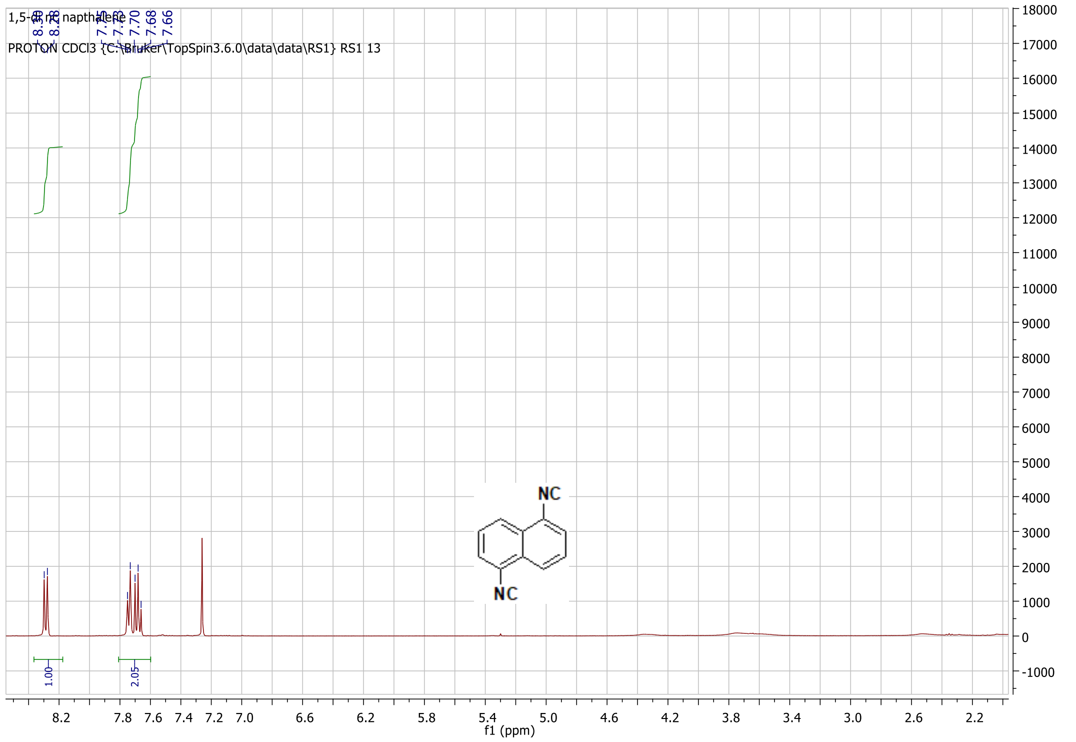

Figure S48. ^{1}H NMR spectrum (400 MHz, $CDCl_3$)

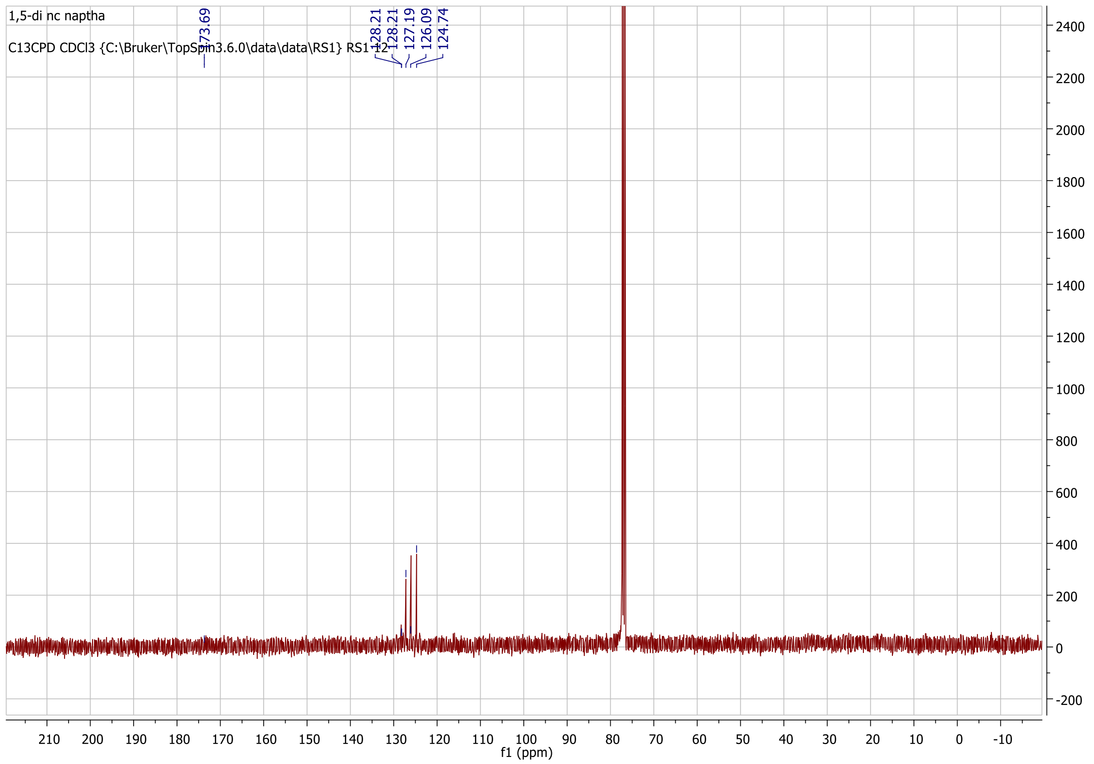

Figure S49. ^{13}C NMR spectrum (101 MHz, $CDCl_3$)

2.25. 2-Isocyano anthracene-9, 10 dione

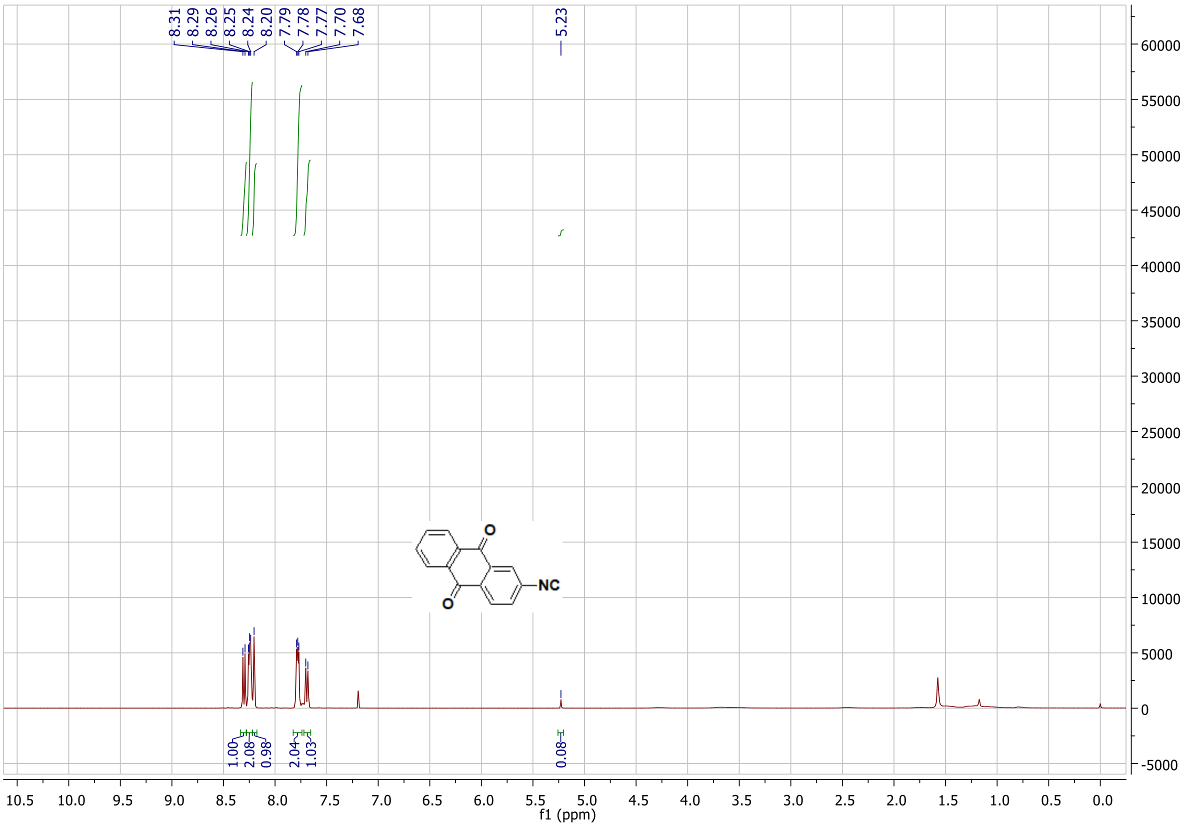

Figure S50. ^{1}H NMR spectrum (400 MHz, CD_3CN)

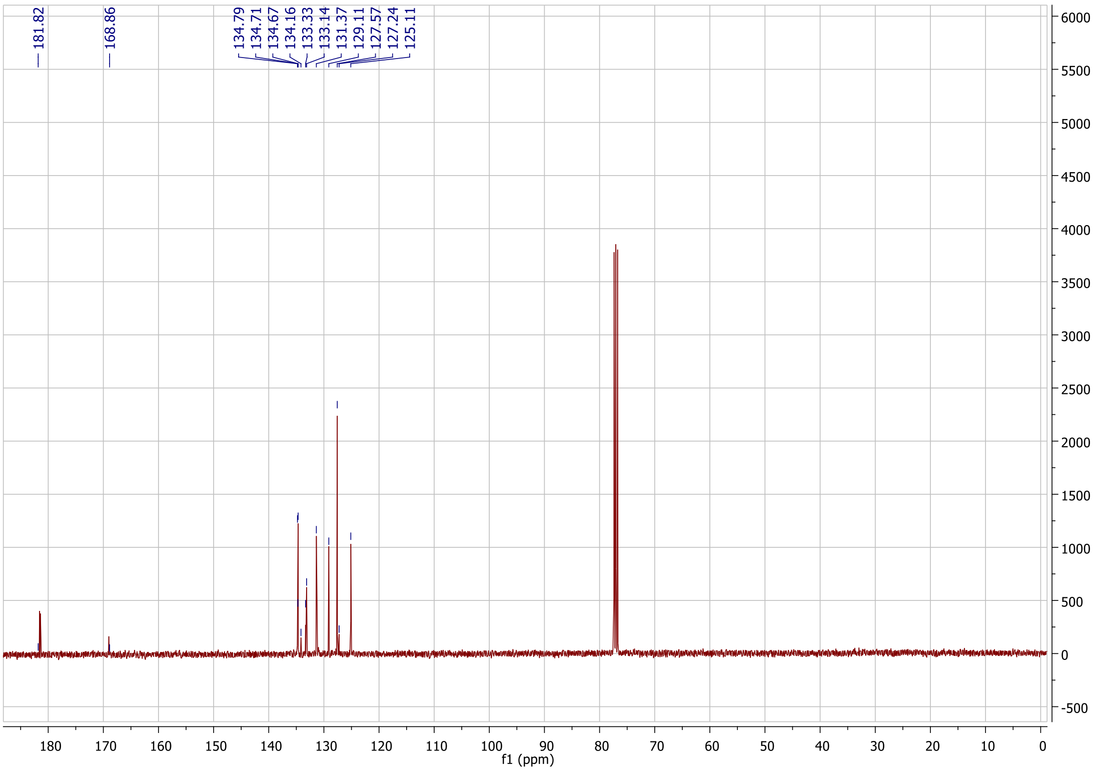

Figure S51. ^{13}C NMR spectrum (101 MHz, CD_3CN)

2.26. 4-Isocyanobenzoic acid

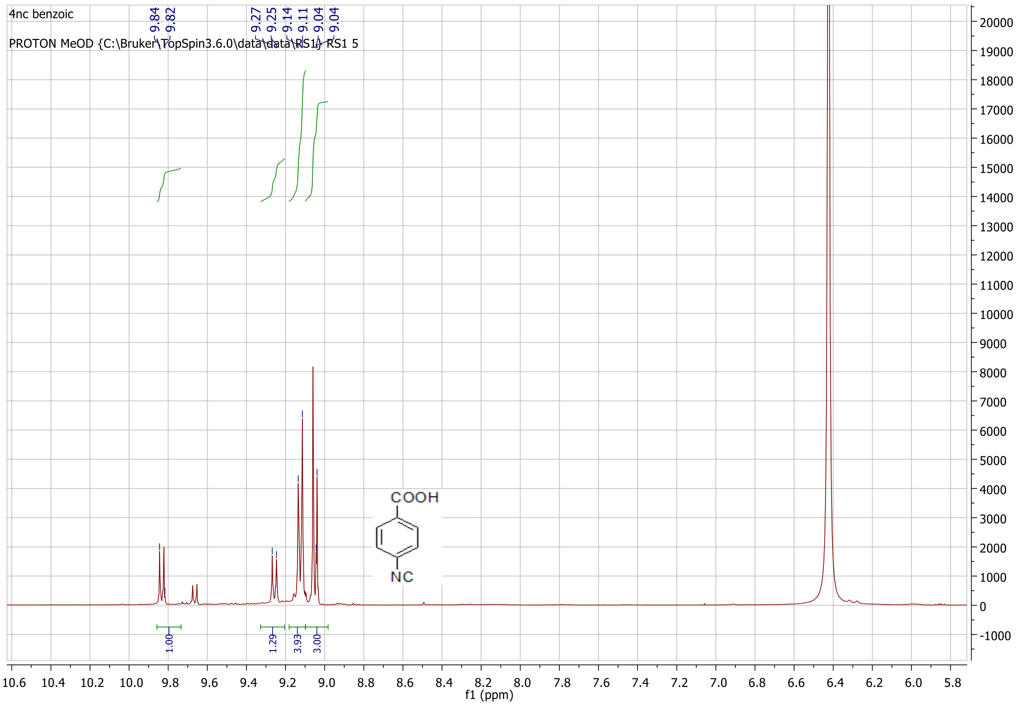

Figure S52. ^{1}H NMR spectrum (400 MHz, MeOD)

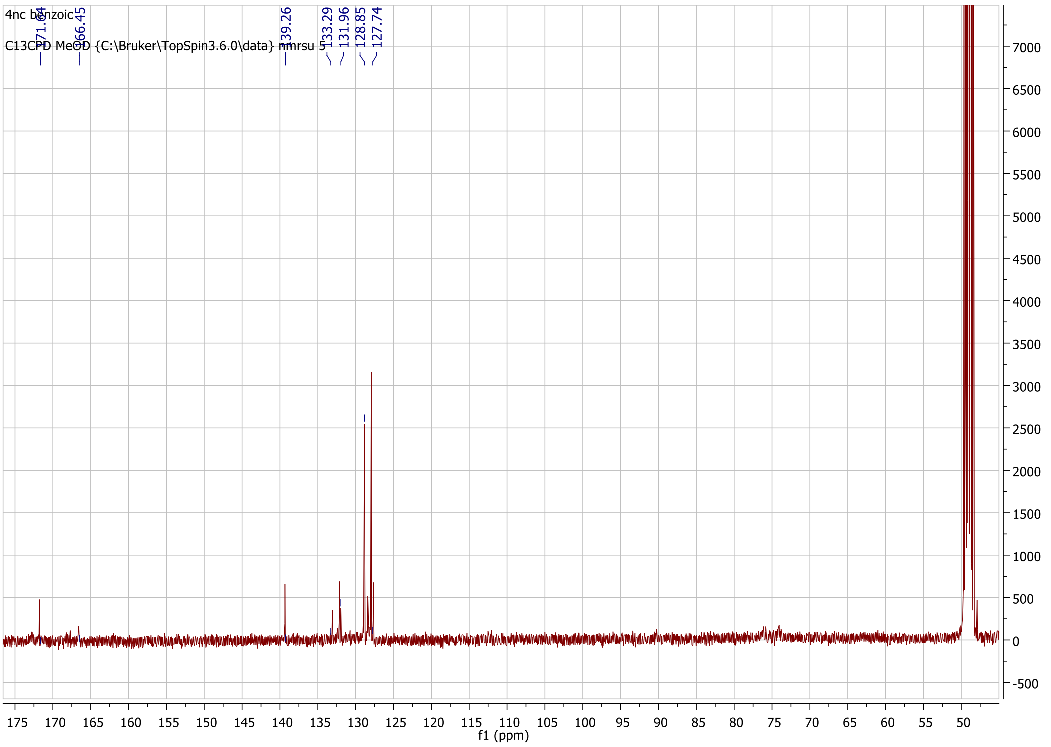

Figure S53. ^{13}C NMR spectrum (101 MHz, MeOD)

2.27. 4-isocyano phenol

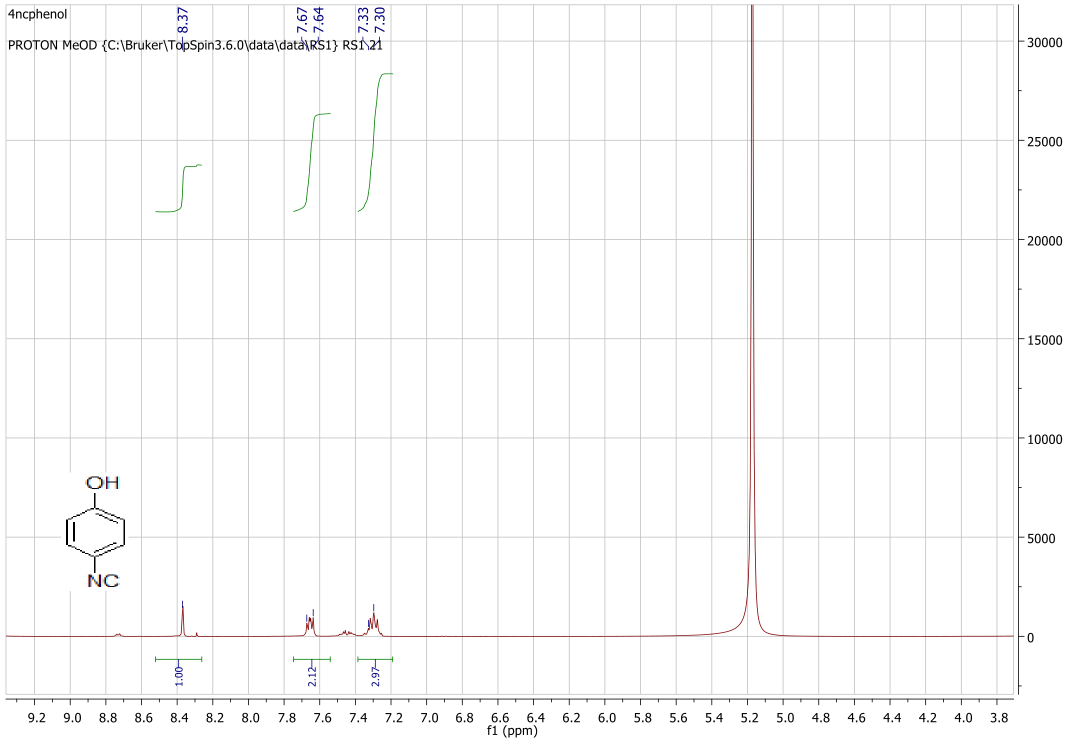

Figure S54. ^{1}H NMR spectrum (400 MHz, MeOD)

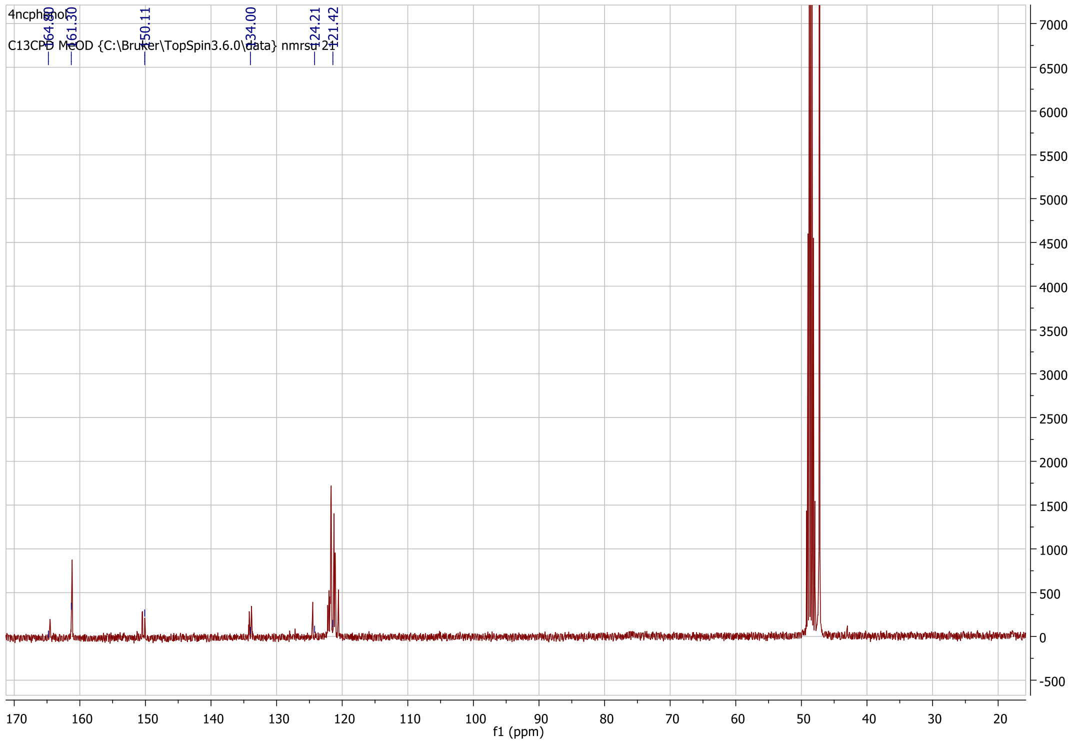

Figure S55. ^{13}C NMR spectrum (101 MHz, MeOD)

2.28. (5-chloro-2-isocyanophenyl)(2-fluorophenyl)methanone

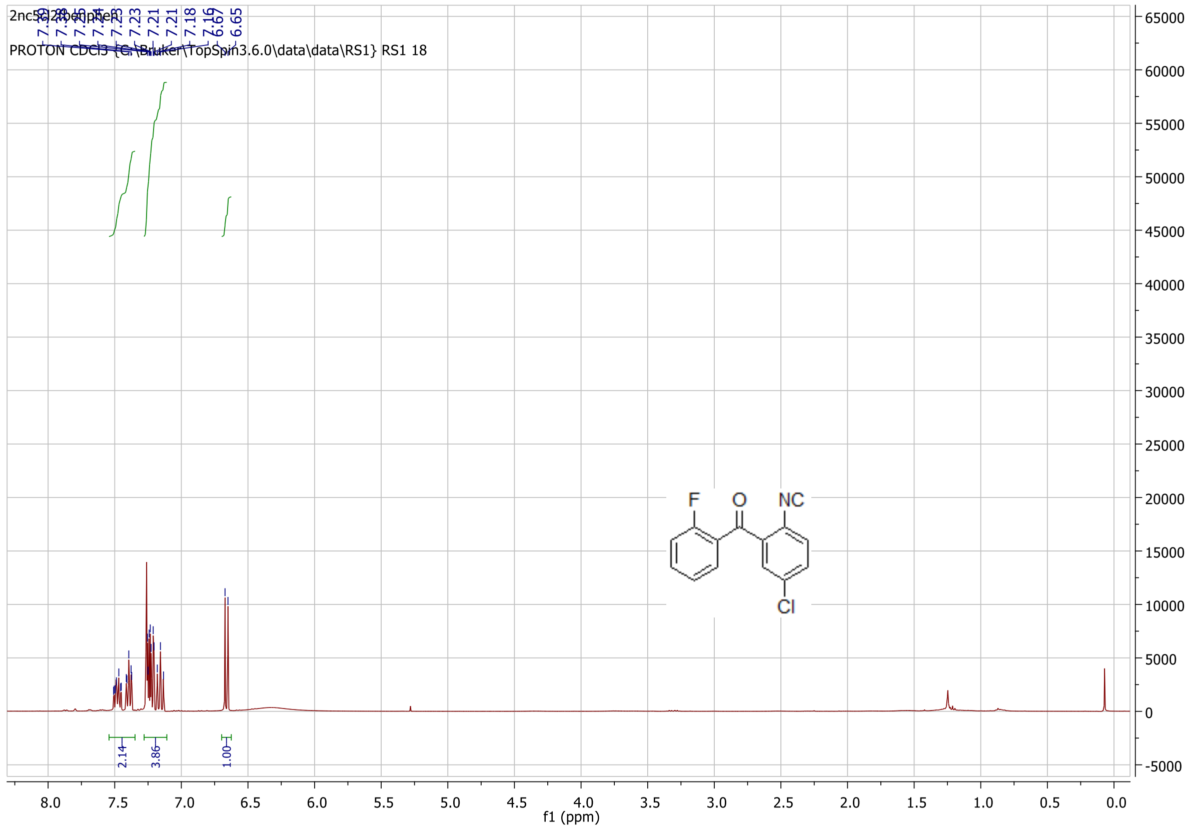

Figure S56. ^{1}H NMR spectrum (400 MHz, $CDCl_3$)

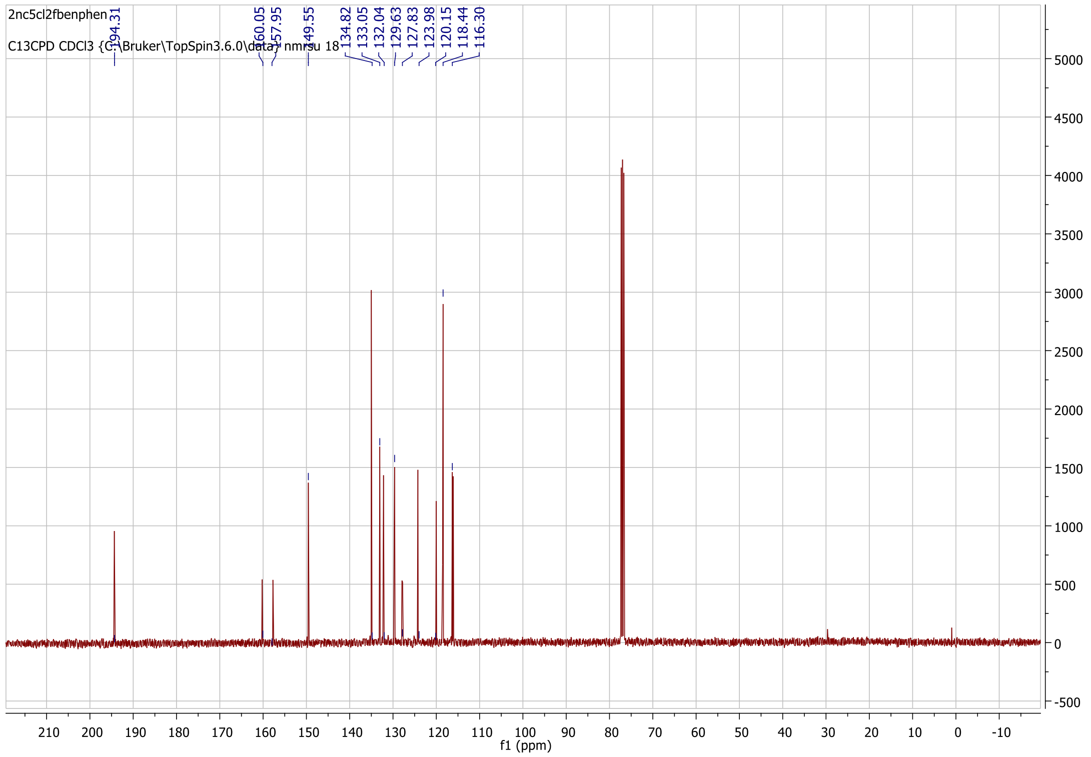

Figure S57. ^{13}C NMR spectrum (101 MHz, $CDCl_3$)

2.29. Isocyano(pyridine-3-yl)methanone

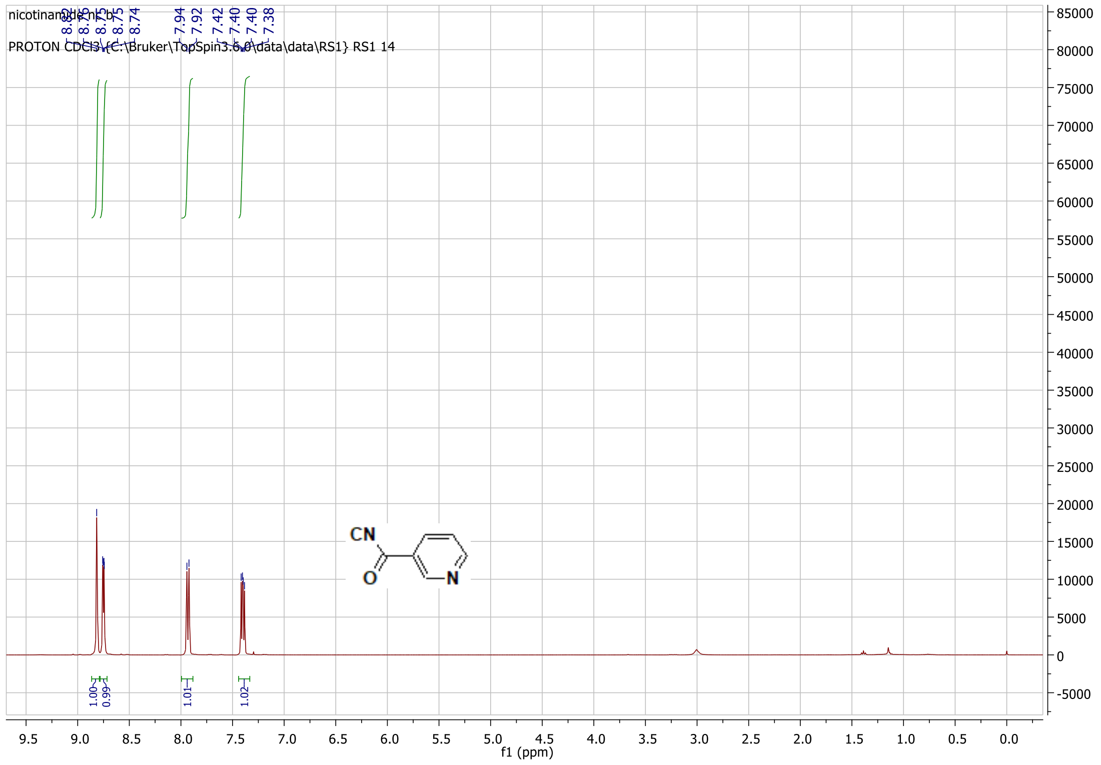

Figure S58. ^{1}H NMR spectrum (400 MHz, $CDCl_3$)

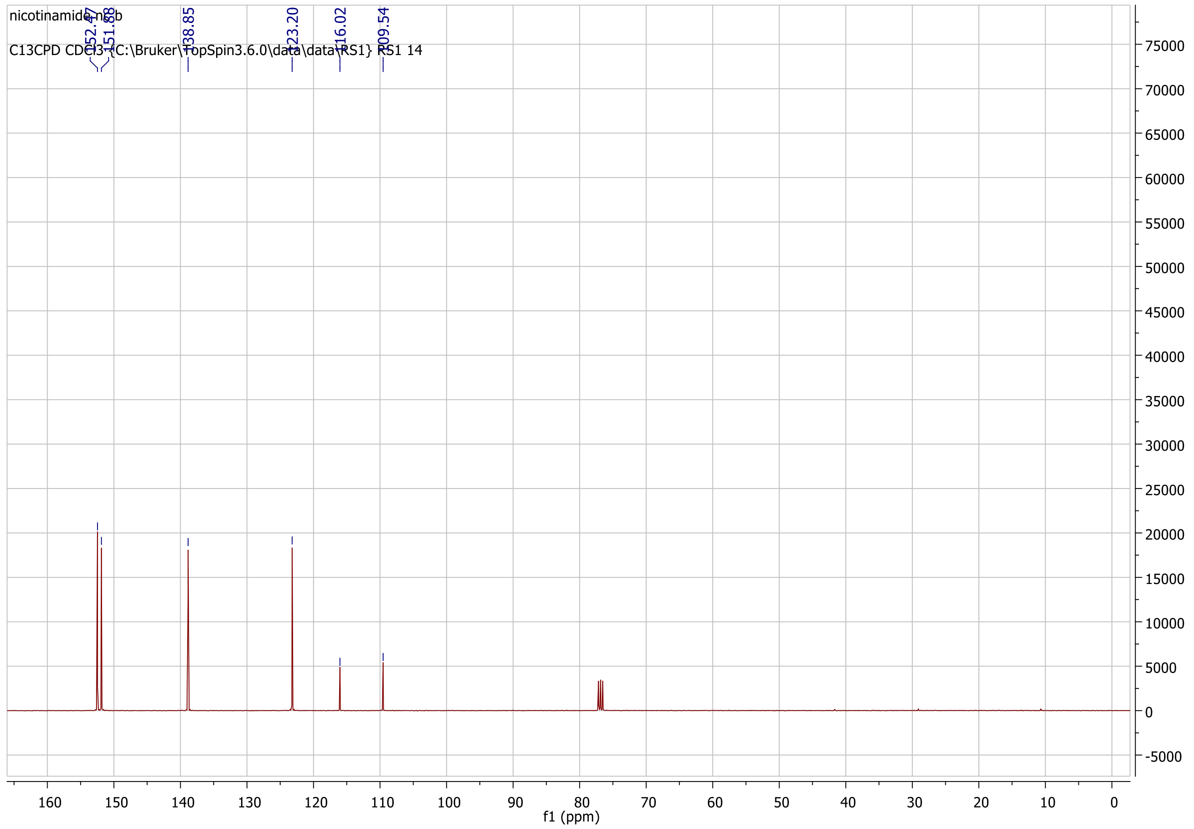

Figure S59. ^{13}C NMR spectrum (101 MHz, $CDCl_3$)

2.30. 2-Isocyano Thiazole

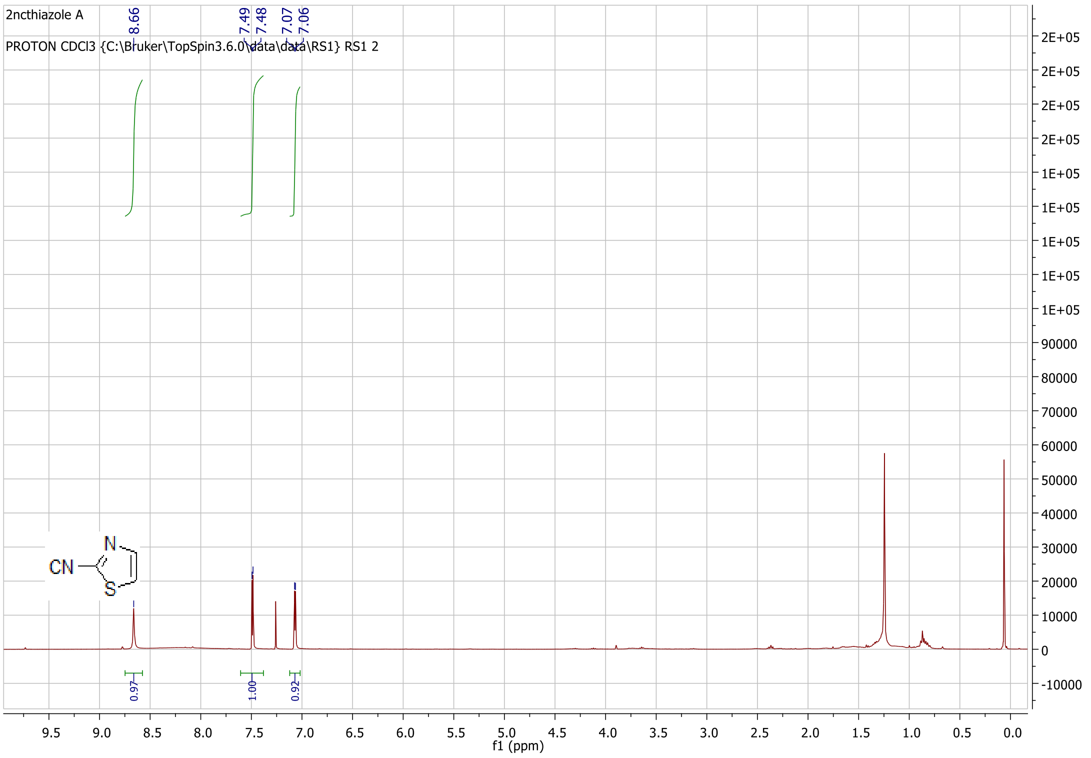

Figure S60. ^{1}H NMR spectrum (400 MHz, $CDCl_3$)

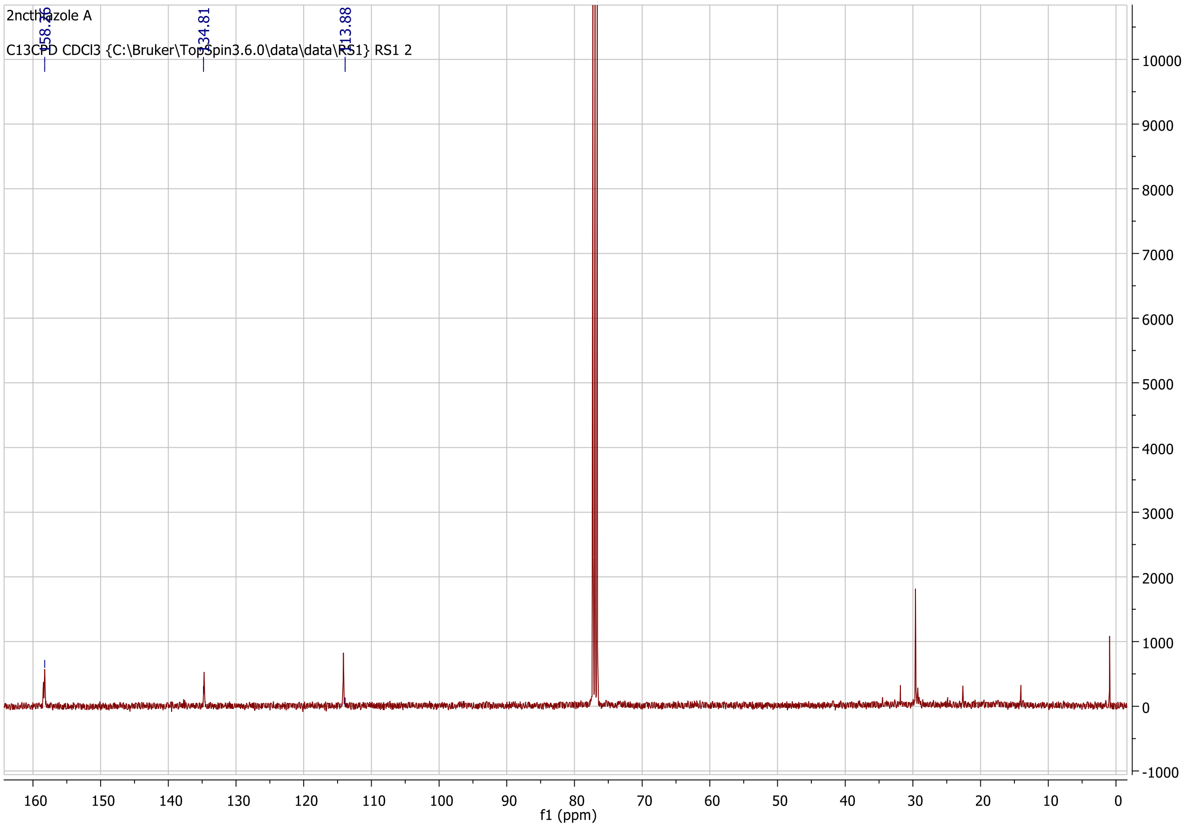

Figure S61. ^{13}C NMR spectrum (101 MHz, $CDCl_3$)

2.31. 4-isocyano-antipyrine

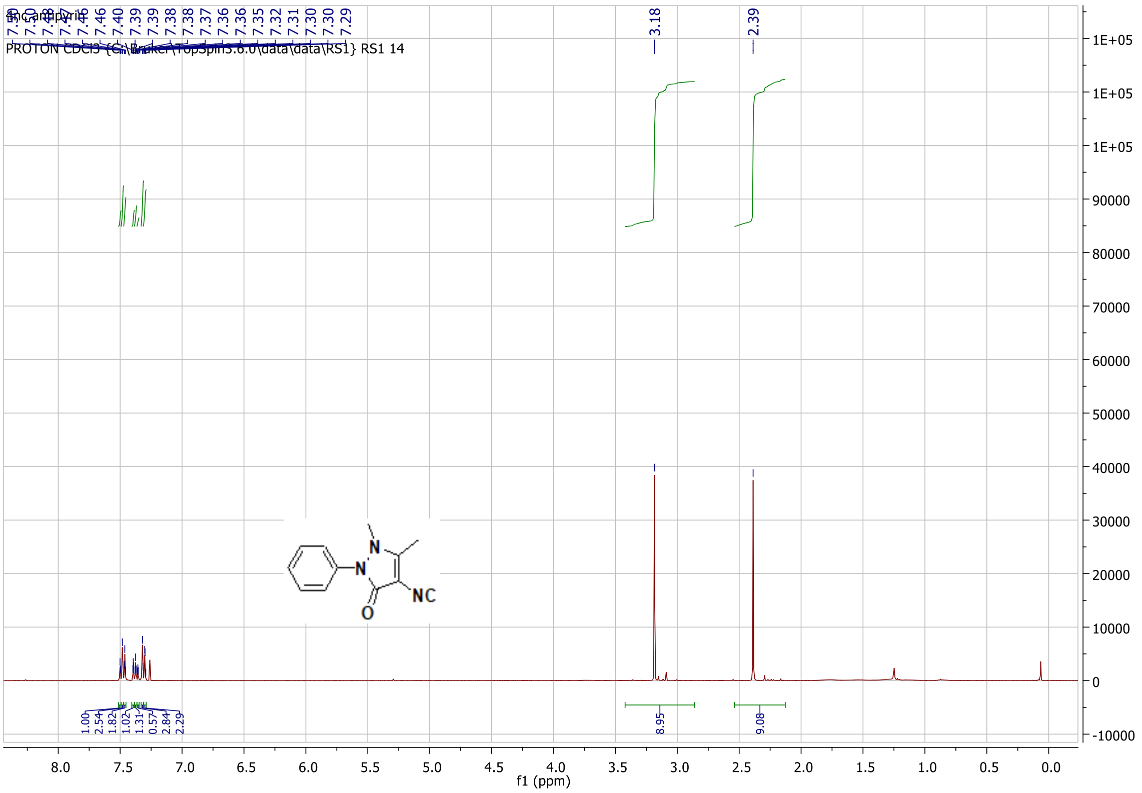

Figure S62. ^{1}H NMR spectrum (400 MHz, $CDCl_3$)

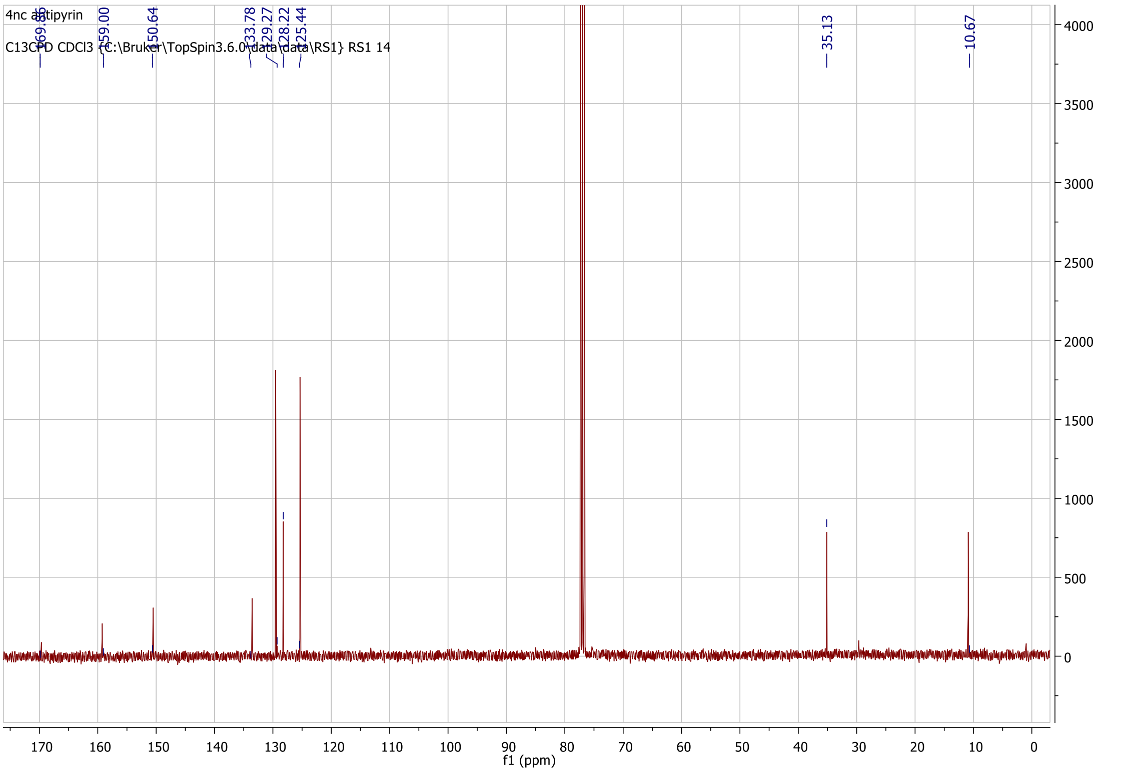

Figure S63. ^{13}C NMR spectrum (101 MHz, $CDCl_3$)

2.32. 1,2,4-trichloro-5-isocyanobenzene

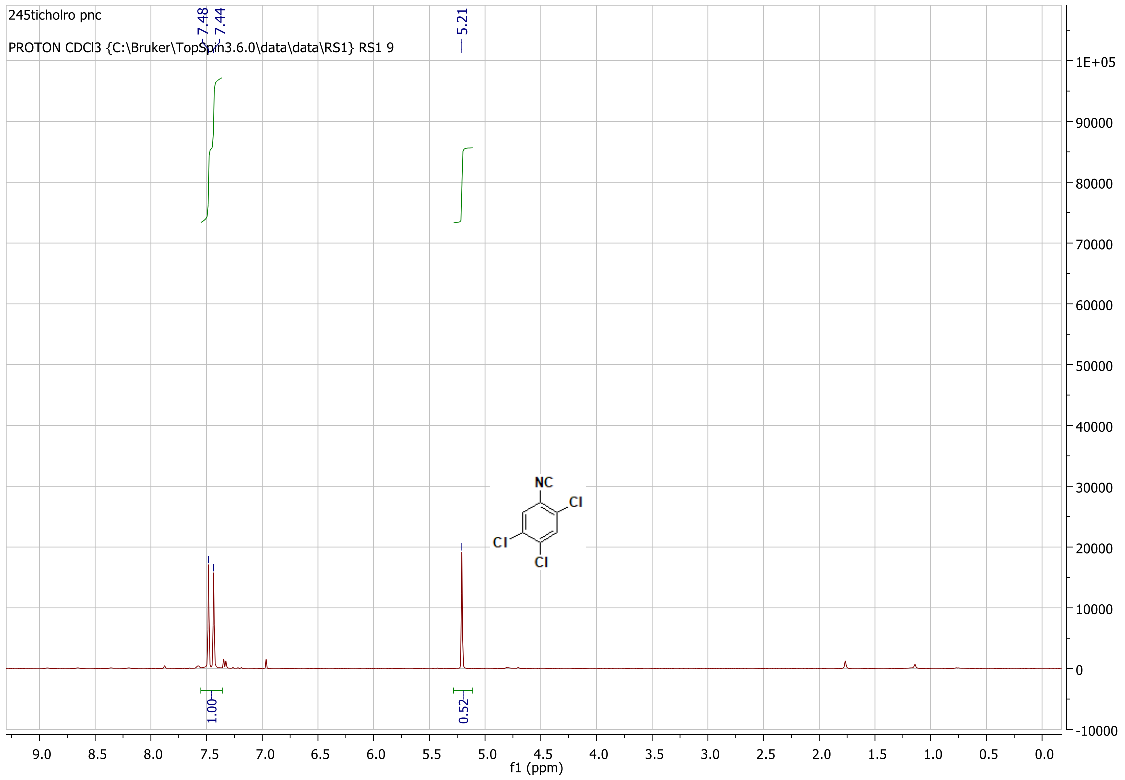

Figure S64. ^{1}H NMR spectrum (400 MHz, $CDCl_3$)

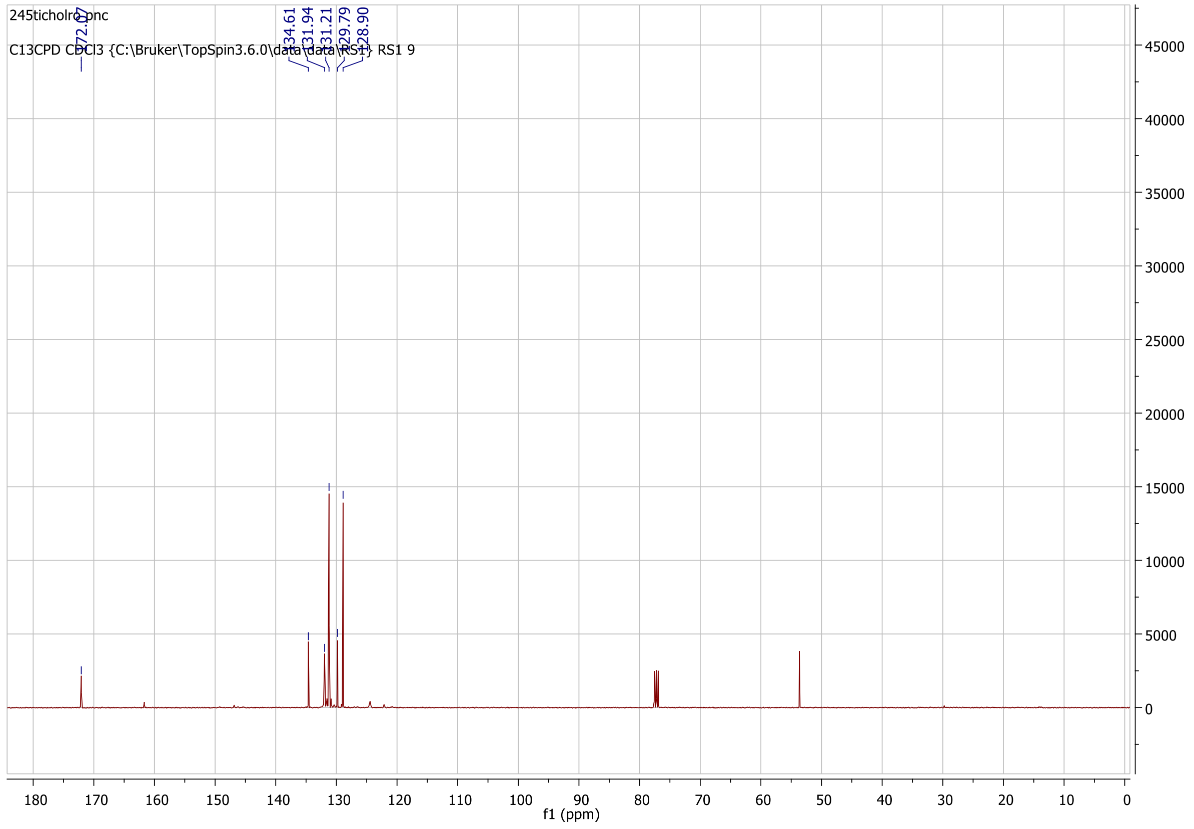

Figure S65. ^{13}C NMR spectrum (101 MHz, $CDCl_3$)

2.33. 2-isocyanobenzenethiol

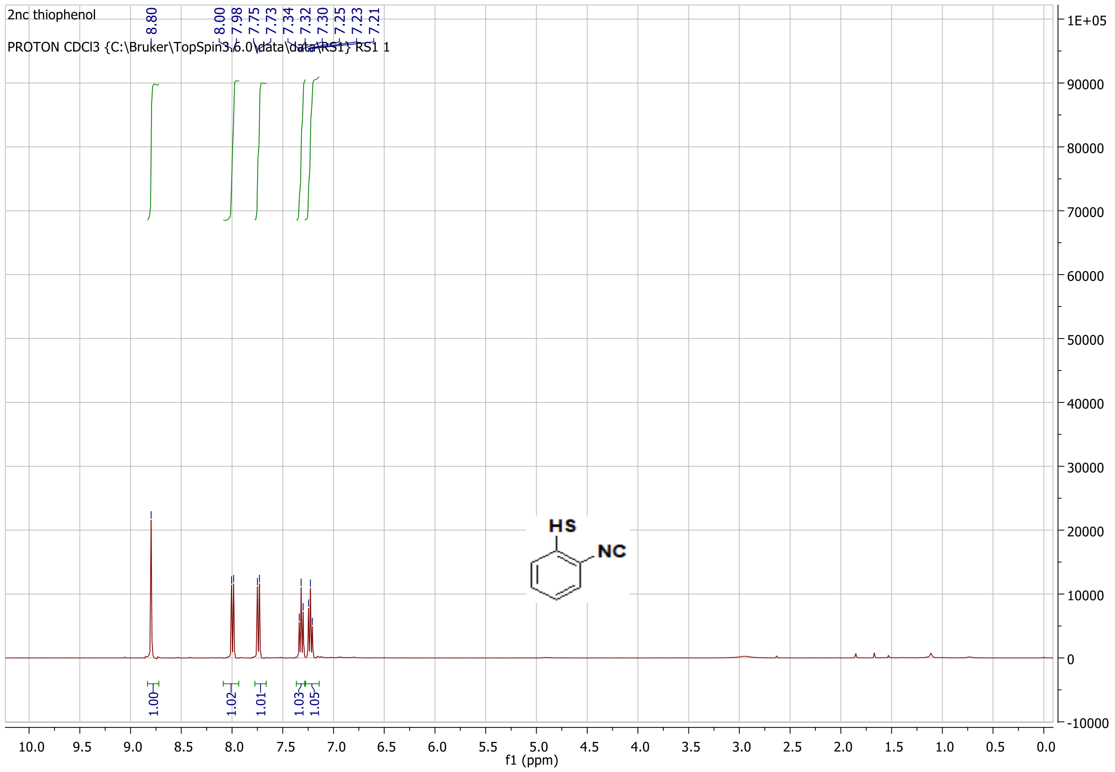

Figure S66. ¹H NMR spectrum (400 MHz, $CDCl_3$)

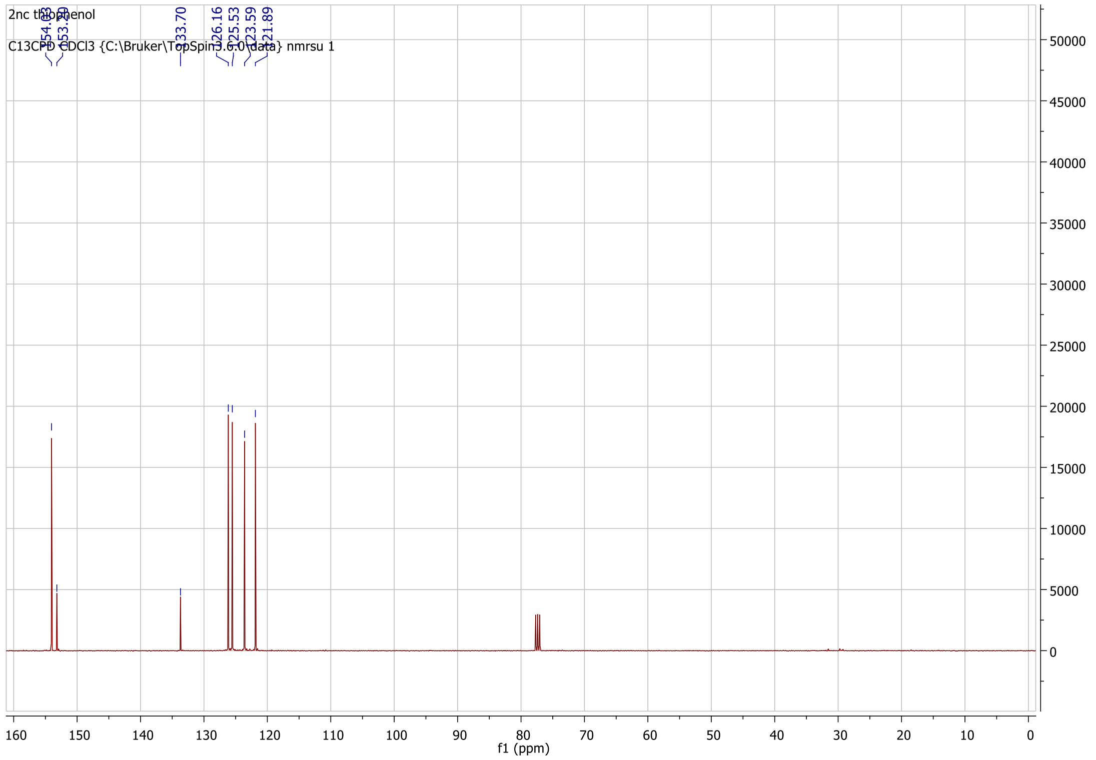

Figure S67. ¹³C NMR spectrum (101 MHz, $CDCl_3$)

2.34. 2-isocyano-1-methoxy-4-methylbenzene

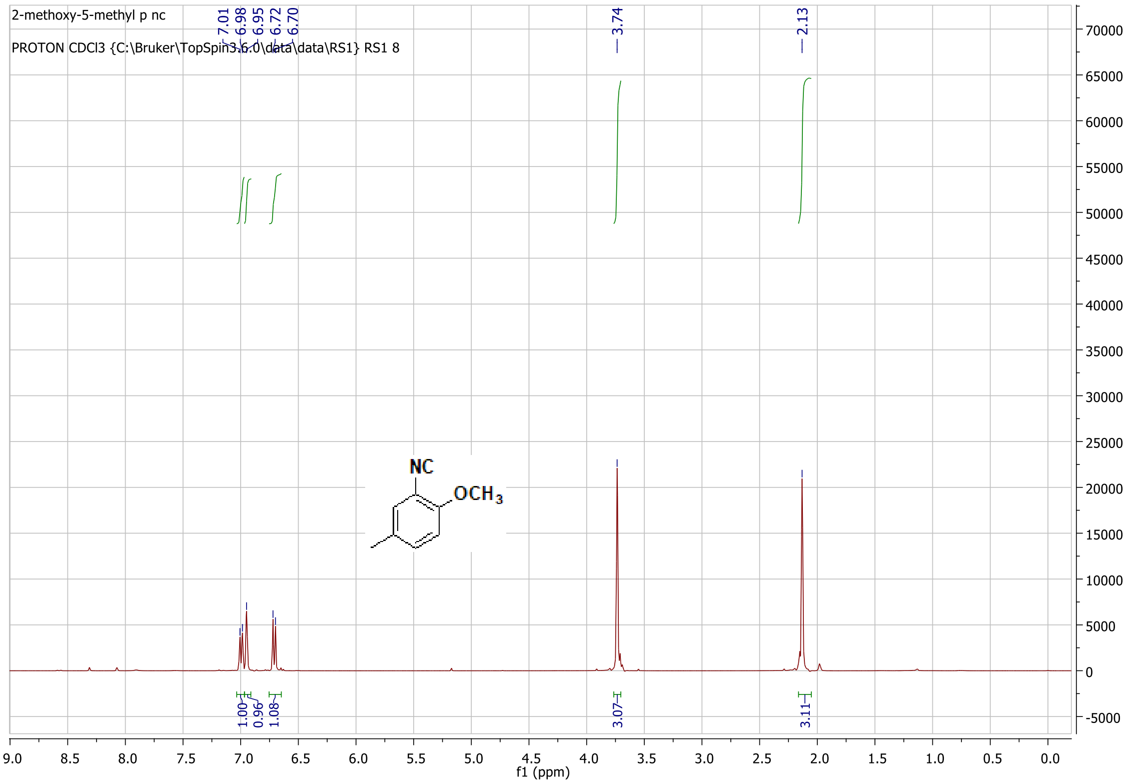

Figure S68. ^{1}H NMR spectrum (400 MHz, $CDCl_3$)

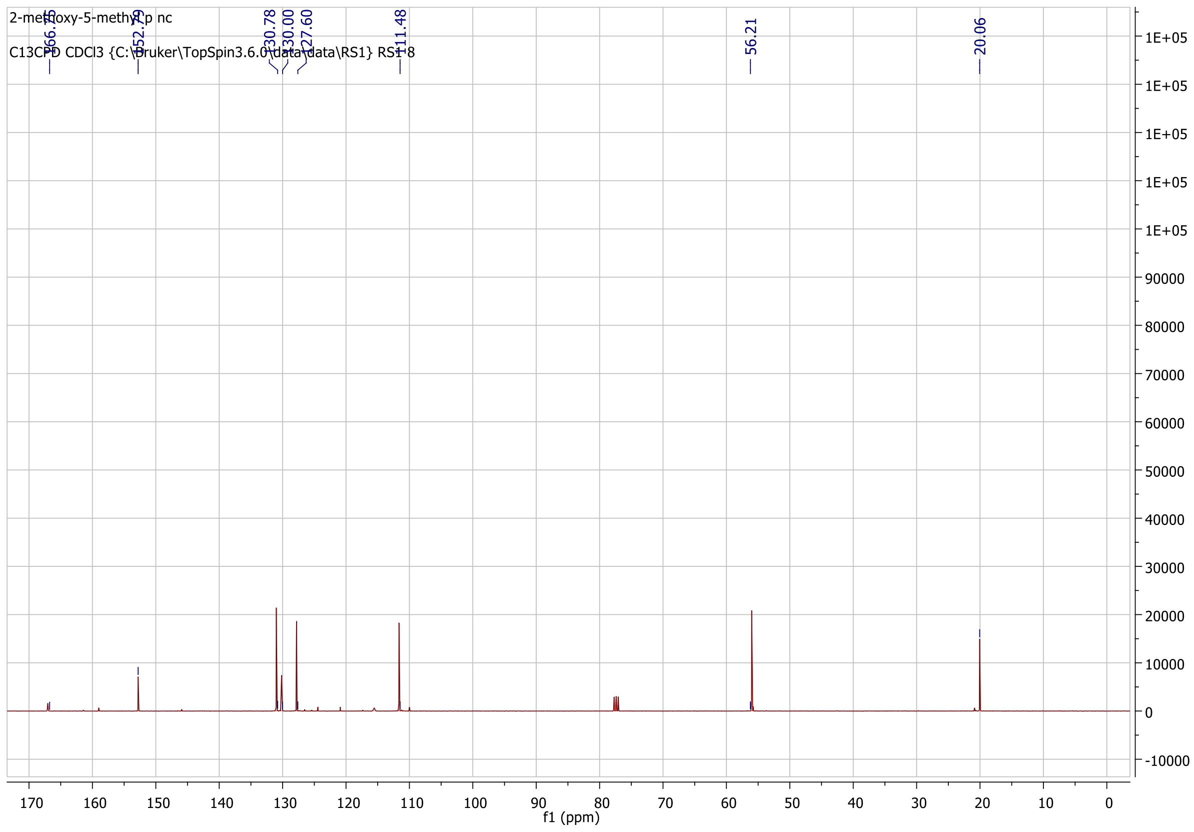

Figure S69. ^{13}C NMR spectrum (101 MHz, $CDCl_3$)

Passerini product

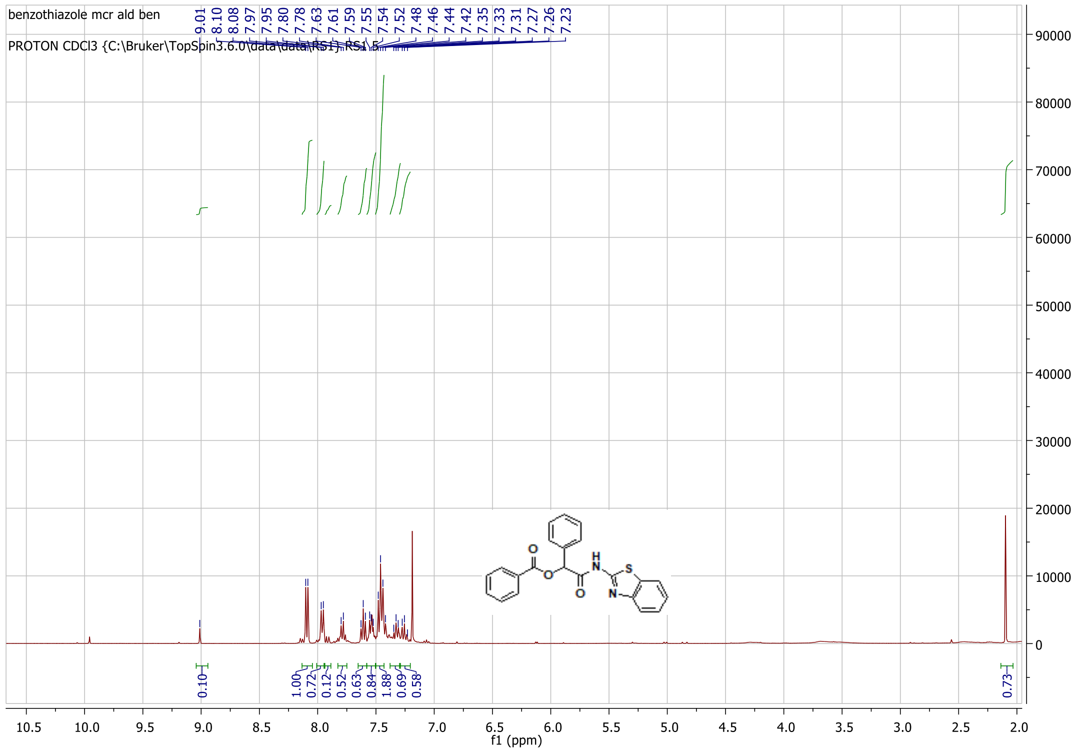

Figure S70. ^{1}H NMR spectrum (400 MHz, $CDCl_3$)

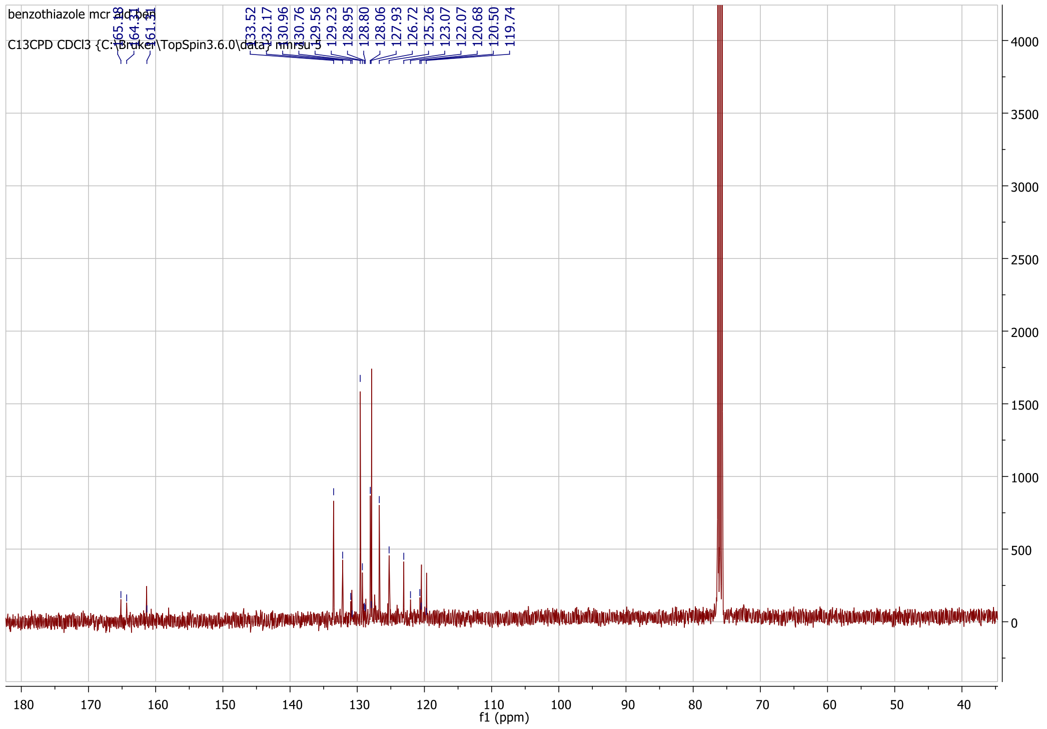

Figure S71. ^{13}C NMR spectrum (101 MHz, $CDCl_3$)

3. Comparison of various parameters for 100 mmol synthesis of various optimized conditions

Here we compare different parameters of literature reported procedures for the optimized conditions for isocyanides synthesis. However, not all procedures were reported on a 100 mmol scale: 1. Waibel-2020: 5 mmol; 2. Wang-2015: 1 mmol; 3. Domling-2009: 100 mmol; 4. Patil-2020: 100 mmol; 5. our method: 2 mmol. Thus, we calculate these values for solvents amount from literature by scaling them to 100 mmol scale. Moreover, we count the number of operations, reaction time, amount of dichloromethane used, aqueous waste generated during the reaction until isolating the pure product in all procedures.

References	**Waibel 2020** [18]	**Wang 2015** [27]	**Domling 2009** [30]	**Patil 2020** [19]	**This method**
Solvent used (ml)	200	900	100	50	**0**
Aq. Waste generated (ml)	500	3000	300	0	**0**
% Yield	97	90	65	97	**98**
Reaction time (min)	120	60	300	12	**5**
Number of Operations	9	7	9	3	**3**
E-Factor	7.41	18.4	50.5	8.4	**5.5**

Calculation of E-factor

E-factors were determined according to Sheldon, taking into account all reactants, including the reaction solvent and reagents. The amount of silica gel used for column chromatography was not considered when calculating the E-factors because it is rarely reported. However, if we consider the normal amount of silica gel utilized for other well-known methods of synthesis in the literature, E-factors will skyrocket.

E-factor = Kg (waste)/Kg (product)

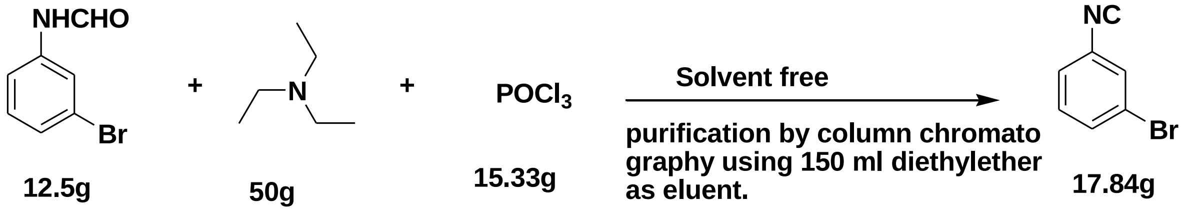

Amount of total amount of Reactants:

Mass (1-bromo-3-isocyanobenzene) = 12.5 g

Mass (Triethylamine) = 50 g

Mass ($POCl_3$) = 15.33 g

Mass (Diethyl ether (assuming 90% recovery)): 150 ml x (0.706 g/ml) x 10% = 10.59 g

Total mass of Reactants waste: (12.5 g + 50.5 g + 15.33 g + 3.7g + 10.59 g =
98.62 g

Amount of product = 17.84 g

E-Factor = Amount of waste/Amount of product = 98.62 g/17.84 g = 5.5

E-factor = 5.5

Process Mass Intensity Calculation (PMI).

To further quantify improvements to the efficiency and sustainability of our protocol PMI was calculated. PMI is calculated by dividing the total quantity of raw materials (kg) that go into synthesizing a product, including silica gel, by the quantity of final product produced (kg).

PMI= Total quantity of raw materials/ Amount of Product.

Total quantity of raw materials:

Mass (1-bromo-3-isocyanobenzene) = 12.5 g

Mass (Triethylamine) = 50 g

Mass ($POCl_3$) = 15.33 g

Silica gel (70–230 mesh size) =2.0 g

Mass (Diethyl ether (assuming 90% recovery)): 150 ml x (0.706 g/ml) x 10% = 10.59 g

Total mass of Reactants waste: (12.5 g + 50.5 g + 15.33 g + 3.7g + 10.59 g +2.0 g =
100.62 g

Amount of product = 17.84 g

PMI= Total quantity of raw materials/ Amount of Product. 100.62/17.84

PMI = 5.6
